# Guideline for management of septic arthritis in native joints (SANJO)

**DOI:** 10.5194/jbji-8-29-2023

**Published:** 2023-01-12

**Authors:** Christen Ravn, Jeroen Neyt, Natividad Benito, Miguel Araújo Abreu, Yvonne Achermann, Svetlana Bozhkova, Liselotte Coorevits, Matteo Carlo Ferrari, Karianne Wiger Gammelsrud, Ulf-Joachim Gerlach, Efthymia Giannitsioti, Martin Gottliebsen, Nis Pedersen Jørgensen, Tomislav Madjarevic, Leonard Marais, Aditya Menon, Dirk Jan Moojen, Markus Pääkkönen, Marko Pokorn, Daniel Pérez-Prieto, Nora Renz, Jesús Saavedra-Lozano, Marta Sabater-Martos, Parham Sendi, Staffan Tevell, Charles Vogely, Alex Soriano, the SANJO guideline group

**Affiliations:** 1 Dept. of Orthopaedic Surgery and Traumatology, Aarhus University Hospital, Aarhus, Denmark; 2 Dept. of Orthopedic Surgery, University Hospitals Ghent, Ghent, Belgium; 3 Dept. of Infectious Diseases, Hospital de la Santa Creu i Sant Pau, Barcelona, Spain; 4 Dept. of Infectious Diseases, Centro Hospitalar do Porto, Porto, Portugal; 5 Dept. of Internal Medicine, Hospital Zollikerberg, Zürich, Switzerland; 6 Dept. of Prevention and Treatment of Wound Infection, Vreden National Medical Research Center of Traumatology and Orthopedics, St. Petersburg, Russia; 7 Dept. of Clinical Microbiology, University Hospitals Ghent, Ghent, Belgium; 8 Dept. of Internal Medicine, IRCCS Ospedale Galeazzi Sant'Ambrogio, Milano, Italy; 9 Dept. of Clinical Microbiology, University Hospital Oslo, Oslo, Norway; 10 Dept. of Septic Orthopedic Surgery and Traumatology, BG Klinikum Hamburg, Hamburg, Germany; 11 Dept. of Infectious Diseases, Attikon University General Hospital, Athens, Greece; 12 Dept. of Orthopaedic Surgery and Traumatology, Aarhus University Hospital, Aarhus, Denmark; 13 Dept. of Infectious Diseases, Aarhus University Hospital, Aarhus, Denmark; 14 Dept. of Orthopaedic Surgery Lovran, University of Rijeka, Rijeka, Croatia; 15 Dept. of Orthopaedic Surgery, University of KwaZulu-Natal, Durban, South Africa; 16 Dept. of Orthopaedics, P.D. Hinduja Hospital and Medical Research Centre, Mumbai, India; 17 Dept. of Orthopaedic and Trauma Surgery, OLVG Amsterdam, Amsterdam, the Netherlands; 18 Dept. of Orthopaedics and Traumatology, Turku University Hospital, Turku, Finland; 19 Dept. of Infectious Diseases, Ljubjana University Medical Center, Ljubjana, Slovenia; 20 Dept. of Orthopaedic Surgery and Traumatology, Hospital del Mar, Barcelona, Spain; 21 Dept. of Infectious Diseases, Bern University Hospital, Bern, Switzerland; 22 Dept. of Pediatric Infectious Diseases Unit, Gregorio Marañón Hospital, Madrid, Spain; 23 Dept. of Orthopaedic Surgery and Traumatology, Hospital Clínic, Barcelona, Spain; 24 Dept. of Infectious Diseases, University Hospital of Basel, Basel, Switzerland; 25 Dept. of Infectious Diseases, Karlstad Hospital and Centre for Clinical Research, Karlstad, Sweden; 26 Dept. of Orthopedic Surgery, University Medical Center Utrecht, Utrecht, the Netherlands; 27 Dept. of Infectious Diseases, Hospital Clínic, Barcelona, Spain; 28 Institute for Infectious Diseases, University of Bern, Bern, Switzerland; ℹ Members of the Steering Committee for the EBJIS Guideline Project on SANJO; ★ shared first authorship; ☆ shared last authorship; ➕ A full list of authors appears at the end of the paper

## Abstract

This clinical guideline is intended for use by orthopedic surgeons and physicians who care for patients with possible or documented septic arthritis of a native joint (SANJO). It includes evidence and opinion-based recommendations for the diagnosis and management of patients with SANJO.

## Supplement

10.5194/jbji-8-29-2023-supplement The supplement related to this article is available online at: https://doi.org/10.5194/jbji-8-29-2023-supplement.

## Figures and Tables

**Figure 1:**
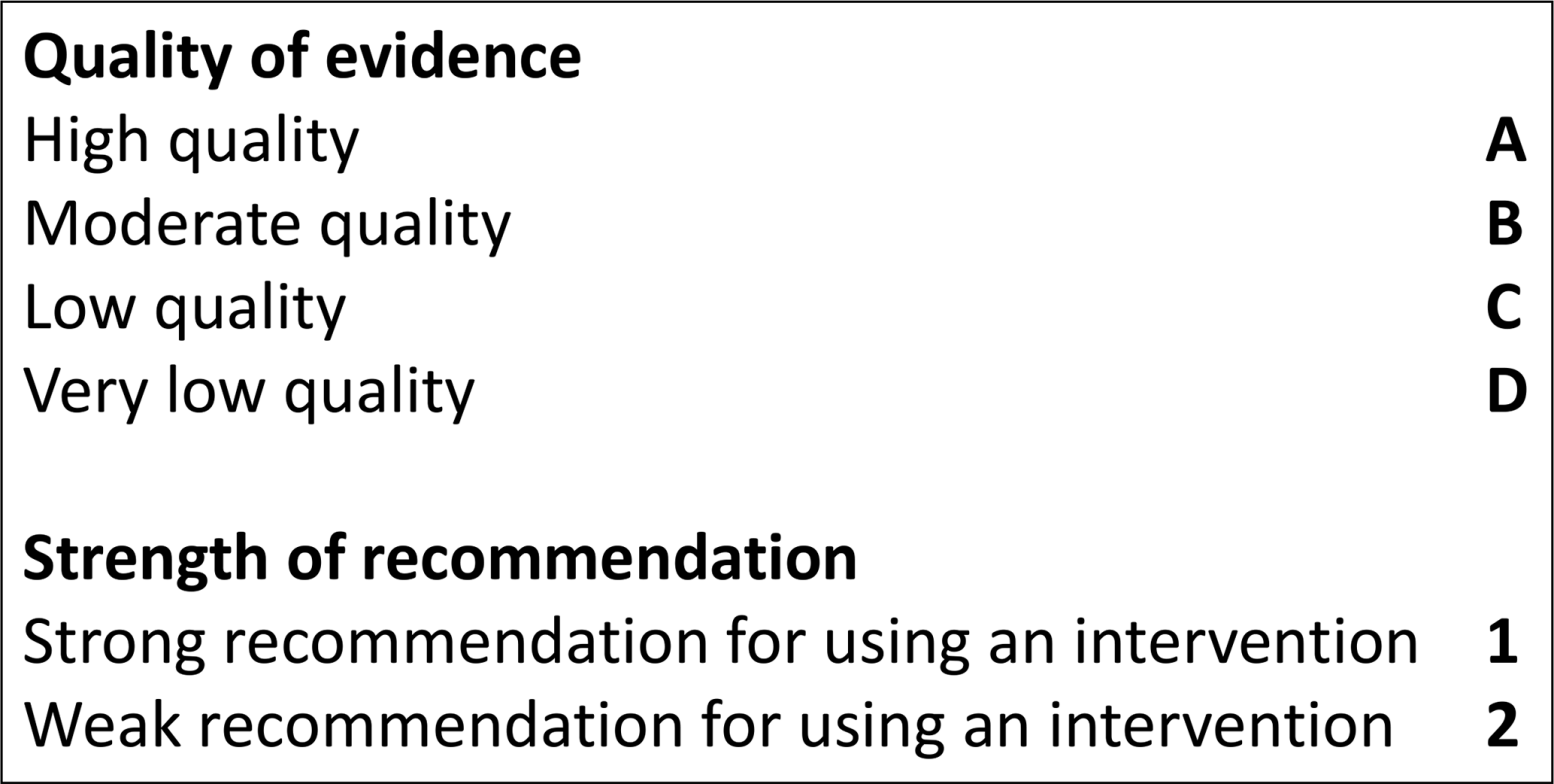
Representations of quality of evidence and strength of recommendations (Guyatt et al., 2008).

**Figure 2:**
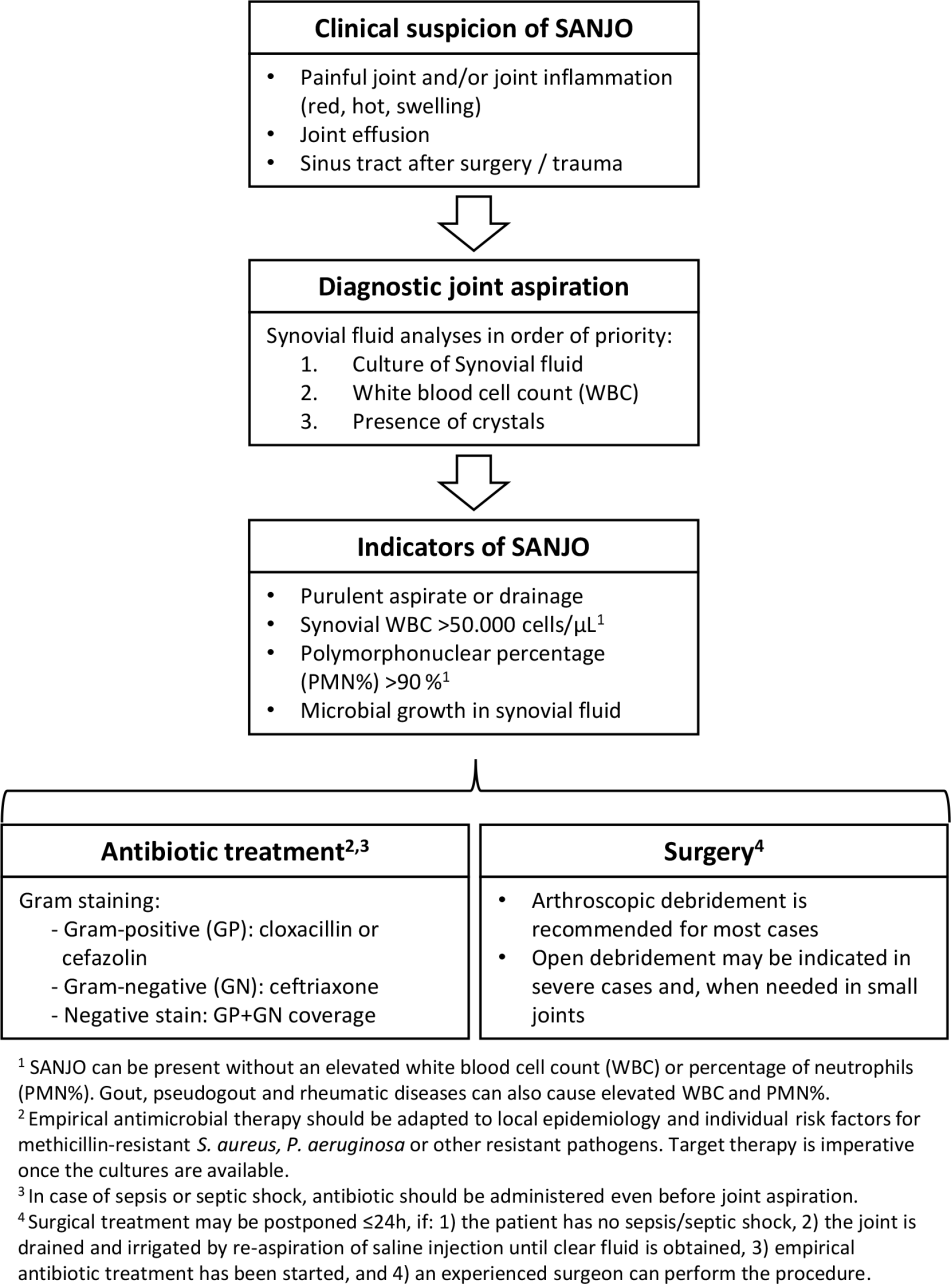
Management of septic arthritis in native joints (SANJO).

**Table 1:** Severity stages of SANJO based on the intraarticular appearance by either open or arthroscopic evaluation (Stutz et al., 2000).

Gächter	Arthroscopic appearance
Stage I	Synovitis, turbid fluid, possible petechiae
Stage II	Highly inflammatory synovitis, clumps of fibrin, pus
Stage III	Thickening of the synovial membrane with adhesions and pouch formation
Stage IV	Pannus formation, proliferation of aggressive synovitis, radiographically visible changes, subchondral erosions

## Data Availability

The work group reports from the Appendix are available in the Supplement.

## Executive summary

: the European Bone and Joint Infection Society (EBJIS) has initiated this interdisciplinary collaborative project to create a concise evidence-based clinical guideline for the management of SANJO (septic arthritis of a native joint). : a steering committee identified 25 clinical dilemmas related to SANJO and grouped these into nine categories as reported below. For each category, a work group of international experts was recruited amongst members of the European Bone and Joint Infection Society (EBJIS), and for the chapter on SANJO after ACL reconstruction, the recommendations were made in cooperation with appointed members of the European Society for Sports Traumatology, Knee Surgery and Arthroscopy (ESSKA). The work groups followed a predefined process of systematic literature search, weighting in the strength of recommendations and quality of evidence (Fig. 1). A detailed description of the methods, background, and evidence reporting for each recommendation with references can be found in the associated work group report (see the Appendix).

## GRADE: Grading of Recommendations Assessment, Development and Evaluation

### Overall recommendations

The diagnosis of septic arthritis in native joints (SANJO) is mainly based on aspiration of joint fluid, which initially is analysed for synovial leucocyte count, but most important, for bacterial identification. Except for patients with signs of sepsis, empirical antibiotic treatment should await diagnostic sampling of joint fluid to avoid false negative culture results. Arthroscopic lavage (with synovectomy, depending on the clinical stage) is recommended for SANJO particularly in larger joints, although open revision could be considered in cases with synovial membrane adhesions or in the presence of cartilage or bone damage. Empirical antibiotic treatment should be selected considering the most likely pathogens and targeted according to the results from microbiology laboratory. Joint mobilization to avoid contracture should be started as soon as possible when infection is under control and after drains have been removed. Careful postoperative evaluation should reveal early signs of treatment failure, which indicates repeated surgical revision. This guideline also includes specific considerations for SANJO: (1) after reconstruction of the anterior cruciate ligament, (2) in tuberculous arthritis, and (3) in pediatric population.

*Guidelines cannot always account for individual variation among patients. They are not intended to replace physician judgment with respect to particular patients or special clinical situations.*

## Recommendations for diagnostic approach

1.: **Are clinical parameters important in the evaluation of a patient with an inflamed painful joint?** *Recommendations:* – A high suspicion of SANJO should be kept in mind in any patient with a painful and/or inflamed joint (redness, hot, swelling, synovial effusion, and/or purulent drainage) with or without a fever (B1). Although a thorough patient history and examination may contain essential information, no clinical parameters can exclude or confirm SANJO (B1). – Clinical parameters should also be used to identify patients with concomitant sepsis or septic shock, requiring immediate attention and rapid surgical and medical treatment (B2). – The diagnosis of tuberculosis (TB) arthritis should be considered in patients with a subacute or chronic course of arthritis (weeks to months or even years) – especially for patients living in or previously living in endemic areas or with a prior history of TB (C1).
2.: **Does normal blood C-reactive protein (CRP) exclude septic arthritis?** No blood tests have either the sensitivity or the specificity to confirm or exclude SANJO. *We suggest* CRP kinetics be used to support the diagnosis and monitor clinical response to treatment (D2).
3.: **When is aspiration of synovial fluid indicated?** *We recommend* aspiration of synovial fluid should be performed as quickly as possible when SANJO is suspected (B1).
4.: **Which analyses should be made on synovial fluid?** When analysing synovial fluid in the setting of SANJO, several factors must be considered. The volume of aspirated synovial fluid may limit which and how many analyses are possible. Also, capacity and accessibility at the clinical laboratory may be another limitation. *Recommendations*: – Synovial fluid should be analysed for bacterial identification (see microbiological methods section), white blood cell count including polymorphonuclear (PMN) percentage, and presence of crystals (in that priority order if the quantity of liquid is not enough for all of them) (B2). – Additional investigation, such as analysis of synovial lactate and synovial glucose may also be considered (C2). – Point of care testing with leucocyte esterase and or glucose strips may add useful bedside information (D2).
5.: **Can certain levels of synovial leukocyte and/or differential count confirm/exclude septic arthritis?** *Recommendations:* – A synovial white blood cell count of > 50 000 cells µL ${}^{-1}$ is suggestive of SANJO, but alone is not sufficient for the diagnosis (B2). – Low synovial white blood cell count ( < 25 000 cells µL ${}^{-1}$ ) decreases post-test probability, but it cannot exclude SANJO (B2). – The previously mentioned cutoffs may not be applicable in immunosuppressed patients (D2). – Gout and pseudogout may also increase levels of white blood cell count in the joint. Nevertheless, the presence of crystals does not rule out SANJO (D2). – It is not possible to give a recommendation for a particular percentage of synovial PMN to diagnose or rule out SANJO, although higher percentages make the diagnosis of SANJO more probable (D2).
6.: **What is the role of imaging in patients with suspected septic arthritis?** *Recommendations:* – Plain joint radiographs are useful in screening for pre-existing conditions (fracture, osteomyelitis, osteoarthritis, implants, etc.). Radiographs may also serve as reference for future monitoring (B2). – Ultrasound, computed tomography (CT), or magnetic resonance imaging (MRI) may be helpful to detect joint effusion and surrounding abscesses (B2). – Ultrasound, fluoroscopy, or CT may be helpful to guide a joint aspiration for not-easily accessible joints (B2). – The usefulness of MRI is limited by accessibility, but MRI may be required for diagnosis in specific joints (e.g. such as the sacroiliac joint) and/or to detect adjacent osteomyelitis (B2).

### Recommendations for microbiological methods

7.: **Which microbiological samples give the best culture yield?** Cornerstone for diagnosing septic arthritis is the detection of the causative pathogen in synovial fluid or in synovial biopsies. Blood cultures can also be positive. *We recommend* obtaining the following samples: – Synovial fluid for microbiological culture (B1). – At least two sets (aerobic and anaerobic bottle for each set) of blood cultures in febrile patients or when bacteremia or sepsis is suspected (C2). – Synovial biopsies (in case of surgical intervention and/or suspicion of TB) for microbiological cultures and histopathological analysis (D2).
8.: **Which techniques are recommended for joint aspiration?** *Recommendations:* – A strict aseptic joint aspiration technique is important to avoid contamination of the joint and the sample material. The needle puncture site needs to be disinfected and the skin should be completely dry before inserting the needle (D1). – Joint puncture through a subcutaneous abscess must be avoided, and if possible, puncture via a route with overlying cellulitis should be avoided (D2). – In case of a dry tap, the needle may be wrongly positioned (outside the joint capsule). Guidance from ultrasound, fluoroscopy, or CT may be helpful to document intraarticular needle positioning (C1). – Injection of saline to increase the culture yield is not recommended (D1). – Development of a standard operating procedure might assist in improving diagnostic reliability (D1).
9.: **How should synovial fluid be analysed in the microbiology laboratory?** *Recommendations:* – Synovial fluid ( ≥ 1 mL) should always be sent for culture in sterile tubes (B1). In case fluid is left over after initial sampling for culture, white blood cell count, and crystal analysis, it is recommended to inoculate the remaining synovial fluid into blood culture bottles (C2). – Performing Gram staining on synovial fluid is recommended despite its limited sensitivity; given its excellent specificity, it can provide early proof of infection and help guiding empirical treatment (B2). – Synovial fluid from a sterile tube should be plated on agar plates and enrichment broth (thioglycolate) as an alternative or in addition to inoculation in blood culture bottles (D2). – Incubation time is recommended between 5 and 7 d according to local practice (D2). – In cases with high suspicion of infectious arthritis but negative culture results after 7 d, prolonged cultivation for up to 10–14 d should be considered (C2). – In patients taking antibiotics at the time of synovial fluid aspiration, when difficult-to-culture pathogens are suspected, or in case of negative culture results despite high suspicion of SANJO, molecular polymerase chain reaction (PCR) technology using synovial fluid are recommended (C2). – In patients with suspicion of TB arthritis, a sample of synovial fluid and synovial biopsies should be analysed for acid-fast bacilli stain, mycobacterial culture, and nucleic acid amplification test (C1).

### Recommendations for initial surgery

10.: **What is the indication for either initial closed (arthroscopic) or open (arthrotomy) surgery?** Invasive treatment (open surgery, arthroscopy, or arthrocentesis) is necessary to wash out toxins and reduce both the bacterial load and intraarticular pressure. Even though (serial) joint aspiration seems to have a role, we recommend surgical debridement for SANJO, especially in larger joints. Based on the limited evidence in the studies available, it appears that (if logistically and surgically possible) an arthroscopic debridement is an adequate option as initial surgical management in patients with Gächter stage I, II, and probably also III septic arthritis (Table 1). In patients with Gächter stage III and definitely in stage IV, an open debridement can be considered (B2). However, the Gächter classification has still to be clinically validated for management of septic arthritis.

11.: **What is the optimal timing of surgery; immediate (< 12 h) or delayed (12–48 h)? ** Even though SANJO is an indication for acute treatment, there is limited evidence that a 24–48 h delay (in the patient without sepsis) could be accepted without negative impact on infection control. It must be noted that there is some evidence to suggest that delay of surgery beyond 24 to 48 h increases the need for repeat debridement (C2). None of the studies investigated the long-term impact of surgical delay on joint degeneration. This topic was presented and discussed during a session at the 2021 annual meeting of EBJIS, where consensus was in favor of the following statement (D2): *We suggest* surgical treatment for the patient without sepsis or septic shock with SANJO can be postponed but no longer than 24 h under the following conditions: - the joint has already been preliminarily treated by articular needle aspiration and irrigation (saline injection under sterile conditions, and re-aspiration until clear fluid is obtained), - *and* empirical antibiotic treatment has been initiated, - *and* an experienced surgeon can perform the procedure.

### Recommendations for empirical antibiotic treatment

12.: **What is the indication for empirical antibiotic treatment?** *We suggest* the antibiotic treatment for suspected SANJO should be started *after* aspiration of synovial fluid for laboratory analysis and obtaining blood cultures (D1): – In cases with sepsis or septic shock, empirical antibiotic treatment must be started as soon as possible according to institutional sepsis guidelines (B1). – Antibiotics can be stopped in case of reasonable alternative diagnosis (D2).
13.: **What is the strategy for selecting empirical antimicrobial treatment?** *We suggest* empirical treatment be selected according to local epidemiology/resistance patterns. Further factors to consider are (D2) the following: – The result of Gram staining (Gram-positive or Gram-negative) and morphology (cocci (grapes or chains) or bacilli) – The presence of risk factors for methicillin-resistant *Staphylococcus aureus* (MRSA) or multidrug-resistant Gram-negative bacilli including *Pseudomonas aeruginosa* and cephalosporin-resistant Enterobacterales. – In the absence of risk factors for resistant microorganisms and with negative Gram stain, the authors recommend a combination of semisynthetic penicillin (e.g. cloxacillin, cefazoline) plus ceftriaxone or monotherapy with amoxicillin–clavulanic acid.
14.: **When can oral antibiotic administration replace intravenous administration?** There are no clinical trials that evaluate either the total duration of antimicrobial treatment or the duration of intravenous and oral antibiotic treatment in adults with SANJO. *We suggest* de-escalating antibiotic treatment according to the susceptibility pattern of the isolated pathogen, to maintain intravenous administration for 1–2 weeks, and switch to oral treatment as soon as the clinical signs and symptoms of infection (fever, redness, warm, swelling, pain) and blood biomarkers (leucocyte count and C-reactive protein) indicate a satisfactory progress. The selection of oral antibiotic should be based on the in vitro activity, oral bioavailability, and the diffusion to the synovial fluid. *We suggest* a treatment duration of oral antibiotics of 2–4 weeks (D2).

### Recommendations for mobilization after surgical treatment

15.: **What are the strategies of joint mobilization?** In SANJO, there is not enough evidence to recommend one mobilization strategy over another. Previous uncontrolled observational studies have shown that prolonged immobilization following surgical intervention leads to limitation of knee motion, atrophy, stiffness, and contractions. Conversely, animal and basic studies have demonstrated the beneficial effects of continuous passive motion. *We suggest* mobilization to be started as soon as possible once infection is under control and after drains have been removed and surgical wounds closed (D2).

### Recommendations for evaluation of outcomes and treatment failure

16.: **What are the most important signs of treatment failure during treatment for SANJO?** Based on the limited evidence available in the literature and with expert-based input, *we recommend* looking for the following signs indicating treatment failure (D2): – *Clinical signs and symptoms.* Persistent pain and/or local signs of inflammation (including presence of purulent discharge) and/or systemic signs of infection and/or deteriorating joint function. – *Blood inflammatory biomarkers.* CRP and white blood cell count not decreasing or rather increasing. – *Synovial fluid at re-aspiration in case of poor clinical progression.* Elevated or not decreasing white blood cell count and percentage polymorphonuclear leucocytes, persistently positive microbial cultures. The clinician should primarily monitor the clinical data as well as the blood and synovial fluid biomarkers suggesting treatment failure. In case of treatment failure, imaging modalities may be able to demonstrate adjacent tissue damage, periarticular abscesses, and/or osteomyelitis.
17.: **Which key elements should be addressed when evaluating the treatment outcome of native joints after septic arthritis?** Based on the limited evidence available in the literature and with expert-based input, we *recommend* considering at least the following when evaluating treatment outcome (D2): a. Eradication of the joint infection. b. Parameters related to remaining joint function. c. Related mortality and overall mortality at 30 d.

### Recommendations for management of septic arthritis after reconstruction of the anterior cruciate ligament (ACL-R)

18.: **What are the clinical signs and symptoms that should raise suspicion of infection after ACL-R?** Suggestive signs and symptoms are delayed range of motion recovery, increased warmth or swelling, wound drainage, and arthrofibrosis, as well as unusual pain and systemic symptoms such as fever and malaise. Confirmative signs are purulent discharge/aspirate, sinus tract communication with the joint, and the presence of intraarticular pus (B2).
19.: **Is surgical treatment necessary for an infection after ACL-R? Which type of surgery is called for?** Surgical treatment is necessary to wash out toxins and reduce both the bacterial load and intraarticular pressure. *We recommend:* – Performing arthroscopic debridement as the primary therapeutic option in every patient (C2). – Arthroscopic inspection and debridement of the joint to evaluate the ACL-graft, achieve early infection control, and promote faster recovery in comparison to open surgery (B1). – Arthroscopic debridement should be performed as soon as clinical suspicion is raised in cases with acute symptoms or in the early postoperative setting, even if the microbiological results are still pending (B1). – In the rare case of inoperability, repeated needle aspiration might be an alternative (C2).
20.: **How many arthroscopic procedures should be performed for an infection after ACL-R? ** *We suggest:* – Additional debridement after the first one is indicated if the clinical course is not favorable. Unfavorable determinants include increasing pain, fever, and/or persistent or secondarily increased CRP without any other explanation (e.g. nosocomial infection), a persistent discharge from the portal, or persistent local signs of inflammation (D2). – If progress is not favourable even after a third debridement, removal of the graft and hardware should be considered (D2). – Magnetic resonance imaging may help in identifying the cause of persistent infection (D2).
21.: **In situations where the graft and hardware has been removed, when can the new ACL-R be performed?** We *suggest* that graft reimplantation be performed after at least 6 weeks treatment of infection in selected cases in cases of graft and hardware removal (D2).
22.: **What is the optimal antibiotic treatment duration for ACL-R-infections?** *We suggest:* – One to 2 weeks of intravenous treatment followed by oral treatment for another 4–5 weeks, preferably with bactericidal agents with good oral bioavailability and bone penetration, as well as biofilm-activity, especially if avascular tissue and fixation devices remain in situ (D2). – The precondition for switching to oral treatment is a good clinical response and a downward CRP trend (D2).

### Recommendations for management of septic arthritis suspected of tuberculosis

23.: **What are the special considerations related to the treatment of SANJO caused by *Mycobacterium tuberculosis*?** *Recommendations:* – Early cases of TB arthritis should be treated with medical therapy alone (D2). – Surgical intervention should be avoided in the active phase of TB arthritis, and a debridement and synovectomy should only be considered in exceptional cases with large abscesses, significantly devitalized bone, or showing inadequate response to medical management (C1). – Patients with substantial joint destruction, ankylosis, deformity, significant loss of function, or chronic pain after TB arthritis may benefit from operative management with excisional arthroplasty or arthrodesis (D1). – Initial medical therapy for TB consists of a combination of four drugs including rifampin, isoniazid, ethambutol, and pyrazinamide for 2 months; ethambutol may be discontinued if susceptibility to the other three drugs is demonstrated (C1). – After the induction 2-month period, patients with drug-susceptible TB should continue with isoniazid and rifampin (C1). – We recommend a minimum regimen of 6 months for drug-susceptible TB, although some experts tend to favour longer durations of 9 or even 12 months (D1). – Treatment and duration should be supervised by an infectious disease expert (D1).

#### Recommendations for management of septic arthritis in children

24.: **Which special considerations are related to antimicrobial treatment of septic arthritis in children?** The traditional treatment of paediatric SANJO (p-SANJO) has consisted of long courses of antibiotics started intravenously and aggressive surgery. During the recent decade, a more conservative approach to surgery has been observed. *We recommend* to culture synovial fluid obtained by joint aspiration (B1). *We suggest* the following strategy for antibiotic therapy: – Empirical therapy should be initiated immediately once appropriate cultures are obtained (C1). – Empirical antibiotics for covering the most common pathogen (*S. aureus*); first-generation cephalosporin, anti-staphylococcal penicillin, or clindamycin (C1). – Consider specific coverage in the following situations: – *Children* < *5 years.* *Kingella kingae* should be covered (C1). – *Neonates.* Antibiotic treatment should also cover Enterobacterales. – *Unvaccinated children.* Consider coverage for *Haemophilus influenzae* and *Streptococcus pneumoniae* (C2). – Methicillin-resistant *S. aureus* in areas with more than 10 %–15 % prevalence in the community (C1). – The duration of targeted antibiotic therapy for non-complicated p-SANJO (i.e. early presenting cases, immunocompetent children with timely response to antibiotic therapy) is the following: – The minimum duration of intravenous antibiotic therapy for non-complicated p-SANJO is 2–4 d. – The duration of therapy for non-complicated p-SANJO is 2–3 weeks.
25.: **Which special considerations are related to surgical treatment of septic arthritis in children?** Controversy remains regarding the need for invasive procedures besides diagnostic sampling. *We suggest* considering a more conservative approach to surgical treatment of p-SANJO once arthrocentesis and irrigation has been performed (D2). Factors to consider are the following: – Surgical treatment should be considered with a longer duration of symptoms before presentation ( > 5 d), with pathogens difficult-to-treat (e.g. MRSA), or after failure of conservative treatment (e.g. lack of clinical progress even after 2–3 joint aspirations) (D2). – Arthroscopy provides visualization of the joint space and may be considered, depending on the local expertise (D2). – Arthrotomy may be considered in neonates (3–6 months) (D2).

## Appendix

The supplement related to this article is available online at: https://doi.org/10.5194/jbji-8-29-2023-supplement.

## Team list

Miguel Araújo Abreu, Yvonne Achermann, Natividad Benito, Svetlana Bozhkova, Liselotte Coorevits, Matteo Carlo Ferrari, Karianne Wiger Gammelsrud, Ulf-Joachim Gerlach, Efthymia Giannitsioti, Martin Gottliebsen, Nis Pedersen Jørgensen, Sebastian Kopf, Tomislav Madjarevic, Leonard Marais, Aditya Menon, Dirk Jan Moojen, Jeroen Neyt, Markus Pääkkönen, Marko Pokorn, Daniel Pérez-Prieto, Nora Renz, Jesús Saavedra-Lozano, Marta Sabater-Martos, Parham Sendi, Alex Soriano, Staffan Tevell, and Charles Vogely.
