# Supplementary material for: Guideline for management of septic arthritis in native joints (SANJO)
Source: J Bone Jt Infect. 2023 Jan 12;8(1):29–37. doi: 10.5194/jbji-8-29-2023 (PMC9901514; doi:10.5194/jbji-8-29-2023)
Supplement: The supplement related to this article is available online at: https://doi.org/10.5194/jbji-8-29-2023-supplement. [file jbji-8-29-supplement.zip › Workgroup report 1, diagnosis of SANJO.pdf]

EBJIS guideline Workgroup 1: Diagnostic work-up and criteria

Authors

Nis Pedersen Jørgensen M.D., Ph.D
Department of Infectious Diseases, Aarhus University Hospital, Denmark

Karianne Wiger Gammelsrud M.D., Ph.D
Department of Clinical Microbiology, University Hospital Oslo, Norway

Staffan Tevell M.D., Ph.D
Department of Infectious Diseases, Karlstad Central Hospital, Centre for Clinical Research and Education, Värmland County Council, Karlstad, and School of Medical Sciences, Faculty of Medicine and Health, University of Örebro, Sweden

Background and aim of clinical application

A patient presenting with a red, swollen, painful joint poses a clinical challenge to both the emergency department doctor and to the General Practitioner, as the spectrum of severity is diverse, and there are several differential diagnoses. The reported annual incidence of septic (bacterial) arthritis in the native joint is estimated to be 2-60 per 100.000 individuals (1-3). This means that non-bacterial arthritis such as reactive arthritis (incidence 6-300 per 100.000) or gout (incidence 100-300 per 100.000) are more likely to be the final diagnosis (4, 5). Thus, in the emergency department, less than one in four patients presenting with a single acutely painful joint will have a septic arthritis (6, 7). However, as septic arthritis is a serious condition with reported mortality rates of 7-11% (3, 8-11), that may be encountered by several specialties (e.g. infectious diseases, emergency medicine, internal medicine, rheumatology, orthopedy, intensive care), the importance of rapid clinical evaluation, collection of relevant samples for microbiological analysis, and interpretation of laboratory analyses followed by adequate treatment must be emphasized. The aim of this workgroup report is to summarize the current literature and evidence on **available tools for the primary diagnostic work-up in suspected septic arthritis of the native joint.**

Summary of recommendations and level of evidence for each clinical dilemma

1. **<u>Are clinical parameters important in the evaluation of a patient with an inflamed painful joint?</u>**
   *No clinical parameters can exclude or confirm septic arthritis, even if a thorough patient history may contain important information **(BII)**. However, clinical parameters are critical for identifying the patient with concomitant sepsis or septic shock, requiring immediate attention and rapid adequate treatment **(BIII)**.*

   A thorough patient history may aid in the management of patients with suspected septic arthritis. Age (>80 years), diabetes, rheumatoid arthritis, concurrent superficial skin infection, iv drug use and previous arthrocentesis are risk factors for septic arthritis (1, 7, 12), while alcohol use, diet and medications may, together with chronic diseases and genetic factors, predispose for gout (4). A history of crystal-induced arthritis was associated to a lower likelihood of septic arthritis at an OR of 0.09 (95% CI 0.01-0.9) in a multivariate analysis (13). Furthermore, a history of bacterial gastroenteritis or genital infection with *Chlamydia trachomatis* may precede reactive arthritis (5).

   Septic arthritis presents as a monoarthritis in 85-94% of cases, and rheumatoid arthritis is a risk factor for septic polyarthritis (14-17).

   Fever has been described in 44-71% of patients (8, 13, 18), but was not significantly associated to septic arthritis (13) and was poorly sensitive in previous systematic reviews, at 57% (95%CI 52-62) and 34-54%, respectively (6, 7). Chills has been

described as significantly associated to septic arthritis at a frequency of 39.5% (13), but Margaretten *et al.* reported a sensitivity of 19% (95% CI 15-24) (7). In a prospective cohort (19) "Sepsis syndrome" (defined according to the now outdated sepsis-2 criteria as SIRS and infection) was present in 11.4% of patients and in a retrospective cohort (20), 7.6% of patients developed septic shock, emphasizing the importance of using a scoring system (e.g qSOFA or NEWS-2) in order to identify patients with concomitant sepsis (21).

2. **What is the indication for aspiration of joint fluid?**
When septic arthritis is suspected, aspiration of joint fluid should be performed as quickly as possible unless the risks of aspiration outweigh the benefits ***(AIII).***

As treatment of septic arthritis requires long-term pathogen-directed antibiotic treatment, often in combination with surgery, a correct diagnosis is essential. Analysis of joint fluid is crucial when differentiating septic arthritis from potential differential diagnoses, such as skin and soft tissue infections, septic bursitis, hemarthrosis, gout, pseudogout, reactive arthritis, rheumatoid arthritis or other arthropathies (7, 12, 22, 23).

3. **What are the risks of performing aspiration of joint fluid?**
In severely ill patients, the main risk associated to aspiration of joint fluid prior to starting antibiotic therapy is delay in treatment of concomitant sepsis and septic shock ***(BII)****. Arthrocentesis may be associated with the risk of an iatrogenic bacterial arthritis in an uninfected inflamed joint, while the risk for bleeding in patients treated with warfarin or DOACs is low* ***(BII).***

Performing joint aspiration must not delay adequate treatment of concomitant sepsis or septic shock (22, 24), as these are life-threatening events. Still, microbiological diagnosis and antimicrobial susceptibility testing are important to avoid suboptimal antibiotic treatment, and efforts should be made to secure cultures from blood and synovial fluid rapid enough not to delay treatment (22, 25).

In an Icelandic study, the risk of septic arthritis post-arthrocentesis was estimated to be low (0,037% per injection), but still constituted 17,9% of diagnosed cases of septic arthritis (12), while Kaandorp *et al.* described 1.6% of septic arthritis cases being preceded by arthrocentesis (15).

Arthrocentesis in patients with ongoing warfarin (26, 27) or direct oral anticoagulants (DOACs) (28) seem to be safe procedures.

4. **Which analyses should be made from synovial fluid?**
When analyzing synovial fluid in the setting of septic arthritis, several factors must be considered. The volume of aspirated synovial fluid may limit which and how many analyses are possible. Also, capacity and accessibility at the clinical laboratory may be another limitation ***(CIII)****.*

Bacterial cultures, synovial white blood cell count (synWBC) including neutrophil percentage (PMN%) and analysis for presence of crystals should be performed ***(AII)****. Additional analyses, such as Gram stain, synovial lactate or synovial glucose should be considered if possible* ***(BII).*** *Leucocyte esterase strip with or without glucose strip may add useful information bedside* ***(BIII)****. Molecular diagnostics such as PCR may be considered in select cases* ***(CIII).*** *The role of synovial calprotectin is yet to be determined* ***(CIII)****. Alpha-defensin is not recommended in this setting* ***(CIII)****.*

A recent review by Turner *et al.* (23) identified 15 studies evaluating diagnostic tests in septic arthritis.
As a positive synovial fluid culture was the most common reference standard for the diagnosis, this specific test was not evaluated. Bacterial growth in synovial fluid was part of Newman's criteria (29), and is a common reference standard for the diagnosis of septic arthritis in current publications (23). Optimizing microbiological methodology is important, as approximately 20% of synovial fluid cultures on solid media is reported to be false negative (6, 7, 30, 31). However, as bacterial growth from synovial fluid will also enable antibiotic susceptibility testing, cultures are crucial for both diagnosis and treatment of septic arthritis.

Synovial WBC count and synovial PMN percentage are widely used, even though several factors such as virulence of the pathogen, anatomical location of the joint and concomitant crystal arthropathy may influence the interpretation of the results (6, 7, 23, 32, 33). Further discussion on the use of synWBC/synPMN including bedside leucocyte esterase test can be found below ("Can certain levels of synovial leukocyte and/or differential count confirm/exclude septic arthritis?").

Detection of crystals in synovial fluid is important in differential diagnostics of the inflamed joint. However, the presence of crystals does not rule out a concomitant bacterial infection (23), as septic arthritis has been described in 1.5%-4.8% of patients with crystals in the synovial fluid (34, 35).

Lactate and glucose in synovial fluid are markers of bacterial metabolism, leading to elevated lactate and decreased glucose in septic arthritis. However, not all pathogens (e.g. *Neisseria gonorrhea*) lead to an increase in synovial lactate (36). Previous studies from the early 1980s using lactate cut-offs between 10-12 mmol/L showed sensitivity ranging from 0.86-1.0, and specificity 0.56-1.0 (22, 36). Lenski *et al.* compared synovial lactate ≥10 mmol/L in septic/gouty arthritis (37), while Shu *et al.* and Berthoud *et al.*, prospectively included patients with inflamed joints (36, 38).
A low (<1.0 mmol/L) synovial glucose has been described as a highly specific finding in septic arthritis (23). Furthermore, a lactate/glucose ratio ≥5 had a LR+ of 27 (36). This cohort was further analyzed, resulting in a proposed score (RESAS) composed by synWBC, presence of crystals, synovial lactate and synovial glucose. Also performing internal validation in an additional cohort, sensitivity ranged 0.56-0.92, specificity 0.98-0.98 giving a LR+ of 29.1-53.2 (39). The key findings regarding lactate and glucose are

summarized in Table 1.

Table 1. Summary of positive likelihood-ratios, sensitivity, and specificity (95% CI) for septic arthritis at different levels of synovial lactate (mmol/L) and glucose (mmol/L)

	Analysis	LR+	Sensitivity	Specificity
Berthoud *et al.* (36)	Lactate ≥11.0	74.9 (9.9-566)	0.36 (0.20-0.55)	0.99 (0.97-1.0)
	Glucose ≤1.0	33.3 (7.5-148)	0.32 (0.17-0.52)	0.99 (0.97-1.0)
	Lac/Gluc ratio ≥5	27.0 (9.5-76)	0.52 (0.34-0.70)	0.98 (0.95-0.99)
Lenski *et al.* (37)	Lactate ≥4.3	3.9 (1.8-8.6)	0.90 (0.76-0.96)	0.77 (0.57-0.90)
	Lactate>10	+∞	0.55	1.0
	Glucose ≤2.9	8.2	0.66 (0.51-0.78)	0.92 (0.75-0.98)
Shu *et al.* (38)	Lactate ≥5	2.3	0.55 (0.32-0.94)	0.76 (0.62-0.93)
	Lactate≥10	7.9	0.27 (0.1-0.72)	0.97 (0.79-0.99)
Omar *et al.* (40)	Glucose ≤1.4	12.5	1.0 (0.78-1.0)	0.92 (0.84-0.97)

<u>Broad-range 16s rDNA PCR</u> is appealing, especially in patients on antibiotic therapy at the time of arthrocentesis. Coiffier *et al.* found a sensitivity of 0.24 (95% CI 0.12-0.40) and specificity 1.0 (95%CI 0.94-1.0) in a cohort including 34 patients with septic arthritis. However, in three of those, PCR was positive while cultures were negative, suggesting a role for molecular diagnostics in select cases (41).

<u>Synovial calprotectin</u> is another biomarker available as point-of-care test for PJI, even though it is not yet part of diagnostic algorithms (42, 43). For native joint arthritis, synovial calprotectin has been evaluated in two prospective studies (44, 45). Couderc *et al.* presented sensitivity of 73% (95% CI 45%-92%), specificity 67% (95% CI 41%-87%) and LR+ 2.2 (95% CI 1.1-4.5) using the threshold 854 mg/L, while Baillet *et al.* found sensitivity 73%, specificity 94% and LR+ 11.6 using the threshold 150 mg/L. Lowering the cut-off to 52 mg/L, consistent with the threshold for one commercially available PJI point-of-care test, resulted in sensitivity 96%, specificity 44% and LR+ 1.7. However, this cut-off yielded a LR- of 0.09, thus making synovial calprotectin a possible test for ruling out septic arthritis (23). Still, the wide range of published thresholds raises questions about the clinical utility of synovial calprotectin in this setting (46). Further studies are needed to determine the usefulness of calprotectin in the diagnosis of septic arthritis in the native joint.

<u>The alpha-defensin</u> point-of-care lateral flow test kit has been developed and evaluated in prosthetic joint infections (PJI) (47), and is also included in two recent PJI definitions (42, 43). Cooper *et al.* (48) evaluated a retrospective cohort including 40 patients in whom alpha-defensin had been analyzed in synovial fluid aspirated from native knee joints. SynWBC and syn PMN percentage were significantly higher among alpha-defensin positive patients. Both culture-positive synovial fluids were also alpha-defensin positive, leading to a sensitivity of 100% (95% CI 16%-100%), specificity of 68% (95% CI 51%-83%), NPV 100% (95% CI 87%-100%) and PPV 14% (95% CIU 2%-43%). However, there was a high false-positive rate (28%), including a 64%

positivity in infection-free samples containing crystals. The authors recommend against the use of alpha-defensin in distinguishing between septic and crystalline arthritis.

5. **Which analysis are most important when the fluid volume is low?**
Bacterial cultures, synovial white blood cell count (synWBC) including neutrophil percentage (PMN%) and analysis for presence of crystals (in that order) are the most important analyses when fluid levels are low. ***(AII)****. Leucocyte esterase strip with or without glucose strip may add useful information if the remaining fluid volume after cultures is too small for standard clinical chemistry analysis* ***(BIII).***

6. **Can certain levels of synovial leukocyte and/or differential count confirm/exclude septic arthritis?**
A synovial WBC count (synWBC) of >50,000 cells/µL is suggestive of septic arthritis but is not alone sufficient for diagnosis. Only extreme values of synWBC (>100,000 cells/µL) can reliably diagnose septic arthritis ***(BII)****. Limited data suggests that these cutoffs may not be applicable in immunosuppressed patients* ***(CIII)****.*
A positive leukocyte esterase strip (++ or +++) is a valid marker for high synWBC and add valuable information, either bedside or if synovial fluid volume is low ***(BII).***
The existing literature on cell differentiation is not conclusive and the data should be cautiously interpreted. As such, it is not possible to give a recommendation for a particular percentage of synovial polymorphonuclear leukocytes (synPMN) to diagnose septic arthritis ***(BII)****. Low synWBC (<25,000 cells/µL) can decrease post-test probability, but given the current level of evidence it cannot exclude septic arthritis* ***(BII)****. There is not enough data to elaborate potential situations in which a normal synPMN count would exclude septic arthritis* ***(CIII)****.*

Leucocyte cell count (synWBC)
Septic arthritis is clinically difficult to distinguish from other inflammatory conditions in joints. As septic arthritis can rapidly damage the affected joint, and the optimal management of septic arthritis requires antibiotic therapy and orthopedic intervention, diagnostic tests that can quickly determine whether infection or sterile inflammation is the etiology behind the arthritis, are essential. Synovial leukocyte count is a readily available analysis and is often the first objective measurement available to the clinician.
In several studies, data are presented as sensitivity, specificity and likelihood ratios (LR) at a defined cutoff value. In general, a LR+ >10 indicates that a positive test may be useful in confirming a diagnosis, while LR- <0.1 indicates that a negative result may be useful in excluding it (23).

Confirming septic arthritis ("rule-in"):
Several reviews recommend starting empirical treatment for septic arthritis if the synovial leukocyte count is above 50,000 cells/µL (6, 7, 30) .
To date, only two meta-analysis have been performed (6, 7). However, both these studies are essentially based on the same original papers. The scope of the Carpenter *et al.* meta-analysis is on septic arthritis in the emergency department setting, even

though the authors do acknowledge that not all original papers included are solely on emergency department patients, hence the risk of spectrum bias. The Carpenter *et al.* meta-analysis is based on 7 published studies on septic arthritis, yet certain studies had to be excluded for the analysis of sensitivity (1 study) and specificity (3 studies) due to heterogeneity. As such, the final analysis yields comparable, but distinct findings compared to the work by Margaretten *et al.*

Table 2. Summary of positive likelihood-ratios, sensitivity, and specificity (95% CI) for septic arthritis at different levels of synovial WBC counts

	**synWBC (cells/μL)**	**LR+**	**Sensitivity**	**Specificity**
**Meta-analysis**				
Margaretten *et al.* (7)	<25,000	0.32 (0.23-0.43)		
	≥25,000	2.9 (2.5-3.4)		
	>50,000	7.7 (5.7-11.0)		
	>100,000	28 (12-66)		
Carpenter *et al.* (6)	>25,000	3.2 (2.3-4.4)		
	>50,000	4.7 (2.5-8.5)	0.56 (0.49-0.63)	0.90 (0.88-0.92)
	>100,000	13.2 (3.6-51.1)		
**Subsequent publications**				
Couderc *et al.* (13)	>10,000	1.36	0.89	0.35
	>50,000	3.14	0.57	0.82
	>100,000	3.93	0.29	0.93
Borzio *et al.* (49)	>64,000	4	0.40	0.90
Ferreyra et al. (50)	>50,000	5.8	0.54	0.92
	>70,000	8.9	0.39	0.96
Shu *et al.* (38)	>50,000	7.9	0.27 (0.1-0,71)	0.97 (0.79-0.99)
Berthoud et al. (36)	>50,000	3.6	0.72	0.80
Coiffier *et al.* (39)	≥70,000	4.51 (3.07-6.63)		

Since the publication of the most recent meta-analysis, several original papers have been published. Coiffier *et al*. (39) conducted a cross-sectional study based on a prospective cohort of unselected acute arthritis patients on native joints and found that purulent synovial fluid or synWBC ≥70,000 cells/µL had a LR+ 4.51 (95%CI 3.07 – 6.63) with a OR 17.9 (95%CI 6.18 – 51.6) in the univariate analysis vs. OR 13.8 (95%CI 3.39 – 56.0) in the multivariate analysis. Borzio *et al*. (49) conducted a retrospective study on 458 patients identified via inpatient databases from 2 hospitals for whom a complete dataset could be extracted, and found that a synWBC >64,000 cells/µL had a LR+ of 4 and a sensitivity of 40% and a specificity of 90%. Couderc *et al.* (13) conducted a prospective observational cohort study on consecutive patients referred to a rheumatologist in the setting of acute arthritis and found quite low LR+ even at high leukocyte counts. Ferreyra *et al.* studied a retrospective cohort consisting of acute and chronic arthritis, while Berthoud *et al.* performed a prospective study on arthritis with a duration <30 days (36, 50). Finally, Bell *et al.* published a small retrospective cohort (n=33) of patients under immunosuppression. Only 31% of patients with septic arthritis had a synWBC >50,000 cells/µL, and no useful threshold differentiating between infected and non-infected patients could be established. The key findings in these studies are summarized in Table 2.

Excluding septic arthritis ("rule-out"):
The meta-analysis by Carpenter *et al.* (6) found that synWBC of 0-25,000 cells/µL resulted in an LR of 0.33 (6). As such, only in situations with low pretest probability of septic arthritis can a cell count <25,000 cells/µL reliably guide the clinician ($P_{posttest} = P_{pretest} \times LR/(1 - P_{pretest} + P_{pretest} \times LR)$). For example, a synWBC of 20,000 cells/µL would decrease pretest probability of septic arthritis from 10% to approximately 3.8%. Several studies have demonstrated that large proportions of included patients have positive cultures from aspirated synovial fluid in the setting of cell counts below 50.000 cells/ µL. Li *et al.* (11) found that 36% of 73 positive joint samples came from patients with synWBC <50,000 cells/µL. Baran *et al.* (32) found that 27% of 44 positive joint samples came from patients with synWBC <50,000 cells/µL.

<u>Leucocyte esterase test from synovial fluid</u>
A recent prospective study by Kolbeck *et al.* (51) on 455 patients with arthritis found excellent performance of leukocyte esterase strips (Combur 7). A total of 293 patients were included in the final dataset of which 41 had septic arthritis. The combination of a positive leucocyte esterase strip (++ or +++) with a negative glucose reading had a sensitivity of 85% (95% CI 75–96%) and specificity of 100 % (95% CI 100–100%,). The patients diagnosed with septic arthritis based on leucocyte esterase strips had a mean synWBC of 81,266 (25,700–522,600) cells/µL with mean PMN percentage of 93 (95% CI 84-98%).
Knapper *et al.* (52) performed a prospective multicenter study on 80 patients, in which septic arthritis was present in 5 patients. Using a similar leucocyte esterase strip (Combur 9) with identical threshold for positivity (++ or +++), sensitivity was 100% (95% CI 47.8–100) and specificity was 30% (95% CI 20.5–42.4).

Cell differentiation (neutrophil count/percentage)

Confirming septic arthritis ("rule-in"):

Carpenter *et al.* found, based on 3 studies, that synovial polymorphonuclear leukocytes (PMNs) >90% had sensitivity of 60% and a specificity of 78% with a +LR of 2.7 (6) whereas Margaretten *et al.* found that synovial PMN >90% had a LR+ of 3.4, but this was based on no more than 4 studies (whereof only 1 study was used in both meta-analysis) in which differential counts was performed (7).

Subsequently a few original papers have addressed cell differentiation. Baran *et al.* (32) conducted a retrospective study to assess the sensitivity and specificity of synovial fluid for a positive culture of various parameters. For PMN differentiation they found decreasing sensitivity and increasing specificity as percentage of PMNs increased. The corresponding LR+ for 80, 85 and 90% are 2.0, 2.1 and 2.5 respectively when calculated (LR+ = sensitivity/1-specificity).
Couderc *et al.* found that synPMN >90% had a LR+ of 2.26 and a sensitivity of 42% and a specificity of 82% (13). Ferreyra *et al.* conducted a retrospective study based on two cohorts of acute or chronic mono- or polyarthritis (50) in which synovial fluid with complete cytologic profiles were analyzed. Amongst patients with septic arthritis, the synPMN percentage was 91.6 ± 7.7 (mean, SD), and synPMN >95% reached a LR+ of 4.55. Finally, Berthoud *et al.* (36) performed a prospective, single-center study on acute (<30 days) joint effusions. The key findings in these studies are summarized in Table 3.

Table 3. Summary of positive likelihood-ratios, sensitivity, and specificity for septic arthritis at different levels of synovial PMN percentages

	PMN (%)	LR+	Sensitivity	Specificity
**Meta-analysis**				
Margaretten *et al.* (7)	>90	3.4		
Carpenter *et al.* (6)	>90	2.7	0.6	0.78
**Subsequent publications**				
Baran *et al.* (32)	>80	2.0	0.93 (0.80-0.98)	0.54 (0.40-0.68)
	>85	2.1	0.89 (0.75-0.96)	0.58 (0.43-0.71)
	>90	2.5	0.82 (0.67-0.91)	0.67 (0.53-0.79)
Couderc *et al.* (13)	>90	2.26	0.42	0.82
Ferreyra *et al.* (50)	>90	3.52	0.71	0.79
	>95	4.55	0.50	0.89
Shu *et al.* (38)	>90	1.2	0.45 (0.24-0.87)	0.62 (0.47-0.82)
Berthoud *et al.* (36)	>90	1.7	0.59	0.64

Excluding septic arthritis ("rule-out"):

The authors have not been able to identify any study addressing this question

specifically. This question was not addressed in the work by Carpenter *et al.* or Margaretten *et al (6, 7),* and the studies by Li and Baran (11, 32) did not correlate synWBC with differential counts. However, Ferreyra *et al.* calculates a LR- 0.07 for synPMN <80% in a retrospective study based on two cohorts of acute or chronic mono- or polyarthritis (50).

7. **Do these cut-off levels apply to all joints?**
*It is currently unknown whether differential thresholds of synWBC or synPMN percentages should be used for different joints when diagnosing septic arthritis **(CIII)**.*

The available data on septic arthritis seldom contains details on the anatomical location of the affected joint. A single retrospective study on native knee joints found that a synWBC ≥30,000 cells/µL had a OR of 90.8 in the multivariable analysis (95% CI 26.6 – 310.1) (53). Ottink *et al.* states in a review (33) that the optimal thresholds for both synWBC and synPMN percentage are affected by location of affected joint in diagnosing prosthetic joint infection (PJI). Their analysis implies that the optimal synWBC cutoff would be highest in shoulder PJI, followed by hip PJI with the lowest cutoff in knee PJI. Whether the synWBC differ from one anatomical location to another also in native septic arthritis is currently unknown and as of now, data supporting differential thresholds for synWBC or synPMN percentage in native septic arthritis is lacking. Further studies in this field are warranted.

8. **What blood/serum tests should be performed in patients with suspected septic arthritis (septic arthritis)?**
*Routine blood tests have neither the sensitivity nor the specificity to verify or exclude septic arthritis **(BII)**. However, blood cultures may provide the microbiological diagnosis and CRP, PCT and WBC count (including neutrophil percentage) are routine investigations that may support the diagnostic hypothesis and should be performed on all patients suspected of having septic arthritis **(AII)**. CRP kinetics may be useful when monitoring clinical response to treatment **(BIII)**. Creatinine and liver ALTs are important for optimizing antibiotic doses in septic arthritis **(BIII)**.*

Blood cultures in patients with septic arthritis has been reported to be positive in 9-41% (10, 17, 20, 22, 54), providing microbiological diagnosis and antibiotic susceptibility patterns. Blood cultures can be taken at the same time as routine blood analyses, still they were only performed in 56-70% of septic arthritis cases (20, 54). A positive blood culture, especially if *S. aureus* or streptococci are found, is reason to perform further diagnostic tests to exclude septic foci, such as endocarditis (22).

ESR (erythrocyte sedimentation rate) levels has in several studies been reported to be higher in septic arthritis compared to non-infectious arthritis (10, 11, 13, 55, 56). Using differentiated cutoff values (men: 17 mm/h, women: 25 mm/h), Talebi-Taher *et al.* (56) calculated a sensitivity of 100%, albeit with a specificity of 26%. Several studies have reported decent sensitivity, but as the specificity for this analysis has overall been poor (6, 7) the benefit of ESR in this setting is limited.

CRP. In general, higher CRP values has been reported in patients with septic arthritis compared to non-infectious arthritis (10, 13, 19, 54, 55, 57). However, Chouk *et al.* found no significant difference in CRP values between septic arthritis and gout (16), and overall, the diagnostic power is limited due to low specificity (6, 7, 23). Even if CRP alone is insufficient to diagnose septic arthritis, it is a useful component when evaluating patients with an acute inflamed joint. Furthermore, as CRP is expected to decline during successful treatment, CRP kinetics is a useful tool to monitor response to treatment (25, 58)

PCT (procalcitonin). There are several studies (10, 16, 18, 19, 56, 57, 59) and meta-analyses (60-62) on the use of serum PCT in diagnosing septic arthritis. In general, serum PCT levels are higher in septic arthritis than in non-infectious arthritis. Chouk *et al.,* however, found no significant difference between serum PCT levels when comparing septic arthritis and gout (16). Concomitant infection at a distant site from inflammatory arthritis lowers the performance of serum PCT (18), and serum PCT levels were similar when comparing patients with chronic gouty arthritis complicated by fever caused by infection (all-cause) or non-infectious fever (63). Several different cut-offs have been suggested. Hugle *et al.* (18) proposed that septic arthritis is highly unlikely when serum PCT is below 0.1 ng/mL (sensitivity 100%, specificity 46%), and unlikely if below 0.25 ng/mL (LR- 0.09). In a recent review, Turner *et al.* calculated a negative likelihood ratio of <0.1 for PCT <0.39 ng/mL suggesting that this would be sufficient to consider as a “rule-out” test if synovial WBC count is < 50.000/µL (23). However, current French guidelines (22) recommends against the use of PCT, as a test result <0.5 mg/mL does not rule out septic arthritis.

Leucocyte count (WBC) in serum has too low specificity to be useful in distinguishing septic arthritis from non-infectious arthritis (6, 7). While a single study suggested significantly elevated WBC and neutrophil percentage in serum (19), several other studies found no difference whatsoever (11, 13, 55).

Other blood/serum tests

Routine blood tests evaluating kidney and liver function are essential to assess kinetics and metabolism of antimicrobial agents, in order to optimize dosing and minimize the risk of adverse effects.

Serological analyses are required in the diagnosis of certain pathogens in bacterial arthritis (e.g., borreliosis, brucellosis, chronic Q-fever), and should be considered in cases where these are suspected.

9. Does normal blood-CRP exclude septic arthritis?

There is not enough data to elaborate potential situations in which a normal blood-CRP would exclude septic arthritis ***(CIII).***

The authors have not been able to identify any study addressing this question specifically. Septic arthritis encompasses a range of pathogens of different virulence,

which may impact the clinical presentation. Furthermore, a short symptom duration may infer false low CRP values. As CRP starts to rise in 6 hours followed by a peak at 48 hours (64), the timeline of the current symptoms is crucial when evaluating a CRP value. Also, therapeutic immunomodulatory drugs may affect the production of inflammatory markers. Notably, the IL-6 inhibitor tociluzumab used in rheumatoid arthritis, shuts off IL-6-mediated production of acute phase proteins (e.g., CRP) from the liver which may mask even severe infections (65).

Setting the cutoff at CRP >15-18 mg/L yields a sensitivity of 92% (13, 56). Furthermore, Gupta *et al.* described elevated CRP values in 74 of 75 patients (8), and Bell *et al.* in 15 of 16 immunosuppressed patients with septic arthritis (66). Still, even if several studies indicate significantly higher CRP in septic arthritis than in non-infectious arthritis (10, 13, 19, 54, 56), data is lacking whether a normal blood CRP may exclude septic arthritis, neither in normal or immunosuppressed patients, nor for specific more virulent pathogens (e.g., *S. aureus*, hemolytic streptococci).

However, in a recent review (23), Turner *et al.* proposed considering one or more analyses with a calculated negative LR<0.1 (blood PCT <0.39 ng/mL, serum TNF-alpha <10 pg/μL, synovial neutrophil count <15,000 cells/μL, synovial neutrophils < 80%, synovial calprotectin <52 mg/L) as "rule-out" test in patients with less than 50,000 cells/μL WBCs in synovial fluid. This algorithm remains to be validated.

10. <u>What is the role of imaging in patients with suspected septic arthritis?</u>

*Systematic use of radiology is not indicated in septic arthritis, and radiology should not delay arthrocentesis and treatment unless necessary **(CIII)**. Radiographs are useful in diagnosing pre-existing conditions as well as be a reference for future monitoring and may detect changes consistent with septic arthritis **(BIII)**. Ultrasound may be useful to detect joint effusion, and in select cases may be used to guide a joint aspiration **(BIII)**. The usefulness of MRI is limited by accessibility, but MRI may be required for diagnosis in specific joints **(BIII)**.*

<u>Radiographs</u> are safe and accessible, and may both add information regarding pre-existing conditions as well as be a reference for future monitoring (67) Radiological findings, such as reduced joint space, cartilage destruction and later osseous erosions and osteomyelitis can evolve rapidly but may also not be visible on standard radiological imaging for ten days (22, 67). Still, they were significantly more common among patients with septic arthritis compared to inflammatory arthritis in a prospective cohort published by Couderc *et al.*, even though the sensitivity was 0.3, specificity 0.95 and LR+5.8 (13).

<u>Ultrasound.</u> Synovitis (96%) and joint effusion (93%) already at the start of antibiotic therapy were common findings using ultrasound (68). Furthermore, joint aspirations in suspected septic arthritis can be performed using ultrasound guidance (67).

<u>Magnetic resonance imaging (MRI)</u> is the radiologic modality of choice for detecting

concomitant osteomyelitis (67). The usefulness of MRI in septic arthritis has been evaluated (69, 70), and it may be particularly useful in certain locations, such as the sacroiliac joint (22, 71). However, limited access to MRI renders this modality inconvenient in the acute setting.

FDG-PET CT. As FDG accumulates in both septic and inflammatory arthritis, there is currently no evidence for using FDG-PET in the diagnosis of septic arthritis (67).

Disclosures

Staffan Tevell: none, Karianne Wiger Gammelsrud: none, Nis Pedersen Jørgensen: none

References

1. Kaandorp CJ, Van Schaardenburg D, Krijnen P, Habbema JD, van de Laar MA. Risk factors for septic arthritis in patients with joint disease. A prospective study. Arthritis Rheum. 1995;38(12):1819-25.
2. Long B, Koyfman A, Gottlieb M. Evaluation and Management of Septic Arthritis and its Mimics in the Emergency Department. West J Emerg Med. 2019;20(2):331-41.
3. Abram SGF, Alvand A, Judge A, Beard DJ, Price AJ. Mortality and adverse joint outcomes following septic arthritis of the native knee: a longitudinal cohort study of patients receiving arthroscopic washout. Lancet Infect Dis. 2020;20(3):341-9.
4. Singh JA, Gaffo A. Gout epidemiology and comorbidities. Semin Arthritis Rheum. 2020;50(3S):S11-S6.
5. Wendling D, Prati C, Chouk M, Verhoeven F. Reactive Arthritis: Treatment Challenges and Future Perspectives. Curr Rheumatol Rep. 2020;22(7):29.
6. Carpenter CR, Schuur JD, Everett WW, Pines JM. Evidence-based diagnostics: adult septic arthritis. Acad Emerg Med. 2011;18(8):781-96.
7. Margaretten ME, Kohlwes J, Moore D, Bent S. Does this adult patient have septic arthritis? Jama-Journal of the American Medical Association. 2007;297(13):1478-88.
8. Gupta MN, Sturrock RD, Field M. A prospective 2-year study of 75 patients with adult-onset septic arthritis. Rheumatology (Oxford). 2001;40(1):24-30.
9. Cooper C, Cawley MID. Bacterial Arthritis in an English Health District - a 10 Year Review. Annals of the Rheumatic Diseases. 1986;45(6):458-63.
10. Soderquist B, Jones I, Fredlund H, Vikerfors T. Bacterial or crystal-associated arthritis? Discriminating ability of serum inflammatory markers. Scandinavian Journal of Infectious Diseases. 1998;30(6):591-6.
11. Li SF, Henderson J, Dickman E, Darzynkiewicz R. Laboratory tests in adults with monoarticular arthritis: can they rule out a septic joint? Acad Emerg Med. 2004;11(3):276-80.

12. Geirsson AJ, Statkevicius S, Vikingsson A. Septic arthritis in Iceland 1990-2002: increasing incidence due to iatrogenic infections. Annals of the Rheumatic Diseases. 2008;67(5):638-43.
13. Couderc M, Pereira B, Mathieu S, Schmidt J, Lesens O, Bonnet R, et al. Predictive value of the usual clinical signs and laboratory tests in the diagnosis of septic arthritis. CJEM. 2015;17(4):403-10.
14. Dubost JJ, Fis I, Denis P, Lopitaux R, Soubrier M, Ristori JM, et al. Polyarticular septic arthritis. Medicine. 1993;72(5):296-310.
15. Kaandorp CJ, Dinant HJ, van de Laar MA, Moens HJ, Prins AP, Dijkmans BA. Incidence and sources of native and prosthetic joint infection: a community based prospective survey. Ann Rheum Dis. 1997;56(8):470-5.
16. Chouk M, Verhoeven F, Sondag M, Guillot X, Prati C, Wendling D. Value of serum procalcitonin for the diagnosis of bacterial septic arthritis in daily practice in rheumatology. Clinical Rheumatology. 2019;38(8):2265-73.
17. Weston VC, Jones AC, Bradbury N, Fawthrop F, Doherty M. Clinical features and outcome of septic arthritis in a single UK Health District 1982-1991. Ann Rheum Dis. 1999;58(4):214-9.
18. Hugle T, Schuetz P, Mueller B, Laifer G, Tyndall A, Regenass S, et al. Serum procalcitonin for discrimination between septic and non-septic arthritis. Clinical and Experimental Rheumatology. 2008;26(3):453-6.
19. Paosong S, Narongroeknawin P, Pakchotanon R, Asavatanabodee P, Chaiamnuay S. Serum procalcitonin as a diagnostic aid in patients with acute bacterial septic arthritis. Int J Rheum Dis. 2015;18(3):352-9.
20. Ruangpin C, Rodchuae M, Katchamart W. Factors Related to Surgical Treatment and Outcomes of Thai Patients With Septic Arthritis. J Clin Rheumatol. 2019;25(4):176-80.
21. Redfern OC, Smith GB, Prytherch DR, Meredith P, Inada-Kim M, Schmidt PE. A Comparison of the Quick Sequential (Sepsis-Related) Organ Failure Assessment Score and the National Early Warning Score in Non-ICU Patients With/Without Infection. Crit Care Med. 2018;46(12):1923-33.

22. Couderc M, Bart G, Coiffier G, Godot S, Seror R, Ziza JM, et al. 2020 French recommendations on the management of septic arthritis in an adult native joint. Joint Bone Spine. 2020;87(6):538-47.
23. Turner EHG, Lang MDH, Spiker AM. A narrative review of the last decade's literature on the diagnostic accuracy of septic arthritis of the native joint. J Exp Orthop. 2021;8(1):3.
24. Rhodes A, Evans LE, Alhazzani W, Levy MM, Antonelli M, Ferrer R, et al. Surviving Sepsis Campaign: International Guidelines for Management of Sepsis and Septic Shock: 2016. Intensive Care Med. 2017;43(3):304-77.
25. Elsissy JG, Liu JN, Wilton PJ, Nwachuku I, Gowd AK, Amin NH. Bacterial Septic Arthritis of the Adult Native Knee Joint: A Review. JBJS Rev. 2020;8(1):e0059.
26. Ahmed I, Gertner E. Safety of arthrocentesis and joint injection in patients receiving anticoagulation at therapeutic levels. Am J Med. 2012;125(3):265-9.
27. Thumboo J, O'Duffy JD. A prospective study of the safety of joint and soft tissue aspirations and injections in patients taking warfarin sodium. Arthritis Rheum. 1998;41(4):736-9.
28. Yui JC, Preskill C, Greenlund LS. Arthrocentesis and Joint Injection in Patients Receiving Direct Oral Anticoagulants. Mayo Clin Proc. 2017;92(8):1223-6.
29. Newman JH. Review of septic arthritis throughout the antibiotic era. Ann Rheum Dis. 1976;35(3):198-205.
30. Ross JJ. Septic Arthritis of Native Joints. Infect Dis Clin North Am. 2017;31(2):203-18.
31. Hughes JG, Vetter EA, Patel R, Schleck CD, Harmsen S, Turgeant LT, et al. Culture with BACTEC Peds Plus/F bottle compared with conventional methods for detection of bacteria in synovial fluid. J Clin Microbiol. 2001;39(12):4468-71.
32. Baran S, Price C, Hak DJ. Diagnosing joint infections: synovial fluid differential is more sensitive than white blood cell count. Eur J Orthop Surg Traumatol. 2014;24(8):1469-74.
33. Ottink KD, Strahm C, Muller-Kobold A, Sendi P, Wouthuyzen-Bakker M. Factors to Consider When Assessing the Diagnostic Accuracy of Synovial Leukocyte Count in Periprosthetic Joint Infection. J Bone Jt Infect. 2019;4(4):167-73.
34. Papanicolas LE, Hakendorf P, Gordon DL. Concomitant septic arthritis in crystal monoarthritis. J Rheumatol. 2012;39(1):157-60.

35. Shah K, Spear J, Nathanson LA, McCauley J, Edlow JA. Does the presence of crystal arthritis rule out septic arthritis? J Emerg Med. 2007;32(1):23-6.
36. Berthoud O, Coiffier G, Albert JD, Gougeon-Jolivet A, Goussault C, Bendavid C, et al. Performance of a new rapid diagnostic test the lactate/glucose ratio of synovial fluid for the diagnosis of septic arthritis. Joint Bone Spine. 2020;87(4):343-50.
37. Lenski M, Scherer MA. Analysis of synovial inflammatory markers to differ infectious from gouty arthritis. Clin Biochem. 2014;47(1-2):49-55.
38. Shu E, Farshidpour L, Young M, Darracq M, Ives Tallman C. Utility of point-of-care synovial lactate to identify septic arthritis in the emergency department. Am J Emerg Med. 2019;37(3):502-5.
39. Coiffier G, Berthoud O, Albert JD, Robin F, Goussault C, Gougeon-Jolivet A, et al. Elaboration of a new synovial predictive score of septic origin for acute arthritis on the native joint (RESAS). Rheumatology (Oxford). 2021;60(5):2238-45.
40. Omar M, Reichling M, Liodakis E, Ettinger M, Guenther D, Decker S, et al. Rapid exclusion of bacterial arthritis using a glucometer. Clin Rheumatol. 2017;36(3):591-8.
41. Coiffier G, David C, Gauthier P, Le Bars H, Guggenbuhl P, Jolivet-Gougeon A, et al. Broad-range 16 s rDNA PCR in synovial fluid does not improve the diagnostic performance of septic arthritis in native joints in adults: cross-sectional single-center study in 95 patients. Clin Rheumatol. 2019;38(7):1985-92.
42. Shohat N, Bauer T, Buttaro M, Budhiparama N, Cashman J, Della Valle CJ, et al. Hip and Knee Section, What is the Definition of a Periprosthetic Joint Infection (PJI) of the Knee and the Hip? Can the Same Criteria be Used for Both Joints?: Proceedings of International Consensus on Orthopedic Infections. J Arthroplasty. 2019;34(2S):S325-S7.
43. McNally M, Sousa R, Wouthuyzen-Bakker M, Chen AF, Soriano A, Vogely HC, et al. Infographic: The EBJIS definition of periprosthetic joint infection. Bone Joint J. 2021;103-B(1):16-7.
44. Couderc M, Peyrode C, Pereira B, Miot-Noirault E, Mathieu S, Soubrier M, et al. Comparison of several biomarkers (MMP-2, MMP-9, the MMP-9 inhibitor TIMP-1, CTX-II, calprotectin, and COMP) in the synovial fluid and serum of patients with and without septic arthritis. Joint Bone Spine. 2019;86(2):261-2.

45. Baillet A, Trocme C, Romand X, Nguyen CMV, Courtier A, Toussaint B, et al. Calprotectin discriminates septic arthritis from pseudogout and rheumatoid arthritis. Rheumatology (Oxford). 2019;58(9):1644-8.
46. Grzelecki D, Kowalczewski J. Comment on: "Comparison of several biomarkers (MMP-2, MMP-9, the MMP-9 inhibitor TIMP-1, CTX-II, calprotectin, and COMP) in the synovial fluid and serum of patients with and without septic arthritis". Joint Bone Spine. 2019; 86(2):261-262. Joint Bone Spine. 2020;87(5):517-8.
47. Abdel Karim M, Andrawis J, Bengoa F, Bracho C, Compagnoni R, Cross M, et al. Hip and Knee Section, Diagnosis, Algorithm: Proceedings of International Consensus on Orthopedic Infections. J Arthroplasty. 2019;34(2S):S339-S50.
48. Cooper KB, Siegel ER, Stambough JB, Bumpass DB, Mears SC. The Alpha-Defensin Prosthetic Joint Infection Test Has Poor Validity for Native Knee Joint Infection. J Arthroplasty. 2021.
49. Borzio R, Mulchandani N, Pivec R, Kapadia BH, Leven D, Harwin SF, et al. Predictors of Septic Arthritis in the Adult Population. Orthopedics. 2016;39(4):e657-63.
50. Ferreyra M, Coiffier G, Albert JD, David C, Perdriger A, Guggenbuhl P. Combining cytology and microcrystal detection in nonpurulent joint fluid benefits the diagnosis of septic arthritis. Joint Bone Spine. 2017;84(1):65-70.
51. Kolbeck L, Haertle M, Graulich T, Ettinger M, Suero EM, Krettek C, et al. Leukocyte Esterase and Glucose Reagent Test Can Rule in and Rule out Septic Arthritis. In Vivo. 2021;35(3):1625-32.
52. Knapper T, Murphy RJ, Rocos B, Fagg J, Murray N, Whitehouse MR. Utility of bedside leucocyte esterase testing to rule out septic arthritis. Emerg Med J. 2021.
53. Holzmeister A, Frazzetta J, Yuan FFN, Cherones A, Summers H, Cohen J, et al. Evaluation for septic arthritis of the native adult knee is aided by multivariable assessment. Am J Emerg Med. 2020.
54. Pavic K, Pandya J, Sebak S, Shetty A, Spencer D, Manolios N. Acute arthritis: predictive factors and current practice in the approach to diagnosis and management across two hospitals in Sydney. Intern Med J. 2018;48(9):1087-95.

55. Rasmussen L, Bell J, Kumar A, Heckman MG, Lesser E, Whalen J, et al. A Retrospective Review of Native Septic Arthritis in Patients: Can We Diagnose Based on Laboratory Values? Cureus. 2020;12(6):e8577.

56. Talebi-Taher M, Shirani F, Nikanjam N, Shekarabi M. Septic versus inflammatory arthritis: discriminating the ability of serum inflammatory markers. Rheumatology International. 2013;33(2):319-24.

57. Fottner A, Birkenmaier C, von Schulze Pellengahr C, Wegener B, Jansson V. Can serum procalcitonin help to differentiate between septic and nonseptic arthritis? Arthroscopy. 2008;24(2):229-33.

58. Sierra R. C-Reactive Protein and Procalcitonin as Markers of Infection, Inflammatory Response, and Sepsis. Clinical Pulmonary Medicine. 2007;14(3):127-39.

59. Maharajan K, Patro DK, Menon J, Hariharan AP, Parija SC, Poduval M, et al. Serum Procalcitonin is a sensitive and specific marker in the diagnosis of septic arthritis and acute osteomyelitis. Journal of Orthopaedic Surgery. 2013;8:19.

60. Shen CJ, Wu MS, Lin KH, Lin WL, Chen HC, Wu JY, et al. The use of procalcitonin in the diagnosis of bone and joint infection: a systemic review and meta-analysis. Eur J Clin Microbiol Infect Dis. 2013;32(6):807-14.

61. Shaikh MM, Hermans LE, van Laar JM. Is serum procalcitonin measurement a useful addition to a rheumatologist's repertoire? A review of its diagnostic role in systemic inflammatory diseases and joint infections. Rheumatology. 2015;54(2):231-40.

62. Zhao JY, Zhang SF, Zhang L, Dong XH, Li JH, Wang Y, et al. Serum procalcitonin levels as a diagnostic marker for septic arthritis: A meta-analysis. American Journal of Emergency Medicine. 2017;35(8):1166-71.

63. Zhang J, Zhao C, Wu T, Su J, Wu XD, Liu J, et al. Procalcitonin may not be a differential diagnostic marker for bacterial infection in febrile patients with chronic gouty arthritis. J Int Med Res. 2018;46(10):4197-206.

64. Hirschfield GM, Pepys MB. C-reactive protein and cardiovascular disease: new insights from an old molecule. QJM. 2003;96(11):793-807.

65. Ogata A, Kato Y, Higa S, Yoshizaki K. IL-6 inhibitor for the treatment of rheumatoid arthritis: A comprehensive review. Modern Rheumatology. 2019;29(2):258-67.

66. Bell J, Rasmussen L, Kumar A, Heckman MG, Lesser ER, Whalen J, et al. Septic Arthritis in Immunosuppressed Patients: Do Laboratory Values Help? J Am Acad Orthop Surg Glob Res Rev. 2020;4(3).
67. Expert Panel on Musculoskeletal I, Beaman FD, von Herrmann PF, Kransdorf MJ, Adler RS, Amini B, et al. ACR Appropriateness Criteria((R)) Suspected Osteomyelitis, Septic Arthritis, or Soft Tissue Infection (Excluding Spine and Diabetic Foot). J Am Coll Radiol. 2017;14(5S):S326-S37.
68. Gaigneux E, Cormier G, Varin S, Merot O, Maugars Y, Le Goff B. Ultrasound abnormalities in septic arthritis are associated with functional outcomes. Joint Bone Spine. 2017;84(5):599-604.
69. Bierry G, Huang AJ, Chang CY, Torriani M, Bredella MA. MRI findings of treated bacterial septic arthritis. Skeletal Radiol. 2012;41(12):1509-16.
70. Karchevsky M, Schweitzer ME, Morrison WB, Parellada JA. MRI findings of septic arthritis and associated osteomyelitis in adults. AJR Am J Roentgenol. 2004;182(1):119-22.
71. Hermet M, Minichiello E, Flipo RM, Dubost JJ, Allanore Y, Ziza JM, et al. Infectious sacroiliitis: a retrospective, multicentre study of 39 adults. BMC Infect Dis. 2012;12:305.
