# Supplementary material for: Guideline for management of septic arthritis in native joints (SANJO)
Source: J Bone Jt Infect. 2023 Jan 12;8(1):29–37. doi: 10.5194/jbji-8-29-2023 (PMC9901514; doi:10.5194/jbji-8-29-2023)
Supplement: The supplement related to this article is available online at: https://doi.org/10.5194/jbji-8-29-2023-supplement. [file jbji-8-29-supplement.zip › Workgroup report 3, initial surgical treatment of SANJO.pdf]

EBJIS guideline Workgroup 3: Initial surgical treatment

Authors:
Dirk Jan F. Moojen
Department of Orthopaedic and Trauma Surgery, JointResearch, OLVG Hospital, Amsterdam, The Netherlands

Marta Sabater-Martos
Department of Orthopaedic and Trauma Surgery, Hospital Clínic de Barcelona, Spain

Ulf-Joachim Gerlach
Department of Septic Traumatology And Orthopedic Surgery, BG-Klinikum Hamburg, Germany

Topic:
Principles and indication for initial surgical treatment of septic arthritis

Septic arthritis of a native joint is generally seen as an acute septic problem with potential devastating sequelae(1), that needs immediate surgical treatment. Although this is the general opinion on how this diagnosis should be treated, there is a lack of high quality evidence to back up these claims. There are only 2 retrospective cohort studies with observational data that compare medical (joint aspiration) with surgical (arthroscopy or arthrotomy) treatment(2,3). Both studies originate from Rheumatology and include a limited number of patients during a long inclusion period. Results show that comparable results with regards to full recovery of joint functionality can be obtained with either treatment. Based on this low-quality data both studies conclude that surgical treatment is not superior to medical treatment.
Despite the lack of prospective, randomized clinical trials, there are multiple studies that report good results of surgical treatment either arthroscopic or open; especially for larger joints like the knee, hip, shoulder and ankle(4–8).

With regard to the surgical treatment there are 2 important additional questions:

1. Should the initial surgery be closed (arthroscopic debridement) or open (arthrotomy)?
2. What is the optimal timing of surgery, acute (<12 hours) or subacute (12-48 hours)?

Question 1: Initial surgery closed (arthroscopic debridement) or open (arthrotomy)?

Results: Literature search showed 1 small prospective RCT on this topic(9). A total of 21 patients with septic arthritis of the knee were randomized to debridement by either arthroscopy (n=10) or arthrotomy (n=11). In all patients, infection was successfully eradicated; 2 patients in the arthrotomy group needed a second debridement. The arthroscopy patients had a faster functional recovery. All patients had a Gächter stage I-III septic arthritis of the knee.
Four retrospective, comparative studies support the efficacy and advantages in functional recovery for arthroscopy.
Wirtz et al.,(10) reported on 51 patients with septic arthritis of the knee, of which 27 were treated arthroscopically and 24 by arthrotomy. They showed that when surgical treatment is initiated early (<5 days) and there is no osseous involvement arthroscopy leads to an effective eradication of infection and better functional results.
Böhler et al.,(11) reported on 70 patients with septic arthritis of the knee, of which 41 were treated arthroscopically and 29 by arthrotomy. The need for repeat surgery was significantly lower in the arthroscopy group (4.9 vs 20.7%, p=0.041) and range of motion was better as well (p<0.0001).
Johns et al.,(12) reported on 161 patients (166 joints) with septic arthritis of the knee, of which 123 were treated arthroscopically and 43 by arthrotomy. In this study 50 vs. 71% of patients had repeat surgery, respectively. The infection eradication rate was higher (p=0.011), the number of surgeries was lower (p=0.01) and the postoperative range of motion was higher (p=0.016) in the arthroscopy group.
Faour et al.,(13) reported on 695 patients with septic arthritis of the knee, of which 464 were treated arthroscopically and 231 by arthrotomy. There was no difference in infectious complications, but overall adverse events were significantly higher in the arthrotomy group (49 vs. 34%, p=0.0002).
No comparative studies were found on other joints than the knee. Even though all 5 studies support the advantages of using arthroscopy, there are factors that may modify this choice.

Stutz et al.,(14) reported on the arthroscopic treatment of septic arthritis of several different joints in 78 patients and graded them according to the Gächter classification. Patients with stage I needed repeat debridement in 5%, stage II in 52% and stage III in 75%. In 4% of patients this was done by open debridement. There were no patients with stage IV. The overall cure rate was 91%, but this was negatively influenced by the initial Gächter classification, with 96% and 95% success for stage I and II, but only 67% for stage III. They concluded that initial staging has important prognostic and therapeutic consequences.
These observations were confirmed in a study of 40 patients with septic arthritis of the knee by Balabaud et al(15). Patients were surgically treated with either arthroscopy (n=20), open debridement (n=6) or synovectomy (n=14). The Gächter stage was I in 8, II in 18, III in 11 and IV in 3. The found the delay between the onset of symptoms and surgical treatment was the major prognostic factor for success (p=0.023). Short time delay was defined as an average of 12 days and long as 23 days; no specific cut-off could be determined. This delay was significantly correlated with the Gächter staging (p=0.001). They concluded that arthroscopic debridement should be routine treatment, but that synovectomy

should be considered as the primary procedure as well in Gächter stage III or IV septic arthritis or when more conservative treatment doesn't lead to a fast improvement.
A third publication by Aïm et al.,(4) on 46 patients was in line with the 2 previous publications and identified Gächter stage III and IV as risk factor for failure in arthroscopic treatment.
As an alternative to either arthroscopic or open debridement only, there are also several case series that show favorable results of a 2-stage approach with initial open debridement, joint resection and spacer placement, and subsequent insertion of a total hip or knee arthroplasty in a selective group of patients with a more advanced stage septic arthritis and end-stage osteoarthritis (16–18).

Questions 2: Optimal timing of surgery, acute (<12 hours) or subacute (12-48 hours)?

Results: Literature search did not show any prospective study related to this question. Two retrospective studies did look specifically into this.
Kodumuri et al.,(19) reported on the effect of timing of arthroscopic debridement in 82 patients with septic arthritis of different joints (including 35% of patients with a joint arthroplasty). They stratified patients in 4 groups (<6, 6-12, 12-24 or 24-48 hours) according to the time delay between setting of the diagnosis and actual surgical treatment. They found no significant correlation between the time delay and mortality, ICU admissions or number of wash outs needed to control the infection.
A larger study by Lauper et al.,(20) investigated 204 episodes of septic arthritis of different native joints and also did not find a significant negative effect of the delay between diagnosis and surgical treatment using the same cut-offs. They concluded that for native septic arthritis, in the absence of clinical sepsis, immediate joint drainage does not appear to reduce the risk of sequelae compared with delayed drainage.
A third study by Dave et al.,(21) did find a significant relation (p=0.012) between time from presentation to surgery and the need for multiple procedures. They concluded that with arthroscopic irrigation and debridement, most patients with septic knee arthritis require only 1 surgical procedure to eradicate infection, but the need for multiple procedures increases with time from onset of symptoms to surgery.
The main focus of the 3 studies mentioned was control of infection. No clinical studies were found that investigated the long term effects of timing of surgery on joint degeneration.

Recommendations based on question 1 & 2:

Even though (serial) joint aspiration seems to have a role as well, we recommend surgical debridement for septic arthritis, especially in larger joints. Based on the limited evidence in the studies available, it appears that an arthroscopic debridement is an adequate option as initial surgical management in patients with Gächter stage I, II and probably also III septic arthritis. In patients with Gächter stage III and definitely IV an open debridement can be considered, since success rates of arthroscopic treatment rapidly decline in these stages. In patients with concomitant end stage arthritis a 2-stage protocol with interval spacer and subsequent joint arthroplasty can be considered as well.
Even though surgery for septic arthritis is an indication for acute treatment, there is limited evidence that 24-48 hours delay(in the non-septic patient) can be accepted without negative effects on infection control. It must be noted that there is some evidence that longer delays increase the need for a repeat debridement and that none of the studies looked at long term effects on joint degeneration.

References:

1. Abram SGF, Alvand A, Judge A, Beard DJ, Price AJ. Mortality and adverse joint outcomes following septic arthritis of the native knee: a longitudinal cohort study of patients receiving arthroscopic washout. The Lancet Infectious Diseases. 2020 Mar 1;20(3):341–9.
2. Ravindran V, Logan I, Bourke BE. Medical vs surgical treatment for the native joint in septic arthritis: a 6-year, single UK academic centre experience. Rheumatology (Oxford, England). 2009;48(10):1320–2.
3. Flores-Robles BJ, Jiménez Palop M, Sanabria Sanchinel AA, Andrus RF, Royuela Vicente A, Sanz Pérez MI, et al. Medical Versus Surgical Approach to Initial Treatment in Septic Arthritis: A Single Spanish Center's 8-Year Experience. Journal of Clinical Rheumatology. 2019 Jan 1;25(1):4–8.
4. Aïm F, Delambre J, Bauer T, Hardy P. Efficacy of arthroscopic treatment for resolving infection in septic arthritis of native joints. Orthopaedics and Traumatology: Surgery and Research. 2015 Feb 1;101(1):61–4.

5. de Sa D, Cargnelli S, Catapano M, Peterson D, Simunovic N, Larson CM, et al. Efficacy of Hip Arthroscopy for the Management of Septic Arthritis: A Systematic Review. Vol. 31, Arthroscopy - Journal of Arthroscopic and Related Surgery. W.B. Saunders; 2015. p. 1358–70.
6. Memon M, Kay J, Ginsberg L, de Sa D, Simunovic N, Samuelsson K, et al. Arthroscopic Management of Septic Arthritis of the Native Shoulder: A Systematic Review. Vol. 34, Arthroscopy - Journal of Arthroscopic and Related Surgery. W.B. Saunders; 2018. p. 625-646.e1.
7. Davis CM, Zamora RA. Surgical Options and Approaches for Septic Arthritis of the Native Hip and Knee Joint. Journal of Arthroplasty. 2020 Mar 1;35(3):S14–8.
8. Elsissy JG, Liu JN, Wilton PJ, Nwachuku I, Gowd AK, Amin NH. Bacterial Septic Arthritis of the Adult Native Knee Joint: A Review. JBJS reviews. 2020 Jan 1;8(1):e0059.
9. Peres LR, Marchitto RO, Pereira GS, Yoshino FS, de Castro Fernandes M, Matsumoto MH. Arthrotomy versus arthroscopy in the treatment of septic arthritis of the knee in adults: a randomized clinical trial. Knee Surgery, Sports Traumatology, Arthroscopy. 2016 Oct 1;24(10):3155–62.
10. Wirtz DC, Marth M, Miltner O, Schneider U, Zilkens KW. Septic arthritis of the knee in adults: Treatment by arthroscopy or arthrotomy. International Orthopaedics. 2001;25(4):239–41.
11. Böhler C, Dragana M, Puchner S, Windhager R, Holinka J. Treatment of septic arthritis of the knee: a comparison between arthroscopy and arthrotomy. Knee Surgery, Sports Traumatology, Arthroscopy. 2016;24(10):3147–54.
12. Johns BP, Loewenthal MR, Dewar DC. Open compared with arthroscopic treatment of acute septic arthritis of the native knee. Journal of Bone and Joint Surgery - American Volume. 2017;99(6):499–505.
13. Faour M, Sultan AA, George J, Samuel LT, Curtis GL, Molloy R, et al. Arthroscopic irrigation and debridement is associated with favourable short-term outcomes vs. open management: an ACS-NSQIP database analysis. Knee Surgery, Sports Traumatology, Arthroscopy. 2019 Oct 1;27(10):3304–10.
14. Stutz G, Kuster MS, Kleinstück F, Gächter A. Arthroscopic management of septic arthritis: Stages of infection and results. Knee Surgery, Sports Traumatology, Arthroscopy. 2000;8(5):270–4.
15. Balabaud L, Gaudias J, Boeri C, Jenny JY, Kehr P. Results of treatment of septic knee arthritis: A retrospective series of 40 cases. Knee Surgery, Sports Traumatology, Arthroscopy. 2007;15(4):387–92.
16. Fleck EE, Spangehl MJ, Rapuri VR, Beauchamp CP. An articulating antibiotic spacer controls infection and improves pain and function in a degenerative septic hip. In: Clinical Orthopaedics and Related Research. Springer New York LLC; 2011. p. 3055–64.
17. Shaikh AA, Ha CW, Park YG, Park YB. Two-stage approach to primary TKA in infected arthritic knees using intraoperatively molded articulating cement spacers. Clinical Orthopaedics and Related Research. 2014;472(7):2201–7.
18. Hochreiter B, Strahm C, Behrend H. Short-interval two-stage approach to primary total knee arthroplasty for acutely septic osteoarthritic knees. Knee Surgery, Sports Traumatology, Arthroscopy. 2016 Oct 1;24(10):3115–21.
19. Kodumuri P, Geutjens G, Kerr HL. Time delay between diagnosis and arthroscopic lavage in septic arthritis. Does it matter. International Orthopaedics. 2012 Aug;36(8):1727–31.
20. Lauper N, Davat M, Gjika E, Müller C, Belaieff W, Pittet D, et al. Native septic arthritis is not an immediate surgical emergency. Journal of Infection. 2018 Jul 1;77(1):47–53.
21. Dave OH, Patel KA, Andersen CR, Carmichael KD. Surgical Procedures Needed to Eradicate Infection in Knee Septic Arthritis. Orthopedics [Internet]. 2016;39(1):50–4. Available from: http://www.healio.com/doiresolver?doi=10.3928/01477447-20151222-05

**Study**	**year**	**Type of study**	**Evidence quality**
Peres LR	2016	RCT	⊕⊕⊕⊖<br>Moderate<br>No blinding, small size
Wirtz DC	2001	Case-control	⊕⊕⊖⊖<br>Low
Böhler C	2016	Case-control	⊕⊕⊖⊖<br>Low

Johns BP	2017	Cohort	⊕⊕⊖⊖<br>Low<br>They use OR in a cohort study, RR would be much more appropriate
Faour M	2019	Case- Control	⊕⊕⊕⊖<br>Moderate<br>Though is an observational study sample size being large and the effect size is important.
Stutz G	2000	Case series	⊕⊖⊖⊖<br>Very low
Balabaud L	2007	Consecutive series. Case-control	⊕⊕⊖⊖<br>Low
Aim F	2015	Observational	⊕⊖⊖⊖<br>Very low
Felck E	2011	Case series	⊕⊖⊖⊖<br>Very low
Shaikh AA	2014	Case series	⊕⊖⊖⊖<br>Very low
Hochreiter B	2016	Case series	⊕⊖⊖⊖<br>Very low
Kodumuri P	2012	Retrospective Cohort	⊕⊕⊖⊖<br>Low
Lauper N	2018	Retrospective Cohort	⊕⊕⊕⊖<br>Moderate<br>Though is a retrospective study sample size being large and the effect size is important.
Dave OH	2016	Retrospective cohort	⊕⊕⊖⊖<br>Low
