# Supplementary material for: Guideline for management of septic arthritis in native joints (SANJO)
Source: J Bone Jt Infect. 2023 Jan 12;8(1):29–37. doi: 10.5194/jbji-8-29-2023 (PMC9901514; doi:10.5194/jbji-8-29-2023)
Supplement: The supplement related to this article is available online at: https://doi.org/10.5194/jbji-8-29-2023-supplement. [file jbji-8-29-supplement.zip › Workgroup report 2, microbiological methods of SANJO.pdf]

EBJIS guideline Workgroup 2: Microbiological methods

Authors

Yvonne Achermann (University Hospital Zurich, University of Zurich, Switzerland)

Liselotte Coorevits (Ghent University Hospital, Belgium, AZ Groeninge, Belgium)

Parham Sendi (University Hospital of Basel, University of Basel AND Institute for Infectious Diseases, University of Bern, Bern, Switzerland).

Background and aim of clinical application

Numerous pathogens can cause native septic arthritis. Native arthritis can be classified according to the clinical presentation (acute versus chronic), according to mode of acquisition (community-acquired *versus* hospital acquired infection), or according to pathogenesis (hematogenous seeding of microorganisms from a distant infection focus *versus* direct inoculation either from of an adjacent infection or iatrogenous). The incidence of native septic arthritis in adults is approximately 10 cases per 100’000 inhabitants per year [1-3]. As delayed management of joint sepsis can lead to irreversible damage and disability, timely diagnosis and treatment is critical. The aim of this workgroup report is to summarize the current literature and evidence on **microbiological diagnostic methods and their challenges.**

Summary of recommendations and level of evidence for each clinical dilemma

1. **Which pathogens are the most common isolates pathogens?**

Septic arthritis is typically caused by a single microorganism [4]. The most common causing pathogens are staphylococci (*Staphylococcus aureus, Staphylococcus lugdunensis*, other coagulase-negative staphylococci) and streptococci [5, 6]. In older and immunocompromised patients, Gram-negative pathogens such as *Pseudomonas aeruginosa* [7] are also described. In patients after trauma or intravenous drug use, anaerobes and *Candida* spp. should be considered as the causing pathogen. Rare pathogens in septic arthritis are *Borrelia burgdorferi*, *Brucella* spp., *Coxiella burnetti* or *Mycobacterium tuberculosis* complex (see Table below). *Tropheryma whipplei* causes arthralgia in classic Whipple Disease.

In immunosuppressed patients, physicians should think of Mollicutes (*Ureaplasma/Mycoplasma*) [8, 9], fungi (*Candida* spp. *Scedosporium spp.*, *Phaeoacremonium* spp.) [10], *Mycobacterium tuberculosis* complex and non-tuberculous mycobacteria (e.g. *Mycobacterium chimaera, Mycobacterium marinum, Mycobacterium abscessus*).

Viruses (Parvovirus B19, Rubella, Hepatitis A, B, C, HIV, Chikungunya, Adenovirus, Coxsackievirus, Echovirus and others) can cause arthritis. However, they play a minor role and the clinical entity is not considered as pyogenic arthritis.

In the following table, clinical or epidemiological associations of rare pathogens are described.

Table. Pathogens and their diagnostic, and associations.

Pathogen	Diagnostic	Associations
Borrelia burgdorferi [11-13]	Serology<br>Specific PCR of synovial samples (Cultures only in reference centers)	Previous non- treated erythema migrans, recurrent attacks or persisting objective joint<br>swelling in one or a few large joints.
Neisseria gonorrhoeae [14]	Blood cultures, PCR for *N. gonorrhoeae* in urogenital, first-void urine or synovial samples.<br>Cultures with selected media only in selected cases for antimicrobial susceptibility testing.	In sexually active persons, often the only sign, or with fever, (pustular) skin lesions, polyarthralgia
Brucella spp. [15]	Serology<br>Culture (under BSL-3 conditions), Specific PCR of blood or synovial samples	Ingestion of unpasteurized dairy products, travel history or endemic area with animal contact
Coxiella burnetti (chronic Q-Fever)	Serology, Specific PCR of blood or synovial samples	Contact with sheep/goat, inhalation with contaminated aerosols. Previous hepatitis or pneumonia
Mycobacterium tuberculosis complex [16]	Acid-fast stain, culture, Specific PCR of synovial fluid of synovial biopsy, or histopathology of synovial biopsy	Epidemiological exposure<br>Living or having previously lived or traveled to endemic countries
Whipple disease (*Tropheryma whipplei*) [17]	PAS staining and specific PCR of synovial fluid or duodenal biopsy	Migratory arthralgia of the large joints, accompanying myalgia gastrointestinal symptoms, weight loss, fever, lymphadenopathy

BSL-3 = Biosafety level 3; PAS=periodic acid–Schiff

Category/Grade: Recommendation A, Evidence II

2. Which microbiological sample gives the best culture yield?

In order to estimate the possibility of septic arthritis, it is crucial to first investigate synovial leukocytes in addition to microbiological cultures first. Crystals in synovial fluid have to be searched to diagnose a crystal deposition disease, but cannot exclude septic arthritis.

The gold standard for diagnosing septic arthritis is the detection of the causative pathogen in synovial fluid or in synovial biopsies [4]. If skin commensals such as coagulase-negative staphylococci or *Cutibacterium* spp. are detected, the presence of phenotypically identical organisms in at least two different samples can help to distinguish contaminants from causative pathogens. If cultures are negative, other diagnostic methods (see question 4) in synovial samples should be considered to identify the causative pathogen.

Category/Grade: Recommendation A, Evidence II

In addition, two or more sets of blood cultures should be obtained in febrile patients [18, 19]. In 36% up to 78% of patients with septic arthritis, blood cultures are positive [5, 6]. In one study, blood cultures were positive in 14% of patients with negative synovial fluid cultures [4] , and hence had an added value in diagnosing septic arthritis.

Category/Grade: Recommendation A, evidence III

The diagnostic sensitivity of synovial biopsy samples is typically higher than synovial fluid. However, in non-septic cases, the analytical material primarily derives from a joint puncture (i.e.; synovial fluid). If synovial fluid analysis shows no microbiological growth despite elevated leucocytes (*see previous chapter about non-culture based methods*), a synovial biopsy may be required for stain, culture and histopathological evaluation to diagnose synovitis caused by slow growing pathogens as *Borrelia* spp., mycobacteria or fungi.

Category/Grade: Recommendation B, Evidence III

3. **What microbiological samples should be obtained in patients with suspected septic arthritis?**

Subsequent to the aforementioned paragraph in response to question number 2, we recommend to obtain the following samples:

- Synovial fluid for microbiological culture, leucocytes and crystals
- Synovial biopsies (in case of surgical intervention) for microbiological cultures and histopathological analysis.
- Synovial fluid for molecular analysis in patients taking antibiotics at the time of puncture or in patients with suspected difficult to cultivate pathogens
- Set of blood cultures (aerobic and anaerobic bottle) in febrile patients

Special diagnostic tests – see Table in question 1 – to exclude rare pathogens are not indicated in routine diagnostics

Category/Grade: Recommendation A, Evidence II-III

4. **What is the role of Multiplex PCR and Metagenomic analysis in diagnostic samples**

Multiplex PCR

Morgenstern, et al showed results of a prospective study investigating the role of the multiplex PCR Unyvero implant and tissue infection (ITI) assay in 22 patients with septic arthritis [20]. This PCR is a fully automated multiplex PCR aiming to cover over 100 targets, including both pathogens and antibiotic resistance genes. Sensitivity for PCR was only 23% with a high specificity of 91%. Although the sample size in this study was small, it revealed that the multiplex PCR Unyvero ITI i60 does not improve sensitivity compared to conventional cultures [21]. Currently, there are no convincing results from other automated multiplex PCRs [22].Therefore we not recommend to use Multiplex PCR as a routine diagnostic.

Category/Grade: Recommendation A, Evidence II-III

Metagenomics

Clinical metagenomics potentially detect all causing pathogens in a clinical sample as showed in a proof of concept study by Ruppé et al., investigating 24 cases of bone and joint infections [23]. However, this diagnostic method is not yet part of the routine microbiological routine and therefore not recommended.

Category/Grade: Recommendation A, Evidence III

5. What is the value of Gram stain of synovial fluid?

The sensitivity of Gram staining on synovial fluid is limited, often below 50% [24]. In a narrative review, the sensitivity, specificity, positive and negative predictive value of Gram stain has been summarized and showed 37%, 99%, 99%, and 28%, respectively with positive cultures as the gold standard [25]. Given its excellent specificity, however, Gram staining can provide early proof of infection, awaiting bacterial culture and/or PCR.

Category/Grade: Recommendation A, Evidence II-III

6. Which pre-analytical steps should be considered?

Specific pre-analytical steps are subject to inter-institutional differences. We strongly advice to establish a standard operating procedure (SOP) for these pre-analytical steps, based on the available infrastructure, material and devices, and expertise. The generation, implementation and validation of these SOPs requires a close collaboration between surgeons, infectious diseases specialists and clinical microbiologists.

Synovial fluid (preferably ≥1 mL) should always be sent for culture in a **sterile native** tube (i.e.; inoculating agar plates and broth with synovial fluid). If enough synovial fluid is obtained, the material can be inoculated in enrichment media such as blood culture bottle or thioglycolate broth. When joint fluid is inoculated in blood culture bottles, the maximal volumes recommended by the bottle manufacturer should be respected. Generally, up to 10 mL is the maximum volume for each bottle. Since blood cultures have included neutralizing substances, inoculation can be beneficial when antibiotics were already given. However, it can also emerge the risk of

cultivating contaminants such as coagulase-negative staphylococci or *Cutibacterium* spp. Therefore, standard culture on agar plates and broth should always be included.

Is it not beneficial to inoculate synovial fluid in tubes containing anticoagulants such as heparin, SPS (sodium polyanethole sulfonate), EDTA (ethylenediamine tetraacetic acid) or citrate, because some of them can inhibit bacterial growth (EDTA) or inhibit PCR testing (heparin).

Category/Grade: Recommendation A, Evidence III

7. **How to enhance the culture yield when samples were obtained during antibiotic treatment?**

Collection of synovial fluid and at least 2 sets of blood cultures should be performed prior to administration of antibiotics whenever possible. Culturing specimens collected during antibiotic treatment could potentially lead to false negative results [4]. Molecular methods (broad-spectrum, multiplex, or specific PCRs) should be considered in case of persistent suspicion of septic arthritis, despite negative culture results.

Category/Grade: Recommendation A, Evidence III

8. **Can samples be kept in a fridge until the lab is available and how urgent is it to plate the sample in the microbiology laboratory?**

Accurate laboratory diagnosis depends highly on the quality of specimens received. Proper sample management includes samples to arrive at the laboratory and being processed as soon as possible after collection [19]. Tissues, fluids, aspirates, biopsies and blood culture bottles should be transported at room temperature (in particular important for *Neisseria* spp.) and should not be refrigerated prior to processing or incubation. In case of delay, samples could be stored at room temperature up to 24 hours even when cultures and molecular tests are planned.

If samples were only taken for molecular testing, they should be stored at lower temperature (at least refrigerated, the lower the temperature, the better) awaiting analysis [18, 26].

Delayed processing (>30 minutes to 24 hours) of small volumes of fluid (<1 ml) compromises the recovery of bacteria that are sensitive to ambient conditions, e.g. *Neisseria gonorrhoeae* and *Streptococcus pneumoniae* [18, 26].

Category/Grade: Recommendation A, Evidence III

9. **Which analytical steps should be considered?**

Synovial fluid should always be sent for culture as native fluid and, if enough fluid was obtained, in blood culture bottles. Synovial fluid in native tubes should then be plated on agar plates and enrichment broth (thioglycolate) as an alternative or in addition to inoculation in blood culture bottles. Cohen et al. showed a sensitivity of 76 % using blood culture bottles (Bactec®) and

62% using agar plates in a large cohort of 5’000 synovial fluid samples [27]. Incubation time is recommended between 5 and 7 days according to local practice.

Enrichment cultures in blood cultures must not be performed without direct examination of the fluid. No PCR test can directly be performed from fluids aspirated from blood culture bottles.

In case tissue biopsies are obtained, they should be vortexed (e.g. with glass beads), homogenized and incubated on agar plates and for enrichment either in thioglycolate broth or in blood culture bottles. There are different protocols regarding agar plates in each laboratory to detect the most common Gram-positive and Gram-negative microorganisms. For detection of anaerobes, specific anaerobe agar plates should be used.

All samples should be labeled in a way that provides information on the exact localization or presence of pus.

Category/Grade: Recommendation A, Evidence III

10. When should culture be prolonged or supplemented by other diagnostic tests? 16S PCR?

In all cases with elevated leucocytes in synovia and/or high suspicious of infection but negative cultures after 7 days, cultivation should be considered to be prolonged for up to 10 to 14 days. Additional cultures for fungal and mycobacteria, PCR or serology should be initiated (see chapter “**uncommon microbiology**”).

In addition, nucleic acid amplification tests (e.g.; 16s rDNA PCR, specific PCR) may be useful in patients with a certain clinical context (see Table 1) and/or in case of negative culture results due to administration of antibiotics prior to sampling. The cultures could be also falsely negative when microorganisms are present in low amount, fastidious or non-cultivable. As molecular tests are highly susceptible to contamination (extremely small amounts of contaminating material can lead to false positive results), results should always be critically reviewed by an Infectious Diseases specialist or a Clinical Microbiologist. One may use a broad-range molecular testing, detecting the 16S rRNA gene (conserved bacterial sequence) followed by sequencing for identification, a multiplex PCR targeting a limited panel of pathogens or a targeted PCR (i.e. *Neisseria gonorrhoeae*) [18].

Category/Grade: Recommendation A, Evidence III

11. When should synovial aspiration be repeated?

A repeat aspiration may be useful in patients with discrepancy between the probability of infection and the initial aspiration culture result. The value of a repeated aspiration should be weighed against the clinical course between the first aspiration and the intended second puncture.

Category/Grade: Recommendation A, Evidence III

12. Which techniques are recommended for joint aspiration? Principles for joint aspiration – e.g. ballooning soft spots / avoid cellulitis?

A key principle of adequate specimen collection is to avoid contamination with commensal microbiota surrounding the site of infection, as this could potentially lead to misleading results. Besides, non-sterile aspiration could lead to infection of the patient. The needle puncture site needs to be disinfect with 70% alcohol and disinfected with an iodine solution (1-2% tincture of iodine of 10% solution of povidone-iodine) or chlorhexidine 2% alcohol. The skin should be completely dry before inserting the needle [28, 29].

When aspirating a red, swollen joint with possible overlying cellulitis, there is a theoretical risk of inoculating the sterile joint with bacteria, subsequently leading to septic arthritis. However, as clinical evidence on this occurrence lacks [30], this finding may not stand in the way of a necessary arthrocentesis. If possible, aspiration through a route other than the possible cellulitis should be preferred. Puncture through an underlying abscess should always be avoided [4, 18, 19, 30, 31].

Category/Grade: Recommendation A, Evidence II-III

13. Should saline be injected in case of a 'dry tap'?

We do not recommend to inject saline because usually there is enough synovial fluid to aspirate in septic arthritis. In case of a dry tap, the needle may wrongly positioned (outside the joint capsule). Using ultrasound may locate the ideal puncture site, targeting maximal fluid collection. However, the fluid may be too dense for the used gauge. In that case, using a larger gauge needle combined with a smaller syringe can generate a greater pressure difference. One can exert manual pressure on the contralateral side of the joint during aspiration to further stimulate fluid collection [32].

Category/Grade: Recommendation A, Evidence III

14. How to puncture not-easily accessible joints (skin incision, fluoroscopy or ultrasound)?

If synovial fluid cannot be obtained with closed needle aspiration, the joint should be aspirated under radiographic guidance (e.g.; ultrasound- or CT-guided puncture) [33].

Category/Grade: Recommendation A, Evidence III

Disclosures

Yvonne Achermann: none Liselotte Coorevits: none, Parham Sendi: none

References

[1]. Geirsson AJ, Statkevicius S, Vikingsson A. Septic arthritis in Iceland 1990-2002: increasing incidence due to iatrogenic infections. Ann Rheum Dis. 2008;67(5):638-43.

[2]. Rutherford AI, Subesinghe S, Bharucha T, et al. A population study of the reported incidence of native joint septic arthritis in the United Kingdom between 1998 and 2013. Rheumatology (Oxford). 2016;55(12):2176-80.

[3]. Kennedy N, Chambers ST, Nolan I, et al. Native Joint Septic Arthritis: Epidemiology, Clinical Features, and Microbiological Causes in a New Zealand Population. J Rheumatol. 2015;42(12):2392-7.

[4]. Long B, Koyfman A, Gottlieb M. Evaluation and Management of Septic Arthritis and its Mimics in the Emergency Department. The western journal of emergency medicine. 2019;20(2):331-41.

[5]. Dubost JJ, Couderc M, Tatar Z, et al. Three-decade trends in the distribution of organisms causing septic arthritis in native joints: single-center study of 374 cases. Joint, bone, spine : revue du rhumatisme. 2014;81(5):438-40.

[6]. Nolla JM, Lora-Tamayo J, Gomez Vaquero C, et al. Pyogenic arthritis of native joints in non-intravenous drug users: A detailed analysis of 268 cases attended in a tertiary hospital over a 22-year period. Semin Arthritis Rheum. 2015;45(1):94-102.

[7]. Goldenberg DL, Brandt KD, Cathcart ES, et al. Acute arthritis caused by gram-negative bacilli: a clinical characterization. Medicine (Baltimore). 1974;53(3):197-208.

[8]. George MD, Cardenas AM, Birnbaum BK, et al. Ureaplasma septic arthritis in an immunosuppressed patient with juvenile idiopathic arthritis. Journal of clinical rheumatology : practical reports on rheumatic & musculoskeletal diseases. 2015;21(4):221-4.

[9]. Sendi P, Zimmerli W, Michot M. Spondylitis and arthritis due to *Mycoplasma hominis:* the case for awareness in undefined pleuropneumonia. Clinical infectious diseases : an official publication of the Infectious Diseases. 2004;39(8):1250-1.

[10]. Taj-Aldeen SJ, Rammaert B, Gamaletsou M, et al. Osteoarticular Infections Caused by Non-Aspergillus Filamentous Fungi in Adult and Pediatric Patients: A Systematic Review. Medicine (Baltimore). 2015;94(50):e2078.
[11]. Steere AC, Strle F, Wormser GP, et al. Lyme borreliosis. Nature reviews Disease primers. 2016;2:16090.
[12]. Arvikar SL, Steere AC. Diagnosis and treatment of Lyme arthritis. Infectious disease clinics of North America. 2015;29(2):269-80.
[13]. Stanek G, Fingerle V, Hunfeld KP, et al. Lyme borreliosis: clinical case definitions for diagnosis and management in Europe. Clin Microbiol Infect. 2011;17(1):69-79.
[14]. Bardin T. Gonococcal arthritis. Best Pract Res Clin Rheumatol. 2003;17(2):201-8.
[15]. Elzein FE, Sherbeeni N. Brucella Septic Arthritis: Case Reports and Review of the Literature. Case reports in infectious diseases. 2016;2016:4687840.
[16]. Hogan JI, Hurtado RM, Nelson SB. Mycobacterial Musculoskeletal Infections. Infectious disease clinics of North America. 2017;31(2):369-82.
[17]. Marth T, Moos V, Muller C, et al. Tropheryma whipplei infection and Whipple's disease. The Lancet infectious diseases. 2016;16(3):e13-22.
[18]. Garcia LS. Clinical Microbiology Procedures Handbook, 3rd Edition: American Society of Microbiology; 2010.
[19]. Miller JM, Binnicker MJ, Campbell S, et al. A Guide to Utilization of the Microbiology Laboratory for Diagnosis of Infectious Diseases: 2018 Update by the Infectious Diseases Society of America and the American Society for Microbiology. Clinical infectious diseases : an official publication of the Infectious Diseases. 2018;67(6):e1-e94.
[20]. Morgenstern C, Renz N, Cabric S, et al. Multiplex Polymerase Chain Reaction and Microcalorimetry in Synovial Fluid: Can Pathogen-based Detection Assays Improve the Diagnosis of Septic Arthritis? J Rheumatol. 2018;45(11):1588-93.
[21]. Achermann Y, Zinkernagel AS. Identifying the Pathogen by Multiplex Polymerase Chain Reaction in Bone and Joint Infections: Challenges and Future. J Rheumatol. 2018;45(11):1497-500.

[22]. Sigmund IK, Holinka J, Gamper J, et al. Qualitative alpha-defensin test (Synovasure) for the diagnosis of periprosthetic infection in revision total joint arthroplasty. The bone & joint journal. 2017;99-B(1):66-72.

[23]. Ruppe E, Lazarevic V, Girard M, et al. Clinical metagenomics of bone and joint infections: a proof of concept study. Scientific reports. 2017;7(1):7718.

[24]. Stirling P, Tahir M, Atkinson HD. The Limitations of Gram-stain Microscopy of Synovial Fluid in Concomitant Septic and Crystal Arthritis. Current rheumatology reviews. 2018;14(3):255-7.

[25]. Uçkay I, Al-Mayahi M, Suvà D, et al. Native Joint Arthritis. Bone and Joint Infections2015. p. 77-91.

[26]. Jorgensen JH, Pfaller MA, Carroll KC, et al. Manual of Clinical Microbiology, Eleventh Edition: American Society of Microbiology; 2015.

[27]. Cohen D, Natshe A, Ben Chetrit E, et al. Synovial fluid culture: agar plates vs. blood culture bottles for microbiological identification. Clin Rheumatol. 2019.

[28]. Zuber TJ. Knee joint aspiration and injection. American family physician. 2002;66(8):1497-500, 503-4, 507.

[29]. Renelt M, Hammer M. [How to do: joint puncture]. Deutsche medizinische Wochenschrift (1946). 2016;141(8):558-61.

[30]. Dooley DP. Aspiration of the possibly septic joint through potential cellulitis: just do it! The Journal of emergency medicine. 2002;23(2):210.

[31]. Costales C, Butler-Wu SM. A Real Pain: Diagnostic Quandaries and Septic Arthritis. Journal of clinical microbiology. 2018;56(2).

[32]. Bhavsar TB, Sibbitt WL, Jr., Band PA, et al. Improvement in diagnostic and therapeutic arthrocentesis via constant compression. Clin Rheumatol. 2018;37(8):2251-9.

[33]. Randelli F, Brioschi M, Randelli P, et al. Fluoroscopy- vs ultrasound-guided aspiration techniques in the management of periprosthetic joint infection: which is the best? La Radiologia medica. 2018;123(1):28-35.
