# Supplementary material for: Guideline for management of septic arthritis in native joints (SANJO)
Source: J Bone Jt Infect. 2023 Jan 12;8(1):29–37. doi: 10.5194/jbji-8-29-2023 (PMC9901514; doi:10.5194/jbji-8-29-2023)
Supplement: The supplement related to this article is available online at: https://doi.org/10.5194/jbji-8-29-2023-supplement. [file jbji-8-29-supplement.zip › Workgroup report 7, ACL-R infection.pdf]

EBJIS guideline Workgroup 7: SANJO after ACL reconstruction

ESSKA & EBJIS recoomendations for the management of infections after ACL-R

Nora Renz (MD)[1,2], Tomislav Madjaravic (MD)[3], Matteo Ferrari (MD,PhD)[4], Sebastian Kopf (MD,PhD)[5] and Daniel Pérez-Prieto (MD, PhD)[6,7]

1. *Charité - Universitätsmedizin Berlin, corporate member of Freie Universität Berlin, Humboldt-Universität zu Berlin, and Berlin Institute of Health, Center for Musculoskeletal Surgery (CMSC), Berlin, Germany*
2. *Department of Infectious Diseases, Bern University Hospital, University of Bern, Bern, Switzerland*
3. *University Hospital for Orthopaedic Surgery Lovran, Rijeka, Croatia*
4. *Humanitas Clinical and Research Center, Milan, Italy*
5. *Centre of Orthopaedics and Traumatology, Brandenburg Medical School Theodor Fontane*
6. *Department of Traumatology and Orthopaedic Surgery. Hospital del Mar – Universitat Autònoma de Barcelona (UAB).*
7. *Catalan Institute for Traumatology and Sports Medicine (ICATME), Hospital Universitari Dexeus. – Universitat Autònoma de Barcelona (UAB).*

ETIOLOGY, RISK FACTORS & PREVENTION

1. Which type of microorganisms are the cause of infection after ACL-R?

Most of the infections after ACL-R are acute postoperative infections[1]. The route of infection in these cases is perioperative. Similar to prosthetic joint infection (PJI), staphylococcal infections are the main cause of postoperative infections, representing 60-90% of all infections after ACL-R[2]. However, in infections after ACL-R, coagulase-negative staphylococci (CNS) are more relevant and they account for up to 70% of staphylococcal infections[3]. Although every joint is at risk of hematogenous seeding during bacteremia- irrespective of previous surgery or present implants- no cases of hematogenous infections have been described in the literature and appear to be rare. Recurrent ACL infections have been described and they can be related to improper debridement, an infected Baker cyst or to incorrect surgical treatment in accordance with Gächter stages[4]. Those very few case-reports about chronic infections are due to either the same bacteria (mostly CNS)[5] or to fastidious and rare microorganisms (mycobacteria, fungi, etc.)[6,7]. Culture negative infections after ACL-R have been described in up to 15-30 % of cases[8,9].

Staphylococci are the main cause of infections after ACL-R. CNS are more frequently observed than PJI cases. Therefore, for empiric antibiotic treatment these bacteria should be considered. It will be discussed in the corresponding section.

2. What is the route of infection of the microorganisms that cause infection?

Although evidence of the microorganisms' origin is limited, several studies have focused on graft colonization during preparation. Harvesting has also given rise to concern about colonization through touching patients' skin or hair follicles[10]. These studies have found similar results with microbial growth in 15-30% of graft cultures[10–12]. Colonization as source of infection is a theory that has not been completely validated. In fact, the rates of colonization are much higher than the rates of infection. However, the "contaminants" described in the aforementioned studies match the most common pathogens after ACL-R, i.e., CNS, *Staphylococcus aureus* and *Cutibacterium* ssp[11,13]. Moreover, Gavriilidis showed that although none of the 10% with positive colonization developed clinically evident infection during the 24 weeks of follow-up, they had higher inflammation markers than the

control group in the postoperative course[14]. The long-term outcome and the potential for the occurrence of delayed low-grade infections is not known for these patients.

Graft harvesting and preparation seem to be a plausible source of contamination. Although the role of this contamination in further clinical infection is not clear, we suggest being extremely cautious during these steps and to prevent unnoticed contamination.

3. Which risk factors have been related to infection after ACL-R?

A previous intraarticular corticoid injection has been clearly related to a high risk of infection after ACL-R [15]. The odds ratio risk for infection after ACL-R for those with a history of steroid injection is up to 27 times higher[16]. This is similar to what has been found for PJI. There are two theories about this risk factor. It is either the immunosuppression produced by the corticosteroid itself or the contamination during puncture (or both combined)[17]. Indeed, prolonged oral steroids or immunosuppression have been associated with higher risk of infection[9].

Multiligament surgeries and concomitant procedures (meniscal suture, multiligamentous injuries or extraarticular procedures) are also at greater risk of infection[16,18]. Surgical time and previous surgeries have been related to increased infection even though multivariate analyses have not been performed in these studies[3].

Professional athletes have been seen to have a higher risk of infection after ACL-R according to Sonnery-Cottet[19]. However, there is concern about the possible bias related to athletes as more serious injuries and previous joint injections are often seen in this population. Recently, a prospective cohort study has shown that athletes do have equal risk for infection after ACL-R.[20]

Diabetes, gender and graft type have not been found to be a consistent risk factor for infection after ACL-R[15,21,22].

Confirmed risk factors for infection after ACL-R are previous corticosteroid injection and concomitant surgical procedures.

4. What is the role of knee puncture in infections after ACL-R?

There is no data about knee puncture after ACL injury and before surgery (before ACL-R). In cases of native joints, the risk of infection is very low (< 0.01% after arthrocentesis) but it is supposed to be higher in cases of knee hemarthrosis after injury due to the inflammatory response[23]. Therefore, we advise doing aspiration only in cases of limited range-of-motion, the inability to activate quadriceps and unbearable pain produced by hemarthrosis. Corticosteroids have been described as risk factors for infection after ACL-R in the previous section. Therefore, we recommend avoiding corticosteroid

injections in patients scheduled for ACL-R. In the case of patients that have received a corticosteroid injection before injury, we recommend postponing surgery (if possible) for 6 months following the recommendations of Conen et al. for native joints[23,24].
As will be covered in the diagnosis section, arthrocentesis is mandatory in any case of increasing pain, redness or effusion *after* ACL-R. Synovial fluid analysis is crucial for diagnosis.

We recommend evacuating post-injury hemarthrosis only in cases of uncontrolled pain and limited range-of-motion. Corticosteroid injection should be avoided for at least 6 months before ACL-R.

5. Is there a preferred graft type to minimize infection after ACL-R?

Graft choice is a controversial issue in the field of ACL-R. In terms of infection risk, it has been widely studied. When it comes to autografts, studies have found a slightly higher risk of infection when hamstrings were used[21,25]. A recent metanalysis seems to prove this[26]. Moreover, when hamstrings are compared to quadriceps tendon autograft, the latter also showed less infection risk[27]. However, several studies that implement new preventive strategies such as the vancomycin soaking technique (see point 8) have reported similar infection rates of close to 0% either in hamstrings or bone-patellar tendon-bone (BTPB) autografts[28]. Nevertheless, those studies were not aimed at comparing the infection rates between BPTB and hamstrings. All in all, it is recommended that the choice of graft be in accordance with the surgeon's experience and patient's characteristics rather than taking infection risk as a basis for decision-making when the vancomycin technique is used. It seems clear that allografts are not at greater risk of infection[29]. There are no randomized controlled trials, but several studies obtain similar results, including a recent metanalysis.[26] Thus, the use of either an autograft or allograft, depending on patient's characteristics and not taking into account infection risk, is recommended[30].

Allograft and autografts do have a similar infection rate. Therefore, we recommend choosing the graft based on patients' characteristics. A hamstring autograft seems to have a greater risk of infection after ACL-R compared to BPTB. However, we recommend using either graft depending on the patient's characteristics when the vancomycin technique is used.

6. What is the value of staphylococcal decolonization as a preventive strategy to avoid infection after ACL-R?

Skin and nose decolonization have become very popular among orthopedics in the recent years[31]. In a recent metanalysis, Weiser et al. stated that it may reduce staphylococcal infections when the carriers are treated before surgery[32]. In addition, economic studies suggest that universal decolonization is cost-effective when compared to screening and treating both permanent and intermittent carriers[33,34]. This

is because up to 30% patients are intermittent carriers that are not identified when they are only screened once[34]. All these studies have been performed in the prosthetic field with patients with an average age of around 70 years-old. Most of the studies focused on *Staphylococcus aureus* in their evaluation. They did not consider coagulase-negative staphylococci even though this is a frequent pathogen in PJI. When it comes to infections after ACL-R, no study about decolonization has been done. The infection rate after ACL-R is lower than in PJI (even lower if the vancomycin technique is used). Therefore, cost-effectiveness could hardly be achieved. However, a 3-day universal decolonization is recommended as a clinical benefit without harm can be expected. There would be a reduction in infections in multiligamentous procedures and even in the in-hospital transmission by reducing the number of carriers.

A 3-day skin and nasal universal decolonization prior to ACL-R is recommended in all hospitals in which it can be implemented, taking into account that this recommendation might not be cost-effective.

7. Should prophylactic intravenous antibiotic be administered before ACL-R?

There is strong evidence in favor of intravenous antibiotic prophylaxis in joint replacement surgery and fracture fixation surgery[35,36]. AlBuhairan, in a pooled analysis of 7 studies with 3,065 total joint arthroplasties, has proven that a single dose of cephalosporine administered between 30 and 60 minutes before incision produces a reduction of the relative risk by 81%[37]. There is conflicting data about prolonged antibiotic prophylaxis after surgery, but it is clear that prolonging it more than 24 hours increases the side effects and can even increase the infection rate[38]. There is paucity of studies that have evaluated the effectiveness of intravenous antibiotic prophylaxis in ACL-R. However, Armstrong and also Carney have found less infection in the antibiotic prophylaxis group[16,39]. The World Health Organization (WHO) recommends antibiotic prophylaxis when a considerable amount of hardware is to be implanted even though it does not specify if ACL-R belongs to this group[40]. Our recommendation for this matter is to give only one single dose of cephalosporin 30-60 minutes before incision, i.e., cefazolin 2g (bodyweight >80kg: 3g) or cefuroxime 1.5g (bodyweight >80kg: 3g). Although the screws or button can be considered as small implants, the graft is avascular and therefore it behaves like a foreign body. Since most ACL-R are performed on an outpatient basis, only a single dose of antibiotic prophylaxis is the best option. Oral postoperative antibiotics must be avoided because of side effects, low bioavailability and the consequent lack of efficacy[41]. In cases of type 1 allergy, prior knee infection, hospitalization or antibiotic therapy, antibiotic prophylaxis can be switched to one dose of 1g of vancomycin (or 15 mg/kg, max. 2500 mg).

We recommend one single dose of cephalosporin 30-60 minutes before ACL-R or one dose of 1 gram of vancomycin in cases of type 1 allergy, prior knee infection, prior hospitalization or prior antibiotic therapy. Postoperative antibiotic (either intravenous or oral) must be avoided.

8. What is the evidence and the recommendation for ACL graft soaking in a vancomycin solution?

The technique of soaking the ACL graft in a vancomycin solution was first described by Vertullo et al. in 2012[42]. A 5mg/ml vancomycin solution is used to soak the graft for 10-15 minutes in order to eradicate contamination and also to use it as an antibiotic reservoir[11,43]. Since then, several studies have been published with similar results. A close to 0% infection rate has been found in all of them, in both the case of a hamstring autograft or BPTB autograft[28,44–47]. A recent metanalysis that included some 3,000 ACL-R using the vancomycin technique has also obtained similar results with an incidence of 0% infections after ACL-R when this technique is used[48]. Additionally, recent studies have proven it to be a safe technique with no increment in re-ruptures and a similar return-to-play time[46,49]. It is a cost-effectiveness technique[46]. However, it is important to remember that all other measures to reduce infection as well as behavior in the operating room must be implemented and cannot be neglected. Moreover, all these studies include primary reconstructions without other procedures in which infection risk is higher.

Other antibiotic soaking techniques, like with gentamicin, have also been described[50]. However, evidence is more limited and the activity against staphylococci is worse than with vancomycin. The resistance of microorganisms to vancomycin is very rare. It has mostly been described in enterococci, which is not a frequent pathogen in infections after ACL-R.

We strongly recommend soaking the ACL graft in a 5mg/ml solution of vancomycin as the technique reduces infection after ACL-R.

DIAGNOSIS

9. What are the clinical signs and symptoms that should raise suspicion of infection after ACL-R?

The signs and symptoms suggestive of infection are often subtle and may be difficult to distinguish from the normal healing process after ACL-R. Increasing or persistent knee pain, tenderness upon slight percussion of the joint, recurrent or persistent knee effusion and systemic symptoms such as fever (> 38.3° C), chills and malaise should call for further investigation[51,52]. However, they are not specific and may also occur in noninfected patients with a large hematoma. Delayed range of motion (ROM) recovery, increased difficulties with physical therapy, increased warmth or swelling, drainage from the incision site/portals (most commonly affected is the tibial tunnel) or any untoward event are suggestive but not specific for infection[18,52,53].

The challenge of late and delayed infection is the indolent presentation of microbial biofilms involving pathogens of low virulence and hence low-grade inflammation[52]. In chronic infection, arthrofibrosis after ACL-R is highly suggestive of infection[54].

Purulent secretion, a sinus tract or intraoperative detection of intraarticular pus are confirmative signs of infection[55].

Suggestive signs and symptoms are delayed ROM recovery, increased warmth or swelling, wound drainage and arthrofibrosis as well as unusual pain and systemic symptoms such as fever and malaise. Confirmative signs are purulent discharge/aspirate, sinus tract communication with the joint and intraoperative intraarticular pus.

10. What classification of infection after ACL-R should be used in clinical and scientific practice?

Since its first publication 1997, the classification by Williams *et al.* has been repeatedly proposed for use in clinical and scientific practice. It differentiates between acute (< 2 weeks), subacute (2 weeks up to 2 months) and late (> 2 months) infection after ACL-R[3]. The basis for these intervals is unclear and there is no clinical impact deduced from the different classifications. In addition, acuity and time of occurrence after surgery are mixed in the previously used classification. We propose differentiating between acute and chronic infection as well as taking into account the pathogenesis along with the time of occurrence. Acute infections arise in the early postoperative period (within 4 weeks after ACL-R). Hematogenous infections may occur at any time even though they are less frequent in ACL-R than joint replacements and are mostly caused by high-virulent pathogens such as *S. aureus*, streptococci and gram-negative rods. Chronic or low-grade infections occur at a later stage and are primarily caused by low-virulent pathogens like coagulase-negative staphylococci, *Corynebacterium* spp. and *Cutibacterium* spp. While chronic septic arthritis is a rarity in native joints, chronic infections are more

common in joints after ACL-R that contain non-vascularized tissue and metal work. In the presence of a foreign material in the joint, the infection is categorized according to the "biofilm age"[56]. Accordingly, periprosthetic joint infections (PJI) with a biofilm less than 3 to 4 weeks of age are considered acute, whereas implant-associated infections exceeding this time interval are categorized as chronic[57,58]. The clinical relevance of this classification is the possible anticipation of the pathogen and respective choice of empiric antibiotic treatment and type of surgical treatment.

We suggest classifying into acute and chronic infections after ACL-R. In the case of a diagnosis within 4 weeks after ACL-R or the new-onset of symptoms of less than 4-weeks duration, the infection is considered acute. On the other hand, chronic infections manifest at more than 4 weeks after ACL-R or with a symptom duration of more than 4 weeks at any time.

11. What is the value of systemic inflammatory markers in the diagnosis of infections after ACL-R?

The specificity of systemic inflammatory markers such as an increased peripheral white blood count, erythrocyte sedimentation rate (ESR) and C-reactive protein (CRP) was found to be limited in the setting of suspected septic arthritis[59]. Furthermore, the sensitivity of systemic inflammatory parameters varies widely between acute and chronic low-grade infections after ACL-R. Only including obvious and florid infections after ACL-R, several studies showed a high sensitivity for CRP[60]. Extrapolated from PJI, infections caused by low-virulent pathogens such as Cutibacterium are expected to cause normal or only mildly elevated CRP in the majority of cases[61]. CRP was shown to be more sensitive and specific for the diagnosis of infection than ESR[62], which is not performed anymore in many institutions in the setting of septic arthritis[63]. The normal CRP course after ACL-R varies widely between individuals, genders, the experience of the surgeon and type of surgery performed[64]. Margheritini *et al.* proposed that a CRP value that does not decrease to normal within 2 weeks of intervention or a secondary CRP rise after an initial decrease are suggestive of infection[63]. However, Ruiz-Ibán observed elevated CRPs showing up to a 5x increase within the first month after ACL-R in uninfected cases. They suggest that a 10-fold increase is very specific for infection (98 % on the 4th postoperative day and 96 % on the 7th postoperative day). One should bear in mind that resorption of hematoma can also cause a high or newly increasing CRP.

We recommend performing CRP as a systemic inflammatory parameter. However, it should be interpreted with caution. We expect high sensitivity in acute infection conditions and low sensitivity with chronic infections. A normal CRP does not exclude infection. Neither does an elevated CRP confirm infection. A secondary increase in CRP in the postoperative course is suggestive of infection as is a 10-fold elevation of the normal value in the first postoperative week.

12. What is the value of imaging in the diagnosis of infections after ACL-R?

Infection after ACL-R is primarily a clinical and laboratory diagnosis. Magnetic resonance imaging may be helpful to exclude other complications that may imitate the signs and symptoms of infection. They include graft impingement, graft ruptures, focal arthrofibrosis, infected Baker's cysts or cystic degeneration of the graft. In addition, it may help to confirm whether there was an inadequate primary debridement in the case of an improper synovectomy that shows remaining synovitis or an involved Baker's cyst that was left in place[65]. MRIs play an important role in chronic infections in which osteomyelitis is frequently present and removal of sequestrum may be required.

The value of imaging in infections after ACL-R is secondary. It may be used to exclude other causes of an unfavorable postoperative course and complications as well as to detect insufficient debridement in surgically pretreated patients with an unfavorable course. In chronic infections, it is mandatory to assess bone involvement and the tissue debridement called for.

13. Which analyses of synovial fluid should be done in case of suspected infection?

Arthrocentesis is considered the preferred diagnostic test in the preoperative setting[18,52,53]. Every joint presenting abnormal features after ACL-R, either in the immediate postoperative course or with new-onset symptoms at a later stage after an uneventful course, should be aspirated if there is an effusion. Then, any suspicion of infection after ACL-R (see 1. and 3. above) must be aspirated. Antibiotic pretreatment may interfere with the microbiological examination and should be withheld until synovial fluid is harvested. The synovial fluid should be sent for microbiology, leukocyte count and differential and crystal analysis, In acute infections, empiric antibiotic treatment should be initiated immediately after sampling and not be withdrawn until the culture results or leukocyte count are available[51]. In chronic infections, the synovial fluid leukocyte count may guide the initiation of empiric antibiotics before revision surgery. However, to date, there is no validated cutoff. In the case of a normal or low leukocyte count in preoperative arthrocentesis, it is acceptable to await the intraoperative results.

***<u>Leukocyte count</u>*:** The determination of the leukocyte count and percentage of granulocytes in synovial fluid is cornerstone in the diagnosis of infections after ACL-R (use ethylenediaminetetraacetic acid (EDTA) tubes to prevent coagulation). Although a leukocyte count consistent with infection is considered confirmative criterion by many authors[1,66], there are no uniform cut-offs of leukocyte counts in synovial fluid in joints after ACL-R. Distinguishing infection from aseptic inflammation in native and prosthetic joints thresholds have been debated for decades. Proposed thresholds vary widely and range from 2.000-10.000/ul in prosthetic joints and 17.500-50.000/µl in native joints[56,57,59]. In infections after ACL-R, low grade infections caused by low-virulent pathogens are common.

Therefore, it is not advisable to use the same threshold as proposed for native joints and suggest using lower cut-offs to avoid underdiagnosing infection. Consider that lower counts, especially in chronic infections, do occur. This fact contrasts with most previously published recommendations for infections after ACL-R. They did not take in account the existence of low-grade infections with a low level of inflammation. Furthermore, the physiological course of leukocytes in the synovial fluid is unknown in the postoperative course after ACL-R. There is no established and validated cut-off for absolute counts. However, a normal leukocyte count (< 2.000 /µl) rules out infection in most cases. A granulocytes percentage of >90% showed a high likelihood ratio for infection[52].

Microbiology: Traditionally, conventional culturing on agar plates is considered the gold standard for pathogen detection in septic arthritis[59]. Inoculation of synovial fluid into pediatric blood culture bottles showed a higher diagnostic yield in terms of pathogen detection compared to the conventional agar plate method in septic arthritis[67]. Gram stains show high specificity but low sensitivity (29 to 50 %) and are inferior to culture methods in septic arthritis[59]. Due to the high specificity, it may be used as a rule-in test in the case of a positive result. In patients pretreated with antimicrobials before joint aspiration with negative culture results, bacterial DNA can be identified by polymerase chain reaction (PCR)[68].

***Biomarkers*:** Several markers such as synovial fluid glucose, protein and lactate dehydrogenase were assessed in the setting of native joints and none of them have shown promising enough results to enter the guidelines[59]. Several reports suggest that D-lactate assessment in synovial fluid could be a specific and sensitive test for the early diagnosis of bacterial infection of native and prosthetic joints[69–71]. To our knowledge, no studies have been performed on joints after ACL-R. Accordingly, calprotectin and alpha-defensins were evaluated in both native and prosthetic joints[72–74]. However, none of the biomarkers has yet been validated in a representative study for infections after ACL-R.

Crystals: The coincidence of crystal arthropathy and infection in joints after ACL-R is probably low with regards to the population undergoing ACL-R. However, particularly in cases with inconclusive diagnostics and negative cultures in presence of a high leukocyte count it represents a differential diagnosis. Polarized light microscopy should be performed to exclude (concomitant) crystal arthropathy if the clinical signs and symptoms occur at a late stage after an uneventful course.

Every painful or otherwise suspicious joint should immediately be aspirated. Antibiotic treatment should be withheld until synovial fluid has been harvested. We recommend doing a leukocyte count and differential (percentage of polymorphonuclear cells) as well as microbiological cultures (inoculated in pediatric blood culture bottles and plated on solid media). In cases of acute infection at a later stage after ACL-R, crystal arthropathy should be excluded through microscopy. Additional tests such as molecular tests, D-lactate and other biomarkers are not (yet) considered standard, but experimental.

14. What intraoperative diagnostics should be performed

Histopathology: To our knowledge, there is no data available on specific histopathological changes in infections after ACL-R. Nevertheless, microscopic tissue changes of the affected joint should be assessed by an experienced histopathologist. In native joints, a synovialitis score was elaborated in order to facilitate the discrimination of rheumatoid, septic and non-septic arthritis[75,76]. Consistent morphology may corroborate the clinical diagnosis of infection in cases of culture-negative infections. In addition, rare differential diagnoses such as crystal arthropathy and mycobacterial septic arthritis may be excluded. Normal histopathological findings do not exclude (low grade) infection. Additionally, sampling errors might mitigate the real entity.

Microbiology: To collect different types of species, we recommend harvesting another intraoperative sample of synovial fluid for culture. In addition, multiple samples of macroscopically affected tissue (synovial lining, graft, femoral and tibial tunnel) should be taken[52]. As extrapolated from PJI, (3 to) 5 tissue samples increase the diagnostic yield and facilitate the interpretation of the results if low-virulent skin commensals are isolated[77].

In cases of removal or exchange of the graft and/or the fixation devices, the graft should be sent for microbiological culture and foreign material (fixation devices) to sonication. Sonication has been shown to be an efficient diagnostic method by dislodging the biofilm from the surface in PJI and implant-associated infections[78,79].

It is important to notify the microbiology laboratory that there is non-vascularized and foreign material involved in the process so that prolonged incubation (for 10 to 14 days) can be carried out and a more extensive interpretation of the results can be elaborated, especially in presence of a typical skin commensal[51]. As used for PJI, isolation of the identic pathogen in at least 2 different samples in cases of low-virulent pathogens (e.g. *Cutibacterium* spp., coagulase-negative pathogens, *Corynebacterium* spp.) and in one sample in case of high-virulent pathogen is considered significant and confirms infection after ACL-R. In cases of non-significant pathogen detection of low-virulent pathogens (e.g. *S. epidermidis* in 1 specimen), the result should be interpreted in the context of non-microbiological criteria.

We recommend collecting and analyzing:

- ***Synovial fluid for microbiological analysis (blood cultures bottles and native vials)***
- ***3 to 5 tissue samples from representative and macroscopically infected tissue***
- ***at least one sample for histopathological examination***

- ***in case of graft removal/exchange, sending the fixation devices (foreign material) to sonication and the graft for conventional culture***

The laboratory should be notified about the type of infection and that a foreign body is involved in the infection to ensure prolonged incubation of the samples.

Confirmative criteria for infection after ACL-R are intraarticular purulence, purulent secretion or sinus tract communication with the joint, positive cultures of tissue, synovial fluid or sonication and/or histopathology consistent with acute infection.

15. Should blood cultures be collected in any case of infection after ACL-R?

In general, infections after ACL-R are of perioperative pathogenesis and blood cultures are not needed. However, every joint is at risk of hematogenous seeding in cases of (prolonged and high-load) bacteremia irrespective of a previous history of ACL-R. Accordingly, secondary infection can occur at any stage after ACL-R (even years or decades after surgery). It results in an acute infection after ACL-R that occurs after an uneventful course, usually concomitantly with systemic signs and symptoms of bloodstream infection like fever, chills, malaise etc. If hematogenous infection is diagnosed, investigation of cause (i.e. echocardiography, othropantomogram, urinalysis, conventional lung X-ray etc), depending on the pathogen, is indicated.

In the case of an acute onset of local and systemic signs and symptoms of knee infection at any time after an uneventful period after ACL-R, blood cultures should be collected to exclude hematogenous infection.

SURGICAL TREATMENT

16. Is surgical treatment necessary for an infection after ACL-R? Which type of surgery is called for?

The key aims in the management of infection after ACL-R are successful infection clearing and the complete functional recovery of the knee joint[66,80,81]. There are no prospective randomized controlled trials that compare surgical and conservative treatment for infection after ACL-R. Indeed, there are studies on native septic arthritis that showed no differences between needle aspiration and surgical debridement[82]. However, the primary endpoint of these studies was infection cure without any assessment of functional outcomes or cartilage damage[82,83].

Despite the lack of evidence, the present group recommends arthroscopic debridement for the following reasons:

- Surgical treatment is necessary to wash out proteolytic enzymes and toxins which cause chondrocyte degeneration[84,85].
- Prompt evacuation of the joint by means of arthroscopic revision reduces the bacterial load and intraarticular pressure[1].
- Arthroscopy allows for cartilage evaluation in accordance with the Gächter classification and the evaluation of graft stability and viability[86,87].
- Compared to open surgery, arthroscopy has proven to be less invasive and facilitates a faster recovery, without compromising the cure rate[88].

However, open debridement is indicated in cases when there is subchondral bone involvement (Gächter IV)[86].

Clinical staging (Gachter classification)	***Intraoperative (arthroscopic) spread of inflammatory process***
Stage 1	***Turbid effusion, hyperemic synovia***
Stage 2	***Purulent effusion, fibrinous appositions, hypertrophic synovia***
Stage 3	***Synovial adhesion, necrotic areas of synovia and cartilage***
Stage 4	***Cartilage necrosis, bone erosion, osteolysis***

Although there are reports of successful treatment with percutaneous drainage or bedside arthrocentesis, this surgical alternative is reserved for selected cases which are not suited to surgery. These less invasive methods are considered inferior as removal of infection residue and mediators is insufficient.

We recommend performing arthroscopic debridement in combination with antibiotic therapy as the primary therapeutic option in every patient. In the rare case of inoperability, repeated needle aspiration might be an alternative.

17. What is the best time for surgical treatment?

Intraarticular infection can lead to graft failure, joint stiffness and chondral damage[89]. Delaying surgical treatment leads to chondrocyte degradation and ACL graft insufficiency, which can cause functional disability. Therefore, surgical treatment should be performed immediately after infection diagnosis or in cases in which there is a high suspicion.

Delayed treatment and prolonged intra-articular infection have been shown to compromise graft function[27]. The cartilage loses more than half of its glycosaminoglycan and collagen if surgical treatment is not initiated within 7 days of the onset of symptoms[84,90].

Delayed treatment, late presentation (more than 30 days after surgery), and virulent microorganisms are risk factors for prolonged treatment and the need for an arthrotomy and graft and hardware removal[8,80,91–93]. Dave et al. reported a correlation between the number of hours between the onset of symptoms and index surgery and the need for multiple procedures[66].

We recommend surgical treatment as soon as a clinical suspicion is established in cases with acute symptoms or in the early postoperative setting, even if the microbiological results are still pending.

18. How many arthroscopic procedures should be performed?

Wang et al. reported that 60% of the patients were successfully treated with a single arthroscopic debridement. Repeated debridement was carried out because of persistent clinical symptoms, fever or increased CRP levels [8]. According to several studies, another arthroscopic debridement should be performed in case of persistent septic arthritis with no positive evolution of clinical (i.e. local inflammatory changes, persistent wound drainage, fever) and laboratory (i.e. persistent or secondarily increased CRP) signs[80,89]. Binnet et al. showed that an average of 2.66 procedures was required to eradicate infection[94]. In previous reports, the graft along with its original fixation material remained in place when the graft was considered functional and did not block knee motion[95]. Indeed, several reports have shown that about 4 out of 5 anterior cruciate ligament (ACL) grafts can be successfully salvaged with multiple debridement procedures. However, Vertullo et al. suggest that a failure to see improvement after 2 arthroscopic irrigations implies that the bacteria have formed a biofilm and the graft is non-viable or that osteomyelitis has also involved the femoral tunnel[42]. Calvo et al. repeated joint lavage several times[89]. After the third lavage, the graft and implants were removed because of a

persistent clinical infection, macroscopic graft damage and elevated inflammation parameters. However, those patients had presented more than one week after infection started. In cases with persistent infection, magnetic resonance imaging should be considered to evaluate the possible involvement of the tunnels and to detect cavities or abscess formation[92].

McAllister et al. were able to retain the graft by managing persistent infections with two to four subsequent debridement procedures, but all four patients developed degenerative changes at a mean of 36 months[95]. One factor that is crucial for graft viability is an early diagnosis since patients diagnosed after 7 days from the onset of symptoms had a higher graft removal rate[8,93]. Delayed treatment might weaken the graft, delay integration or lead to insufficiency[27].

Scheduled debridement should not be performed in patients with a favorable course after the first debridement.

Additional debridement is indicated if the clinical course is not favorable. Unfavorable determinants include increasing pain, fever or persistent or secondarily increased CRP without any other explanation (e.g. nosocomial infection), a persistent discharge from the portal or persistent local signs of inflammation. In cases with an uneventful course, repeated arthroscopic debridement is not needed. If the course is not adequate after the 3rd debridement, graft and hardware removal should be considered. MRI may help in identifying the cause of persistent infection in those cases.

19. What is the role of the Gächter classification in infections after ACL-R?

Intraoperative arthroscopic findings in native septic arthritis were described and stratified by Gächter et al. and can be seen in Table 1[86].This classification is a useful guide to the therapeutic measures to take. It recommends mild arthroscopic debridement without synovectomy in stage I (mainly lavage) because the synovial membrane acts as a barrier and provides the joint with nutrients and antibiotics and it is well vascularized. Moreover, complete removal when it is not affected by infection will provoke bleeding and hematoma. Aggressive arthroscopic debridement is recommended in stages II and III and open debridement (arthrotomy) in stage IV. This classification also predicts the need for further debridement, 52% in stage II and 75% in stage III[86].

Although several authors recommend choosing the surgical strategy according to the Gächter stage, there is no study that has validated its utility in infections after ACL-R. However, those studies which showed excellent outcomes even with repeated debridement reported only Gächter stage I and II ACL-R infections[96].

Nevertheless, we recommend the use of the Gächter classification as a surgical and prognostic guide in infections after ACL-R. It should be considered in combination with graft viability and hardware stability.

We recommend the Gächter classification along with graft condition to select the optimal surgical treatment for ACL-R infections.

20. In which situations should the graft and hardware be removed? When can the new ACL-R be performed in cases of graft and hardware removal?

Graft removal is considered when multiple debridement procedures fail to control the infection, in loosened fixation devices or with graft insufficiency[66]. Another reason for graft and hardware removal is bony involvement of the tibia or femur (Gächter IV)[27].

According to several studies, a new ACL reconstruction is performed from three to six months following graft removal[26,52,66,90]. However, this approach was based on concomitant osteomyelitis rather than infections after ACL-R.

Based on the available evidence of PJI and native septic arthritis, a new graft can be reconstructed in a shorter period (6 weeks) if the following considerations are fulfilled[58]:

- No bone involvement (no osteomyelitis)
- Good clinical evolution
- Decreasing CRP (no need to be normal)
- No difficult-to-treat infections caused by a microorganism that is resistant to biofilm-active antibiotics (see antibiotic section for information)

In the case of a new ACL-R, tissue cultures from the synovial membrane and bone tunnels must be obtained during surgery. Histopathology of the tunnels might be of help in ruling out osteomyelitis. Keeping patients under antibiotics until the results of intraoperative diagnostics are available is recommended.

If these requirements are not met, the new ACL-R must be delayed, either after additional debridement procedures or when the prolonged antibiotic treatment (i.e. osteomyelitis treatment) is completed.

Graft and hardware must be removed in the case of multiple debridement procedure failures and/or hardware loosening/graft insufficiency. We recommend that graft reimplantation be performed after 6 weeks in selected cases in cases of graft and hardware removal.

SYSTEMIC ANTIMICROBIAL TREATMENT

21. What are the most common pathogens that cause infection after ACL-R?

The most commonly isolated pathogens that cause infections after ACL-R are staphylococci, with coagulase-negative staphylococci being the most common followed by Staphylococcus aureus[2,8,53,66]. Depending on the local epidemiology, the percentage of methicillin-/oxacillin-resistant staphylococcal strains vary widely. Other causative agents are anaerobes such as Peptostreptococcus spp. and Cutibacterium (formerly Propionibacterium) spp., gram-negative rods (Enterobacteriaceae, Pseudomonas aeruginosa), streptococci and enterococci[80,97]. Fungi and mycobacteria are considered very rare pathogens in infections after ACL-R.

Most common causative pathogens of infections after ACL-R are coagulase-negative staphylococci and S. aureus. Other pathogens include anaerobes, gram-negative rods, streptococci and enterococci.

22. Which antimicrobial agents are recommended for empirical treatment until culture results are available?

To cover the most common pathogens that cause infections after ACL-R, we recommend starting an intravenous (i.v.) antimicrobial treatment with a beta lactam/beta lactam inhibitor combination [e.g. ampicillin/sulbactam 3x3g i.v. or amoxicillin/clavulanic acid 4x2.2g (or 4x1.2g, depending on availability)]. As coagulase-negative staphylococci are oxacillin/methicillin-resistant in the majority of cases (variable epidemiology), the addition of vancomycin (according to through level, target 15 to 20mg/l) or daptomycin 1x8-10mg/kg i.v. is suggested until culture results are available. In case of allergy to penicillin (non-type 1), we suggest replacing the beta lactam antibiotic with a second-generation cephalosporin (e.g. cefuroxime 3x1.5g i.v.). In case of a type 1 allergy (anaphylaxis, Quincke's edema), we suggest using daptomycin 1x8-10mg/kg as a monotherapy. It is preferred over vancomycin due to its bactericidal activity, lack of toxicity and its immediate attainment of therapeutic levels. Regarding the substances used in previous studies, we recommend against the use of 1st or 3rd generation cephalosporins[15,66] due to their narrow spectrum or (flu-)cloxacillin and gentamicin[52] due to their toxicity as an empirical treatment. Indeed, the antimicrobial treatment should be targeted to the pathogen as soon as the causative agent and its susceptibility are available.

We advise against administering rifampin as an empirical treatment as resistance may occur when used improperly.

**1. choice**	**Non-type 1 penicillin allergy**	**Type 1 penicillin allergy**
Ampicillin/sulbactam 3 x 3 g<br>or<br>Amoxicillin/clavulanic acid 4 x 1.2 - 2.2g<br>PLUS<br>Vancomycin 2 x 15 mg / kg<br>or<br>Daptomycin 1 x 8 mg / kg	Cefuroxime 3 x 1.5 g<br>PLUS<br>Vancomycin 2 x 15 mg / kg<br>or<br>Daptomycin 1 x 8 mg / kg	Daptomycin 1 x 8 mg / kg

23. When should antimicrobial treatment be started?

As soon as joint fluid has been obtained, prompt intravenous antibiotic therapy should be given in cases with strong clinical evidence of acute infection[15,52,53]. In this situation, microbiology and leukocyte count results should not be awaited. In cases of chronic infection, the decision whether to start antimicrobials before revision surgery or after harvesting samples intraoperatively may be based on leukocyte count. However, there is no validated cutoff to date (see also section "Diagnosis", 5. Leukocyte count). In the case of a normal leukocyte count, infection is unlikely, and no antibiotics should be started until intraoperative samples are harvested.

Empirical antibiotic treatment should be initiated immediately after joint aspiration if infection is suspected.

24. Is the use of biofilm-active agents indicated in infections after ACL-R?

While no biofilm-active treatment is needed in septic arthritis of the native joint, the use of biofilm-active antibiotics in implant-associated joint infections has been shown to be superior[98]. In infections after ACL-R, a native joint with non-vascularized (graft) and foreign (fixation device) material is involved in infection. Guidelines based on either clinical trials or animal model studies recommend the use of rifampicin in combination with another antibiotic for the treatment of staphylococcal infections[99–102] and ciprofloxacin for gram-negative pathogens[101]. In this context, we recommend using biofilm-active treatment -if available- in the case of treatment scenarios in which the graft is exchanged or retained. Pérez-Prieto et al. showed a high rate of treatment success using a rifampin-fluoroquinolone combination in staphylococcal infections after ACL-R[2]. Based on theoretical considerations and in vitro studies, the same applies to infections caused by cutibacteria[103]. The optimal time-point for starting rifampicin therapy is still debated. We advocate starting rifampicin after all drains have been removed and the wound is dry, as recommended for periprosthetic joint infections.

Although clinical evidence is weak, based on theoretical considerations and extrapolation from other implant-associated infections, the use of biofilm-active antibiotics in infections with any grafts and fixation devices in place is advisable.

25. What is the optimal treatment duration for ACL-R-infections?

There is a controversy regarding the duration of antibiotic treatment and when to switch from intravenous to oral therapy. There are no randomized controlled studies addressing this issue. In native septic arthritis, the duration of antimicrobial treatment depends on the organism isolated and the clinical response to the chosen antibiotic. The duration of treatment is generally 2 to 6 weeks[104]. In infections after ACL-R, it was 2 to 12 weeks in previously published case series[1,8]. Intravenous treatment was given for 5 days to 6 week[1,2,96]. In most reports, an adequate clinical response and a decrease in C-reactive protein (CRP) are prerequisite for a switch to oral treatment or discontinuation of antibiotics[8,52,81,96]. Overall, antibiotic treatment is maintained for a minimum of 4 to 6 weeks[1,3,51–53]. In a recent study, a good clinical outcome was seen with oral treatment started at a mean of 5 days (range, 4–7) after surgery and a total antibiotic treatment lasting an average of six weeks[2]. Recent landmarks studies corroborated the trend towards shorter i.v. treatment durations in severe infections such as bone and joint infections and infective endocarditis[105,106].

A 1-week (up to 2 weeks) i.v. treatment regimen is suggested. It should be followed by oral treatment for another 4 to 5 weeks. It would be preferable to do it with bactericidal agents with good bioavailability and bone penetration as well as biofilm-activity if avascular tissue and fixation devices are in situ. The conditions for switching to an oral treatment are a good clinical response with a decrease in local inflammatory signs and CRP trending towards normal values.

We suggest a 1 week (up to 2 weeks) of intravenous treatment followed by oral treatment for another 4-5 weeks, preferably with bactericidal agents with good bioavailability and bone penetration as well as biofilm-activity if avascular tissue and fixation devices are in situ. The conditions for switching to oral treatment are a good clinical response with nearly normal CRP values.

26. What is the recommended targeted antimicrobial regimen for the most common pathogens?

The recommended pathogen-specific treatment extrapolated from the treatment of septic arthritis and periprosthetic joint infections are shown below[57,104,107]. In culture negative cases, we advise giving broad spectrum (i.e., empiric) intravenous antibiotics followed by a biofilm-active combination covering the most common pathogens, i.e., rifampin and fluoroquinolones (see below).

Microorganism	Antibiotic (check pathogen susceptibility before)	Dose (blue: renal adjustment needed)	Route
***Staphylococcus* spp.**			
- Oxacillin-/methicillin-susceptible	Flucloxacillin[a]	4 x 2g	i.v.
	+/- Fosfomycin[c]	3 x 5g	i.v.
	for 1-2 weeks*, followed by (according to susceptibility)		
	Cotrimoxazole	3 x 960mg	p.o.
	or		
	Rifampin[d] + either	2 x 450mg	p.o.
	- Levofloxacin or	2 x 500mg	p.o.
	- Cotrimoxazole or	3 x 960mg	p.o.
	- Doxycycline or	2 x 100mg	p.o.
	- Fusidic acid	3 x 500mg	p.o.
- Oxacillin-/methicillin-resistant	Daptomycin or	1 x 8mg/kg	i.v.
	Vancomycin[b]	2 x 15mg/kg	i.v.
	+/- Fosfomycin[c]	3 x 5g	i.v.
	for 1-2 weeks*, followed by an oral therapy as above		
***Streptococcus* spp.**	Penicillin G[a] or	4 x 5 million U	i.v.
	Ceftriaxone	1 x 2g	i.v.
	for 1-2 weeks*, followed by:		
	Amoxicillin or	3 x 1000mg	p.o.
	Levofloxacin	2 x 500mg	p.o.
***Enterococcus* spp.**			
- Penicillin-susceptible	Ampicillin or Amoxicillin	4 x 2g	i.v.
	+ Gentamicin[e] or	1 x 3mg/kg	i.v.
	+ Ceftriaxone[f]	2 x 2g	i.v.
	+/- Fosfomycin[c]	3 x 5g	i.v.
	for 1-2 weeks*, followed by:		
	Amoxicillin	3 x 1000mg	p.o.
- Penicillin-resistant or allergy to penicillin	Vancomycin[b] or	2 x 15mg/kg	i.v.
	Daptomycin	1 x 10mg/kg	i.v.
	+ Gentamicin[e]	1 x 3mg/kg	i.v.
	+/- Fosfomycin[c]	3 x 5g	i.v.
	for 1-2 weeks, followed by:		
	Linezolid	2 x 600 mg	p.o.
**Gram-negative rods**			
- Enterobacteriaceae (*E. coli, Klebsiella, Enterobacter,* etc.)	Ceftriaxone or	1 x 2g	i.v.
	Piperacillin/tazobactam or	3 x 4.5g	i.v.
	Meropenem	3 x 1g	i.v.
	for 1-2 weeks followed by:		
	Ciprofloxacin	2 x 750mg	p.o.
- Nonfermenters (*Pseudomonas aeruginosa, Acinetobacter* spp.)	Piperacillin/tazobactam or	4 x 4.5g	i.v.
	Meropenem or	3 x 2g	i.v.
	Ceftazidime or Cefepime +	3 x 2g	i.v.
	Tobramycin[g]	1 x 3-5mg/kg	i.v. i.v.
	(or Gentamicin)	(1 x 3mg/kg)	
	for 2 weeks, followed by:		
	Ciprofloxacin	2 x 750mg	p.o.
**Anaerobes**	Penicillin G[a] or	4 x 5 million U	i.v.
	Ceftriaxone	1 x 2g	i.v.
	for 1-2 weeks, followed by:		

- Gram-positive (*Cutibacterium, Peptostreptococcus, Finegoldia* spp.)	Rifampin[d] +<br>Levofloxacin or<br>Amoxicillin	2 x 450mg<br>2 x 500mg<br>3 x 1000mg	p.o.<br>p.o.<br>p.o.
Candida spp.<br>- Fluconazole-susceptible	Caspofungin[h]<br>Anidulafungin<br>for 1-2 weeks, followed by:<br>Fluconazole	1 x 70mg<br>1 x 100mg (1. day 200 mg)<br><br>1 x 400mg	i.v.<br>i.v.<br><br>p.o.
- Fluconazole-resistant	Individual (e.g. with voriconazole[i] 2 x 4mg/kg p.o.)		
**Culture-negative**	Ampicillin/sulbactam[a]<br>for 2 weeks, followed by:<br>Rifampin[d] +<br>Levofloxacin	3 x 3g<br><br>2 x 450mg<br>2 x 500mg	i.v.<br><br>p.o.<br>p.o.

Footnotes

[a] Non-type 1 **penicillin allergy** (e.g. skin rash): cefazolin (3x2g i.v.). In the case of anaphylaxis (type 1 allergy such as Quincke´s edema, bronchospasm, anaphylactic shock) or cephalosporin allergy: vancomycin (2x15mg/kg i.v.) or daptomycin (1x8mg/kg i.v.); Ampicillin/sulbactam is equivalent to amoxicillin/clavulanic acid (4x2.2g i.v.)

[b] Check **Vancomycin** through concentration (take blood before next dose) at least 1x/week; First concentration before 4th dose; therapeutic range: 15-20µg/ml

[c] Fosfomycin can be given 3x5g or 2x8g. Fosfomycin is not available in all countries and not in the same dosage. Monitor regularly serum electrolytes

[d] Add it already to intravenous treatment as soon as wounds are dry and drains removed and the graft is retained or exchanged; in patients aged >75 years, rifampin is reduced to 2x300mg p.o.

[e] Give only, if enterococcus is tested **gentamicin high-level (HL)** susceptible (consult microbiology laboratory). In gentamicin HL-resistant *E. faecalis*: gentamicin is exchanged with ceftriaxone (2x2g i.v.)

[f] Valid only for *Enterococcus faecalis*

[g] Combination treatment only indicated in multi-resistant gram-negative bacteria

[h] After a loading dose of 70mg on day 1, **reduce to 50mg** in patients weighing <80kg from day 2

[i] Loading dose 2x6mg/kg, then 2x4mg/kg, measure through level (1-5µg/ml)

REHABILITATION

27. Are functional outcomes impaired in patients after an ACL-R infection?

A satisfactory result after treatment of an ACL-R infection should be defined as good functional scores, articular cartilage architecture preserved, ACL graft function preserved, full range of motion (ROM) restored or a return to previous level of activity1.

Some studies seem to suggest that knee function is impaired after infection, and the results are inferior to those without an infection after ACL reconstruction[19,91,93]. Stiffness and arthrofibrosis are the most common reason for functional limitation[93,108]. Therefore, additional surgeries like arthroscopic arthrolysis are quite frequent even though they can be considered part of the infection treatment. Even after those procedures, a flexion or extension deficit is seen in infected patients, albeit without clinical relevance[3,66,91,95]. In that sense, Abdel-Aziz et al. found loss of range in flexion and extension in 21% of the patients with an infection following ACL-R[96].

On the other hand, Boström et al. studied functional outcomes in 27 patients after infected ACL-Rs with 60 months of follow-up and found no inferior objective knee function or lower degree of patient satisfaction[109]. However, the patients did require a longer rehabilitation period[66].

Interestingly enough, when the ACL-R infection went undetected for less than 5 days, no statistically significant differences were noted in the mean Lysholm, IKDC, or KOOS scores between the patients with a successfully treated infection and matched patients with an ACL reconstruction without infection[3].

Moreover, data from studies with higher rates of graft retention showed greater range-of-motion as well as similar Lysholm scores when compared with data reported from non-infected cases[19].

Delayed diagnosis and treatment might preclude poor functional recovery[27]. Therefore, a delay in treatment could lead to graft failure, articular cartilage damage and joint dysfunction[92].

Functional scores might be impaired in patients with infection after ACL-R. Early diagnosis and treatment are crucial to preventing arthrofibrosis, stiffness and to preserve cartilage.

28. Which rehabilitation protocol is appropriate in cases of infection after ACL-R?

Josep Trueta was the first to suggest immobilization of the limb when dealing with infected wounds[110]. Since then, that protocol has been extrapolated to all orthopedic infections[111]. However, it is important to state that Trueta's studies were carried out in the early 1940s and were mostly related to open fractures in war injuries. What is crucial to healing in a septic non-union might be harmful in terms of

infections after ACL-R. This is because the main sequalae in arthroscopic infections is a decrease in ROM, as previously stated.

Physical therapy should be focused on preventing stiffness and regaining range-of-motion (ROM)[80]. A loss of flexion, with a mean loss of 5.8°, was reported by 13 studies[66].

Thus, it is necessary to start promoting ROM exercises as soon as possible without limitations in range of motion after arthroscopic irrigation and debridement[93]. Moreover, muscle training (especially isometric exercises) can be started in the early postoperative days, between day 1 to day 5[1,19]. For instance, Calvo et al. proposed a rehabilitation program with adjustment depending on the symptoms and pain[89]. Additionally, weightbearing might be allowed, as tolerated, from the early postoperative period[81,93,112].

In patients with an infection after ACL-R, the same rehabilitation protocols are recommended as for ACL-R to prevent arthrofibrosis. It is advisable to start as soon as the symptoms suggestive of infection are resolved. Of course, there should be adjustments based on the symptoms and pain experienced. Muscle strength and ROM should be the physical therapist's target.

SUMMARY OF RECOMMENDATIONS

- **Staphylococci are the main cause of infections after ACL-R. CNS are more frequently observed than PJI cases. Therefore, for empiric antibiotic treatment these bacteria should be considered. It will be discussed in the corresponding section.**
- **Graft harvesting and preparation seem to be a plausible source of contamination. Although the role of this contamination in further clinical infection is not clear, we suggest being extremely cautious during these steps and to prevent unnoticed contamination.**
- **Confirmed risk factors for infection after ACL-R are previous corticosteroid injection and concomitant surgical procedures.**
- **We recommend evacuating post-injury hemarthrosis only in cases of uncontrolled pain and limited range-of-motion. Corticosteroid injection should be avoided for at least 6 months before ACL-R.**
- **Allograft and autografts do have a similar infection rate. Therefore, we recommend choosing the graft based on patients' characteristics. A hamstring autograft seems to have a greater risk of infection after ACL-R compared to BPTB. However, we recommend using either graft depending on the patient's characteristics when the vancomycin technique is used.**
- **A 3-day skin and nasal universal decolonization prior to ACL-R is recommended in all hospitals in which it can be implemented, taking into account that this recommendation might not be cost-effective.**
- **We recommend one single dose of cephalosporin 30-60 minutes before ACL-R or one dose of 1 gram of vancomycin in cases of type 1 allergy, prior knee infection, prior hospitalization or prior antibiotic therapy. Postoperative antibiotic (either intravenous or oral) must be avoided.**
- **We strongly recommend soaking the ACL graft in a 5mg/ml solution of vancomycin as the technique reduces infection after ACL-R.**
- **Suggestive signs and symptoms are delayed ROM recovery, increased warmth or swelling, wound drainage and arthrofibrosis as well as unusual pain and systemic symptoms such as fever and malaise. Confirmative signs are purulent discharge/aspirate, sinus tract communication with the joint and intraoperative intraarticular pus.**
- **We suggest classifying into acute and chronic infections after ACL-R. In the case of a diagnosis within 4 weeks after ACL-R or the new-onset of symptoms of less than 4-weeks duration, the infection is considered acute. On the other hand, chronic infections manifest at more than 4 weeks after ACL-R or with a symptom duration of more than 4 weeks at any time.**

- **We recommend performing CRP as a systemic inflammatory parameter. However, it should be interpreted with caution. We expect high sensitivity in acute infection conditions and low sensitivity with chronic infections. A normal CRP does not exclude infection. Neither does an elevated CRP confirm infection. A secondary increase in CRP in the postoperative course is suggestive of infection as is a 10-fold elevation of the normal value in the first postoperative week.**
- **The value of imaging in infections after ACL-R is secondary. It may be used to exclude other causes of an unfavorable postoperative course and complications as well as to detect insufficient debridement in surgically pretreated patients with an unfavorable course. In chronic infections, it is mandatory to assess bone involvement and the tissue debridement called for.**
- **Every painful or otherwise suspicious joint should immediately be aspirated. Antibiotic treatment should be withheld until synovial fluid has been harvested. We recommend doing a leukocyte count and differential (percentage of polymorphonuclear cells) as well as microbiological cultures (inoculated in pediatric blood culture bottles and plated on solid media). In cases of acute infection at a later stage after ACL-R, crystal arthropathy should be excluded through microscopy. Additional tests such as molecular tests, D-lactate and other biomarkers are not (yet) considered standard, but experimental**.
- **We recommend collecting and analyzing:**
  - **Synovial fluid for microbiological analysis (blood cultures bottles and native vials)**
  - **3 to 5 tissue samples from representative and macroscopically infected tissue**
  - **at least one sample for histopathological examination**
  - **in case of graft removal/exchange, sending the fixation devices (foreign material) to sonication and the graft for conventional culture**

  The laboratory should be notified about the type of infection and that a foreign body is involved in the infection to ensure prolonged incubation of the samples.

  Confirmative criteria for infection after ACL-R are intraarticular purulence, purulent secretion or sinus tract communication with the joint, positive cultures of tissue, synovial fluid or sonication and/or histopathology consistent with acute infection.
- **In the case of an acute onset of local and systemic signs and symptoms of knee infection at any time after an uneventful period after ACL-R, blood cultures should be collected to exclude hematogenous infection.**
- **We recommend performing arthroscopic debridement in combination with antibiotic therapy as the primary therapeutic option in every patient. In the rare case of inoperability, repeated needle aspiration might be an alternative.**

- **We recommend surgical treatment as soon as a clinical suspicion is established in cases with acute symptoms or in the early postoperative setting, even if the microbiological results are still pending.**
- **Additional debridement is indicated if the clinical course is not favorable. Unfavorable determinants include increasing pain, fever or persistent or secondarily increased CRP without any other explanation (e.g. nosocomial infection), a persistent discharge from the portal or persistent local signs of inflammation. In cases with an uneventful course, repeated arthroscopic debridement is not needed. If the course is not adequate after the 3rd debridement, graft and hardware removal should be considered. MRI may help in identifying the cause of persistent infection in those cases.**
- **We recommend the Gächter classification along with graft condition to select the optimal surgical treatment for ACL-R infections.**
- **Graft and hardware must be removed in the case of multiple debridement procedure failures and/or hardware loosening/graft insufficiency. We recommend that graft reimplantation be performed after 6 weeks in selected cases in cases of graft and hardware removal.**
- **Most common causative pathogens of infections after ACL-R are coagulase-negative staphylococci and S. aureus. Other pathogens include anaerobes, gram-negative rods, streptococci and enterococci.**
- **Empirical antibiotic treatment should be initiated immediately after joint aspiration if infection is suspected.**
- **Although clinical evidence is weak, based on theoretical considerations and extrapolation from other implant-associated infections, the use of biofilm-active antibiotics in infections with any grafts and fixation devices in place is advisable.**
- **We suggest a 1 week (up to 2 weeks) of intravenous treatment followed by oral treatment for another 4-5 weeks, preferably with bactericidal agents with good bioavailability and bone penetration as well as biofilm-activity if avascular tissue and fixation devices are in situ. The conditions for switching to oral treatment are a good clinical response with nearly normal CRP values.**
- **Functional scores might be impaired in patients with infection after ACL-R. Early diagnosis and treatment are crucial to preventing arthrofibrosis, stiffness and to preserve cartilage.**
- **In patients with an infection after ACL-R, the same rehabilitation protocols are recommended as for ACL-R to prevent arthrofibrosis. It is advisable to start as soon as the symptoms suggestive of infection are resolved. Of course, there should be adjustments based on the symptoms and pain experienced. Muscle strength and ROM should be the physical therapist's target.**

BIBLIOGRAPHY

1. Torres-Claramunt R, Gelber P, Pelfort X, Hinarejos P, Leal-Blanquet J, Pérez-Prieto D, et al. Managing septic arthritis after knee ligament reconstruction. *Int Orthop* 2016;40(3):607–614.

2. Pérez-Prieto D, Trampuz A, Torres-Claramunt R, Eugenia Portillo M, Puig-Verdié L, Monllau JC. Infections after Anterior Cruciate Ligament Reconstruction: Which Antibiotic after Arthroscopic Debridement? *J Knee Surg* 2017;30(4):309–313.

3. Williams RJ, Laurencin CT, Warren RF, Speciale AC, Brause BD, O'Brien S. Septic arthritis after arthroscopic anterior cruciate ligament reconstruction. Diagnosis and management. *Am J Sports Med* 1997;25(2):261–267.

4. Koh D, Tan SM, Tan AHC. Recurrent surgical site infection after anterior cruciate ligament reconstruction: A case report. *World J Orthop* 2019;10(6):255–261.

5. Mei-Dan O, Mann G, Steinbacher G, Ballester SJ, Cugat RB, Alvarez PD. Septic arthritis with Staphylococcus lugdunensis following arthroscopic ACL revision with BPTB allograft. *Knee Surg Sports Traumatol Arthrosc* 2008;16(1):15–18.

6. Ng SWL, Yee Han DL. Lessons learnt from an atypical mycobacterium infection post-anterior cruciate ligament reconstruction. *Clin Orthop Surg* 2015;7(1):135–139.

7. Burke WV, Zych GA. Fungal infection following replacement of the anterior cruciate ligament: a case report. *J Bone Joint Surg Am* 2002;84(3):449–453.

8. Wang C, Ao Y, Wang J, Hu Y, Cui G, Yu J. Septic arthritis after arthroscopic anterior cruciate ligament reconstruction: a retrospective analysis of incidence, presentation, treatment, and cause. *Arthroscopy* 2009;25(3):243–249.

9. Murphy MV, Du DT, Hua W, Cortez KJ, Butler MG, Davis RL, et al. Risk Factors for Surgical Site Infections Following Anterior Cruciate Ligament Reconstruction. *Infect Control Hosp Epidemiol* 2016;37(7):827–833.

10. Alomar AZ, Alfayez SM, Somily AM. Hamstring autografts are associated with a high rate of contamination in anterior cruciate ligament reconstruction. *Knee Surg Sports Traumatol Arthrosc* 2018;26(5):1357–1361.

11. Pérez-Prieto D, Portillo ME, Torres-Claramunt R, Pelfort X, Hinarejos P, Monllau JC. Contamination occurs during ACL graft harvesting and manipulation, but it can be easily eradicated. *Knee Surg Sports Traumatol Arthrosc* 2018;26(2):558–562.

12. Badran MA, Moemen DM. Hamstring graft bacterial contamination during anterior cruciate ligament reconstruction: clinical and microbiological study. *Int Orthop* 2016;40(9):1899–1903.

13. Nakayama H, Yagi M, Yoshiya S, Takesue Y. Micro-organism colonization and intraoperative contamination in patients undergoing arthroscopic anterior cruciate ligament reconstruction. *Arthroscopy* 2012;28(5):667–671.

14. Gavriilidis I, Pakos EE, Wipfler B, Benetos IS, Paessler HH. Intra-operative hamstring tendon graft contamination in anterior cruciate ligament reconstruction. *Knee Surg Sports Traumatol Arthrosc* 2009;17(9):1043–1047.

15. Stucken C, Garras DN, Shaner JL, Cohen SB. Infections in anterior cruciate ligament reconstruction. *Sports Health* 2013;5(6):553–557.

16. Armstrong RW, Bolding F, Joseph R. Septic arthritis following arthroscopy: clinical syndromes and analysis of risk factors. *Arthroscopy* 1992;8(2):213–223.

17. Armstrong RW, Bolding F. Septic arthritis after arthroscopy: the contributing roles of intraarticular steroids and environmental factors. *Am J Infect Control* 1994;22(1):16–18.

18. Cadet ER, Makhni EC, Mehran N, Schulz BM. Management of septic arthritis following anterior cruciate ligament reconstruction: a review of current practices and recommendations. *J Am Acad Orthop Surg* 2013;21(11):647–656.

19. Sonnery-Cottet B, Archbold P, Zayni R, Bortolletto J, Thaunat M, Prost T, et al. Prevalence of septic arthritis after anterior cruciate ligament reconstruction among professional athletes. *Am J Sports Med* 2011;39(11):2371–2376.

20. Bohu Y, Klouche S, Herman S, Pamphilis O de, Gerometta A, Lefevre N. Professional Athletes Are Not at a Higher Risk of Infections After Anterior Cruciate Ligament Reconstruction: Incidence of Septic Arthritis, Additional Costs, and Clinical Outcomes From the French Prospective Anterior Cruciate Ligament Study (FAST) Cohort. *Am J Sports Med* 2019;47(1):104–111.

21. Brophy RH, Wright RW, Huston LJ, Nwosu SK, MOON Knee Group, Spindler KP. Factors associated with infection following anterior cruciate ligament reconstruction. *J Bone Joint Surg Am* 2015;97(6):450–454.

22. Westermann R, Anthony CA, Duchman KR, Gao Y, Pugely AJ, Hettrich CM, et al. Infection following Anterior Cruciate Ligament Reconstruction: An Analysis of 6,389 Cases. *J Knee Surg* 2017;30(6):535–543.

23. Conen A, Borens O. 11.1 Septic arthritis. *cal* 17:18.

24. Cole BJ, Schumacher HR. Injectable corticosteroids in modern practice. *J Am Acad Orthop Surg* 2005;13(1):37–46.

25. Maletis GB, Inacio MCS, Reynolds S, Desmond JL, Maletis MM, Funahashi TT. Incidence of postoperative anterior cruciate ligament reconstruction infections: graft choice makes a difference. *Am J Sports Med* 2013;41(8):1780–1785.

26. Bansal A, Lamplot JD, VandenBerg J, Brophy RH. Meta-analysis of the Risk of Infections After Anterior Cruciate Ligament Reconstruction by Graft Type. *Am J Sports Med* 2018;46(6):1500–1508.

27. Schuster P, Schlumberger M, Mayer P, Raoulis VA, Oremek D, Eichinger M, et al. Lower incidence of post-operative septic arthritis following revision anterior cruciate ligament reconstruction with quadriceps tendon compared to hamstring tendons. *Knee Surg Sports Traumatol Arthrosc* 2020;

28. Pérez-Prieto D, Torres-Claramunt R, Gelber PE, Shehata TMA, Pelfort X, Monllau JC. Autograft soaking in vancomycin reduces the risk of infection after anterior cruciate ligament reconstruction. *Knee Surg Sports Traumatol Arthrosc* 2016;24(9):2724–2728.

29. Barker JU, Drakos MC, Maak TG, Warren RF, Williams RJ, Allen AA. Effect of graft selection on the incidence of postoperative infection in anterior cruciate ligament reconstruction. *Am J Sports Med* 2010;38(2):281–286.

30. Grassi A, Nitri M, Moulton SG, Marcheggiani Muccioli GM, Bondi A, Romagnoli M, et al. Does the type of graft affect the outcome of revision anterior cruciate ligament reconstruction? a meta-analysis of 32 studies. *Bone Joint J* 2017;99-B(6):714–723.

31. Pelfort X, Romero A, Brugués M, García A, Gil S, Marrón A. Reduction of periprosthetic Staphylococcus aureus infection by preoperative screening and decolonization of nasal carriers undergoing total knee arthroplasty. *Acta Orthop Traumatol Turc* 2019;

32. Weiser MC, Moucha CS. The Current State of Screening and Decolonization for the Prevention of Staphylococcus aureus Surgical Site Infection After Total Hip and Knee Arthroplasty. *J Bone Joint Surg Am* 2015;97(17):1449–1458.

33. Hadi H, Jabalameli M, Bagherifard A, Ghaznavi-Rad E, Behrouzi A, Joorabchi A, et al. Staphylococcus aureus Colonization in Patients Undergoing Total Hip or Knee Arthroplasty and Cost-effectiveness of Decolonization Programme. *Arch Bone Jt Surg* 2018;6(6):554–559.

34. Williams DM, Miller AO, Henry MW, Westrich GH, Ghomrawi HMK. Cost-Effectiveness of Staphylococcus aureus Decolonization Strategies in High-Risk Total Joint Arthroplasty Patients. *J Arthroplasty* 2017;32(9S):S91–S96.

35. Bratzler DW, Dellinger EP, Olsen KM, Perl TM, Auwaerter PG, Bolon MK, et al. Clinical practice guidelines for antimicrobial prophylaxis in surgery. *Surg Infect (Larchmt)* 2013;14(1):73–156.

36. Weber WP, Marti WR, Zwahlen M, Misteli H, Rosenthal R, Reck S, et al. The timing of surgical antimicrobial prophylaxis. *Ann Surg* 2008;247(6):918–926.

37. AlBuhairan B, Hind D, Hutchinson A. Antibiotic prophylaxis for wound infections in total joint arthroplasty: a systematic review. *J Bone Joint Surg Br* 2008;90(7):915–919.

38. Mauerhan DR, Nelson CL, Smith DL, Fitzgerald RH, Slama TG, Petty RW, et al. Prophylaxis against infection in total joint arthroplasty. One day of cefuroxime compared with three days of cefazolin. *J Bone Joint Surg Am* 1994;76(1):39–45.

39. Carney J, Heckmann N, Mayer EN, Alluri RK, Vangsness CT, Hatch Iii GF, et al. Should antibiotics be administered before arthroscopic knee surgery? A systematic review of the literature. *World J Orthop* 2018;9(11):262–270.

40. Information NC for B, Pike USNL of M 8600 R, MD B, Usa 20894. *Summary of a systematic review on surgical antibiotic prophylaxis prolongation*. Global Guidelines for the Prevention of Surgical Site Infection. World Health Organization, 2018. [cited 31 Mar 2020].

41. Stefánsdóttir A, Johansson A, Lidgren L, Wagner P, W-Dahl A. Bacterial colonization and resistance patterns in 133 patients undergoing a primary hip- or knee replacement in Southern Sweden. *Acta Orthop* 2013;84(1):87–91.

42. Vertullo CJ, Quick M, Jones A, Grayson JE. A surgical technique using presoaked vancomycin hamstring grafts to decrease the risk of infection after anterior cruciate ligament reconstruction. *Arthroscopy* 2012;28(3):337–342.

43. Grayson JE, Grant GD, Dukie S, Vertullo CJ. The in vitro elution characteristics of vancomycin from tendons. *Clin Orthop Relat Res* 2011;469(10):2948–2952.

44. Baron JE, Shamrock AG, Cates WT, Cates RA, An Q, Wolf BR, et al. Graft Preparation with Intraoperative Vancomycin Decreases Infection After ACL Reconstruction: A Review of 1,640 Cases. *J Bone Joint Surg Am* 2019;

45. Figueroa D, Figueroa F, Calvo R, Lopez M, Goñi I. Presoaking of Hamstring Autografts in Vancomycin Decreases the Occurrence of Infection Following Primary Anterior Cruciate Ligament Reconstruction. *Orthop J Sports Med* 2019;7(9):2325967119871038.

46. Offerhaus C, Balke M, Hente J, Gehling M, Blendl S, Höher J. Vancomycin pre-soaking of the graft reduces postoperative infection rate without increasing risk of graft failure and arthrofibrosis in ACL reconstruction. *Knee Surg Sports Traumatol Arthrosc* 2019;27(9):3014–3021.

47. Phegan M, Grayson JE, Vertullo CJ. No infections in 1300 anterior cruciate ligament reconstructions with vancomycin pre-soaking of hamstring grafts. *Knee Surg Sports Traumatol Arthrosc* 2016;24(9):2729–2735.

48. Naendrup J-H, Marche B, Sa D de, Koenen P, Otchwemah R, Wafaisade A, et al. Vancomycin-soaking of the graft reduces the incidence of septic arthritis following ACL reconstruction: results of a systematic review and meta-analysis. *Knee Surg Sports Traumatol Arthrosc* 2019;

49. Bohu Y, Klouche S, Sezer HB, Herman S, Grimaud O, Gerometta A, et al. Vancomycin-soaked autografts during ACL reconstruction reduce the risk of post-operative infection without affecting return to sport or knee function. *Knee Surg Sports Traumatol Arthrosc* 2020;

50. Yazdi H, Yousof Gomrokchi A, Nazarian A, Lechtig A, Hanna P, Ghorbanhoseini M. The Effect of Gentamycin in the Irrigating Solution to Prevent Joint Infection after Anterior Cruciate Ligament (ACL) Reconstruction. *Arch Bone Jt Surg* 2019;7(1):67–74.

51. Musso AD, McCormack RG. *Infection after ACL reconstruction: what happens when cultures are negative? Clinical journal of sport medicine : official journal of the Canadian Academy of Sport Medicine*. 2005.

52. Mouzopoulos G, Fotopoulos VC, Tzurbakis M. Septic knee arthritis following ACL reconstruction: a systematic review. *Knee Surg Sports Traumatol Arthrosc* 2009;17(9):1033–1042.

53. Bauer T, Boisrenoult P, Jenny JY. Post-arthroscopy septic arthritis: Current data and practical recommendations. *Orthopaedics & traumatology, surgery & research : OTSR* 2015;101(8 Suppl).

54. Gobbi A, Karnatzikos G, Chaurasia S, Abhishek M, Bulgherhoni E, Lane J. Postoperative Infection After Anterior Cruciate Ligament Reconstruction. *Sports Health* 2016;8(2):187–189.

55. Torres-Claramunt R, Pelfort X, Erquicia J, Gil-Gonzalez S, Gelber PE, Puig L, et al. Knee joint infection after ACL reconstruction: prevalence, management and functional outcomes. *Knee Surg Sports Traumatol Arthrosc* 2013;21(12):2844–2849.

56. Roerdink RL, Huijbregts HJTA, Lieshout AWT, Dietvorst M, Zwaard BC. The difference between native septic arthritis and prosthetic joint infections: A review of literature. *Journal of Orthopaedic Surgery* 2019;27(2).

57. Izakovicova P, Borens O, Trampuz A. Periprosthetic joint infection: current concepts and outlook. *EFORT Open Rev* 2019;4(7):482–494.

58. Zimmerli W, Trampuz A, Ochsner PE. Prosthetic-joint infections. *N Engl J Med* 2004;351(16):1645–1654.

59. Margaretten ME, Kohlwes J, Moore D, Bent S. Does this adult patient have septic arthritis? *Jama* 2007;297(13):1478–1488.

60. Wang C, Lee YH, Siebold R. Recommendations for the management of septic arthritis after ACL reconstruction. *Knee Surg Sports Traumatol Arthrosc* 2014;22(9):2136–2144.

61. Renz N, Mudrovcic S, Perka C, Trampuz A. Orthopedic implant-associated infections caused by Cutibacterium spp. - A remaining diagnostic challenge. *PLoS ONE* 2018;13(8):e0202639.

62. Baillet A, Trocme C, Romand X, Nguyen CMV, Courtier A, Toussaint B, et al. Calprotectin discriminates septic arthritis from pseudogout and rheumatoid arthritis. *Rheumatology* Oxford, England, 2019;58(9):1644–1648.

63. Margheritini F, Camillieri G, Mancini L, Mariani PP. C-reactive protein and erythrocyte sedimentation rate changes following arthroscopically assisted anterior cruciate ligament reconstruction. *Knee Surg Sports Traumatol Arthrosc* 2001;9(6):343–345.

64. Ruiz-Iban MA, Diaz Heredia J, Martinez Val IC, Alonso Guemes S, Cuellar Gutierrez R, Sastre Solsona S. Evolution of C-reactive protein values in the first month after anterior cruciate ligament reconstruction: reference values. *Knee Surg Sports Traumatol Arthrosc* 2015;23(3):763–769.

65. Papakonstantinou O, Chung CB, Chanchairujira K, Resnick DL. Complications of anterior cruciate ligament reconstruction: MR imaging. *Eur Radiol* 2003;13(5):1106–1117.

66. Judd D, Bottoni C, Kim D, Burke M, Hooker S. Infections following arthroscopic anterior cruciate ligament reconstruction. *Arthroscopy* 2006;22(4):375–384.

67. Hughes JG, Vetter EA, Patel R, Schleck CD, Harmsen S, Turgeant LT, et al. 3rd: Culture with BACTEC Peds Plus/F bottle compared with conventional methods for detection of bacteria in synovial fluid. *Journal of clinical microbiology* 2001;39(12):4468–4471.

68. Louie JS, Liebling MR. The polymerase chain reaction in infectious and post-infectious arthritis. A review. *Rheumatic diseases clinics of North America* 1998;24(2):227–236.

69. Yermak K, Karbysheva S, Perka C, Trampuz A, Renz N. Performance of synovial fluid D-lactate for the diagnosis of periprosthetic joint infection: A prospective observational study. *J Infect* 2019;79(2):123–129.

70. Gratacos J, Vila J, Moya F, Marcos MA, Collado A, Sanmarti R, et al. D-lactic acid in synovial fluid. A rapid diagnostic test for bacterial synovitis. *The Journal of rheumatology* 1995;22(8):1504–1508.

71. Kortekangas P, Peltola O, Toivanen A, Aro HT. Synovial-fluid D-lactic acid in bacterial and other acute joint effusions. *Scandinavian journal of rheumatology* 1994;23(4):203–205.

72. Wouthuyzen-Bakker M, Ploegmakers JJW, Ottink K, Kampinga GA, Wagenmakers-Huizenga L, Jutte PC, et al. Synovial Calprotectin: An Inexpensive Biomarker to Exclude a Chronic Prosthetic Joint Infection. *The Journal of arthroplasty* 2018;33(4):1149–1153.

73. Salari P, Grassi M, Cinti B, Onori N, Gigante A. Synovial Fluid Calprotectin for the Preoperative Diagnosis of Chronic Periprosthetic Joint Infection. *The Journal of arthroplasty* 2020;35(2):534–537.

74. Wouthuyzen-Bakker M, Ploegmakers JJW, Kampinga GA, Wagenmakers-Huizenga L, Jutte PC, Muller Kobold AC. Synovial calprotectin: a potential biomarker to exclude a prosthetic joint infection. *The bone & joint journal* 2017;99-b(5):660-665.

75. Krenn V, Morawietz L, Burmester GR, Haupl T. Synovialitis score: histopathological grading system for chronic rheumatic and non-rheumatic synovialitis. *Zeitschrift fur Rheumatologie* 2005;64(5):334–342.

76. Illgner U, Krenn V, Osada N, Bause L. Histopathology and microbiology of joint infections: extension of diagnostic safety in patients with chronic polyarthritis. *Zeitschrift fur Rheumatologie* 2013;72(7):709–713.

77. Dudareva M, Barrett L, Figtree M, Scarborough M, Watanabe M, Newnham R, et al. Sonication versus Tissue Sampling for Diagnosis of Prosthetic Joint and Other Orthopedic Device-Related Infections. *Journal of clinical microbiology* 2018;56(12).

78. Oliva A, Pavone P, D'Abramo A, Iannetta M, Mastroianni CM, Vullo V. Role of Sonication in the Microbiological Diagnosis of Implant-Associated Infections: Beyond the Orthopedic Prosthesis. *Adv Exp Med Biol* 2016;897:85–102.

79. Trampuz A, Piper KE, Jacobson MJ, Hanssen AD, Unni KK, Osmon DR, et al. Sonication of removed hip and knee prostheses for diagnosis of infection. *The New England journal of medicine* 2007;357(7):654–663.

80. Indelli PF, Dillingham M, Fanton G, Schurman DJ. Septic arthritis in postoperative anterior cruciate ligament reconstruction. *Clin Orthop Relat Res* 2002;(398):182–188.

81. Van Tongel A, Stuyck J, Bellemans J, Vandenneucker H. Septic arthritis after arthroscopic anterior cruciate ligament reconstruction: a retrospective analysis of incidence, management and outcome. *Am J Sports Med* 2007;35(7):1059–1063.

82. Manadan AM, Block JA. Daily needle aspiration versus surgical lavage for the treatment of bacterial septic arthritis in adults. *Am J Ther* 2004;11(5):412–415.

83. Ravindran V, Logan I, Bourke BE. Medical vs surgical treatment for the native joint in septic arthritis: a 6-year, single UK academic centre experience. *Rheumatology (Oxford)* 2009;48(10):1320–1322.

84. Smith RL, Schurman DJ, Kajiyama G, Mell M, Gilkerson E. The effect of antibiotics on the destruction of cartilage in experimental infectious arthritis. *J Bone Joint Surg Am* 1987;69(7):1063–1068.

85. Schollin-Borg M, Michaëlsson K, Rahme H. Presentation, outcome, and cause of septic arthritis after anterior cruciate ligament reconstruction: a case control study. *Arthroscopy* 2003;19(9):941–947.

86. Stutz G, Kuster MS, Kleinstück F, Gächter A. Arthroscopic management of septic arthritis: stages of infection and results. *Knee Surg Sports Traumatol Arthrosc* 2000;8(5):270–274.

87. Stutz G, Gächter A. [Diagnosis and stage-related therapy of joint infections]. *Unfallchirurg* 2001;104(8):682–686.

88. Peres LR, Marchitto RO, Pereira GS, Yoshino FS, Castro Fernandes M de, Matsumoto MH. Arthrotomy versus arthroscopy in the treatment of septic arthritis of the knee in adults: a randomized clinical trial. *Knee Surg Sports Traumatol Arthrosc* 2016;24(10):3155–3162.

89. Calvo R, Figueroa D, Anastasiadis Z, Vaisman A, Olid A, Gili F, et al. Septic arthritis in ACL reconstruction surgery with hamstring autografts. Eleven years of experience. *Knee* 2014;21(3):717–720.

90. Studahl M, Bergman B, Kälebo P, Lindberg J. Septic arthritis of the knee: a 10-year review and long-term follow-up using a new scoring system. *Scand J Infect Dis* 1994;26(1):85–93.

91. Hantes ME, Raoulis VA, Doxariotis N, Drakos A, Karachalios T, Malizos KN. Management of septic arthritis after arthroscopic anterior cruciate ligament reconstruction using a standard surgical protocol. *Knee* 2017;24(3):588–593.

92. Pogorzelski J, Themessl A, Achtnich A, Fritz EM, Wörtler K, Imhoff AB, et al. Septic Arthritis After Anterior Cruciate Ligament Reconstruction: How Important Is Graft Salvage? *Am J Sports Med* 2018;46(10):2376–2383.

93. Saper M, Stephenson K, Heisey M. Arthroscopic irrigation and debridement in the treatment of septic arthritis after anterior cruciate ligament reconstruction. *Arthroscopy* 2014;30(6):747–754.

94. Binnet MS, Başarir K. Risk and outcome of infection after different arthroscopic anterior cruciate ligament reconstruction techniques. *Arthroscopy* 2007;23(8):862–868.

95. McAllister DR, Parker RD, Cooper AE, Recht MP, Abate J. Outcomes of postoperative septic arthritis after anterior cruciate ligament reconstruction. *Am J Sports Med* 1999;27(5):562–570.

96. Abdel-Aziz A, Radwan YA, Rizk A. Multiple arthroscopic debridement and graft retention in septic knee arthritis after ACL reconstruction: a prospective case-control study. *Int Orthop* 2014;38(1):73–82.

97. Balato G, Di Donato S, Ascione T, D'Addona A, Smeraglia F, Di Vico G, et al. Knee Septic Arthritis after Arthroscopy: Incidence, Risk Factors, Functional Outcome, and Infection Eradication Rate. *Joints* 2017;05(02):107–113.

98. Gellert M, Hardt S, Koder K, Renz N, Perka C, Trampuz A. Biofilm-active antibiotic treatment improved the outcome of knee periprosthetic joint infection: Results from a 6-year prospective cohort. *International journal of antimicrobial agents* 2020(105904).

99. John AK, Baldoni D, Haschke M, Rentsch K, Schaerli P, Zimmerli W, et al. Efficacy of daptomycin in implant-associated infection due to methicillin-resistant Staphylococcus aureus: importance of combination with rifampin. *Antimicrob Agents Chemother* 2009;53(7):2719–2724.

100. Zimmerli W, Widmer AF, Blatter M, Frei R, Ochsner PE. *Role of rifampin for treatment of orthopedic implant-related staphylococcal infections: a randomized controlled trial*. Foreign-Body Infection (FBI) Study Group, 1998.

101. Widmer AF, Gaechter A, Ochsner PE, Zimmerli W. *Antimicrobial treatment of orthopedic implant-related infections with rifampin combinations. Clinical infectious diseases : an official publication of the Infectious Diseases Society of America*. 1992.

102. OC EH, EF B, BD L, JE E-P, RR R, IG S, et al. Efficacy and safety of rifampin containing regimen for staphylococcal prosthetic joint infections treated with debridement and retention. *Eur J Clin Microbiol Infect Dis* 2010;29(8):961–967.

103. U FT, S C, B B, W Z, A T. Role of rifampin against Propionibacterium acnes biofilm in vitro and in an experimental foreign-body infection model. *Antimicrob Agents Chemother* 2012;56(4):1885–1891.

104. Conen A, Borens O. Septic Arthritis. In: Kates SL, Davos BO, eds. *Principles of Orthopedic Infection Management*. edn. Switzerland: AO Publishing, 2016:213–226.

105. Iversen K, Ihlemann N, Gill SU, Madsen T, Elming H, Jensen KT, et al. Partial Oral versus Intravenous Antibiotic Treatment of Endocarditis. *The New England journal of medicine* 2019;380(5):415–424.

106. Li HK, Rombach I, Zambellas R, Walker AS, McNally MA, Atkins BL, et al. Oral versus Intravenous Antibiotics for Bone and Joint Infection. *The New England journal of medicine* 2019;380(5):425–436.

107. Margaryan D, Conen A, Renz N, Feihl S, Pérez-Prieto D, Trampuz A. Pro-Implant Foundation Pocket Guide to Diagnosis & Treatment of Septic Arthritis (Native Joints & after Ligament Repair. *Version* 2020;1.

108. Makhni EC, Steinhaus ME, Mehran N, Schulz BS, Ahmad CS. Functional Outcome and Graft Retention in Patients With Septic Arthritis After Anterior Cruciate Ligament Reconstruction: A Systematic Review. *Arthroscopy* 2015;31(7):1392–1401.

109. Boström Windhamre H, Mikkelsen C, Forssblad M, Willberg L. Postoperative septic arthritis after anterior cruciate ligament reconstruction: does it affect the outcome? A retrospective controlled study. *Arthroscopy* 2014;30(9):1100–1109.

110. Trueta J, Barnes JM. The Rationale of Complete Immobilization in Treatment of Infected Wounds. *Br Med J* 1940;2(4149):46–48.

111. Trueta J. Treatment of acute osteomyelitis. *Lancet* 1948;2(6515):68.

112. Schuster P, Schulz M, Immendoerfer M, Mayer P, Schlumberger M, Richter J. Septic Arthritis After Arthroscopic Anterior Cruciate Ligament Reconstruction: Evaluation of an Arthroscopic Graft-Retaining Treatment Protocol. *Am J Sports Med* 2015;43(12):3005–3012.
