# Supplementary material for: Guideline for management of septic arthritis in native joints (SANJO)
Source: J Bone Jt Infect. 2023 Jan 12;8(1):29–37. doi: 10.5194/jbji-8-29-2023 (PMC9901514; doi:10.5194/jbji-8-29-2023)

EBJIS guideline Workgroup 8: Special considerations with TB SANJO

Authors:
Leonard Marais
Head of department: Orthopaedics / School of Clinical Medicine
Nelson R Mandela School of Medicine, Durban, South Africa

Recommendations for tuberculosis arthritis

Question 1: When should the diagnosis of arthritis caused by Mycobacterium tuberculosis be suspected?

- We suggest considering the diagnosis of tuberculosis (TB) arthritis in any patient with **symptoms/signs of arthritis**, such as joint pain, swelling, effusion, restricted movement and/or deformity involving one or more joints, **with a subacute or chronic course** (weeks to months or even years) (A-II).
- Additionally, we recommend **increasing the suspicion of TB arthritis** in:
  - patients living or that previously lived in highly TB endemic areas (A-II)
  - patients with a prior history of untreated (or wrongly treated) TB disease, simultaneous TB in other site, and/or chest radiography with signs of old TB (A-II)
  - patients with monoarthritis with an indolent course, even if systemic symptoms such as low-grade fever, night sweats or weight loss are absent, and there are not evident risk factors for TB (migration from TB endemic area, comorbid illness, immunosuppression) (A-III)
  - any suspected infectious arthritis with conventional culture negative and not responding to empirical treatment of pyogenic arthritis (A-III).
  - Patients X-ray features of peri-articular osteopenia, subchondral erosions, peri-articular cysts or fractures, narrowing of the joint space and/or bony destruction or sclerosis.

Question 2: What tests should be performed in patients with suspicion of native joint infection caused by M. tuberculosis?

- Routine **infection markers** are indicated. An Erythrocyte sedimentation rate (ESR) > 40mm/h should raise suspicion.
- **Plain radiographs** of the involved and contralateral joint are recommended in all patients at baseline (A-II). Although X-ray findings are often non-specific, it can rule out other diagnoses and serve as baseline image for evaluating any future joint impairment. Additional imaging studies are not commonly necessary (D-III).
- We suggest performing **ultrasound** for detecting effusion if it is not evident by physical examination and for guiding joint aspiration in deep joints such as the hip (A-II).

- **Magnetic resonance imaging (MRI)** is suggested if advanced imaging is required, such as in patients with suspicion of coexistent periarticular osteomyelitis or to better evaluate the extent of disease (B-III). It may also be useful when TB arthritis is suspected, and plain radiographs are normal (in early stages of disease) (C-III).
- We recommend sending a sample of **synovial fluid**, with as much fluid as possible, in a sterile container for acid-fast bacilli (AFB) stain, mycobacterial culture, and nucleic acid amplification test (NAAT) (A-II).
- A **synovial biopsy** specimen for microbiological studies (AFB stain, mycobacterial culture and NAAT), as well as histopathological analysis, is recommended in patients with suspected TB arthritis and negative AFB stain and *M. tuberculosis* NAAT (A-II).

Question 3: **What is the recommended antimicrobial therapy for arthritis due to *M. tuberculosis* and its optimal duration?**

- We recommend an initial medical therapy for TB of a combination of 4 drugs including rifampin, isoniazid, ethambutol and pyrazinamide for 2 months; ethambutol may be discontinued if susceptibility to the other 3 drugs is demonstrated (A-II).
- After the induction 2-month period, patients with drug-susceptible TB should continue with isoniazid and rifampin (A-II). Treatment of patients with drug-resistant TB should be guided by an infectious diseases expert in the field (A-III).
- The optimal duration of antituberculous therapy for arthritis TB is uncertain. We suggest 6 to 12-month duration regimens, which should be supervised by an infectious diseases expert (A-III).

Question 4: **What are the special considerations related to the surgical management of native joint infection caused by *M. Tuberculosis?***

- We suggest treating early cases of TB arthritis with medical therapy alone (B-III).
- We suggest considering surgical intervention with debridement and synovectomy in the active phase of TB arthritis for patients with large abscesses, significant devitalized bone or showing inadequate response to medical management (A-II).

- Patients with substantial joint destruction, ankylosis, deformity, significant loss of function or chronic pain after TB arthritis may benefit from operative management with excisional arthroplasty or arthrodesis (A-III). Total joint arthroplasty may be considered in patients with hip or knee involvement, but the optimal time to perform the surgery after the TB treatment it is not clear (B-III).

Rationale

Tuberculosis (TB) primary infection is usually acquired through inhalation of *Mycobacterium tuberculosis*. During primary infection, hematogenous dissemination may occur and lead to seeding of bacilli in many sites, including bones and joints, although it is generally contained by cell-mediated immunity. Manifestations of bone and joint TB are more common during reactivation of latent bacilli, often many years after the initial infection, but it may also occur during primary infection.[1] The latter is more common in young patients, in highly endemic areas, while the former occurs mainly in adults outside of these areas.[2,3] The most frequent form of skeletal TB is spondylitis (Pott disease) followed by arthritis.[3,4,5]
TB arthritis usually presents as a chronic granulomatous monoarthritis of insidious onset, that slowly progresses to joint destruction in the absence of adequate treatment.[1,6] The most commonly affected sites are the weightbearing joints namely the hip, knee, foot and ankle, followed by the shoulder, elbow and wrist; however, any joint may be involved.[5] Up to 10% of patients will present with polyarticular affectation. Clinical manifestations include pain, swelling, and/or impairment of joint function that evolves over weeks to months or even years.[17] Warmth and erythema are typically absent. Systemic symptoms of low-grade fever, night sweats or weight loss are often lacking (with prevalence ranging from 13 to 50% in different series).[72] Previous history of TB is rarely present (less than 25% of cases)[89] and pulmonary active TB is uncommon (less than 50% of cases, although reported rates vary greatly). [1593] Patients are frequently diagnosed late in the course of the disease (duration of symptoms/signs before diagnosis ranges 8 to 25 months in different series)[579] and they may present with joint contracture or evidence of joint destruction including local deformity, restricted range of motion and even a draining sinus.

Risk for TB arthritis is greater in people living or that previously lived in regions with high TB endemicity, although they currently reside in no-endemic areas. Other risk factors include an older age (mainly in industrialized countries), chronic underlying diseases (as diabetes mellitus, chronic renal disease or malignancy), alcoholism, low socioeconomics and impaired immunity (mainly the use of corticosteroids and other immunomodulatory medications).[157]

The main challenge in the diagnosis of joint TB is to consider this diagnosis, due to its low incidence (especially in industrialized countries), usual absence of simultaneous lung disease and constitutional TB symptoms, and its indolent course with scarce inflammatory signs. The gold standard for diagnosis is the identification or isolation of *M. tuberculosis* by microscopy (acid-fast bacilli [AFB] smear microscopy), culture, or nucleic acid amplification test (NAAT).[110] Mycobacterial susceptibility study should be performed on culture isolates. In the absence of extraarticular TB, diagnosis of TB arthritis requires microbiological study of synovial biopsy, which is more sensitive (90% of positive cultures).[111]

Moreover, histopathology of biopsy often shows characteristic granulomas, and occasionally yields AFB on staining.[1] The culture has tradionally been considered the most sensitive test, but its main drawback is the long time (generally several weeks) it takes. NAATs can provide a rapid diagnosis; the Xpert MTB/RIF assay is an automated NAAT that can detect simultaneously *M. tuberculosis* and rifampin resistance, and has demonstrated good diagnostic accuracy for bone and joint TB.[121314] Recent data suggest than the more recent Xpert Ultra may increase the yield.[15] Although a positive NAAT confirms the diagnosis, a negative result cannot rule out TB. Few data are available on the sensitivity and specificity of NAAT in the diagnosis of culture-negative TB.[1]

Low haemoglobin level and normal white blood cell counts are seen in most of patients.[3] Elevated erythrocyte sedimentation rate (ESR) and C-reactive protein (CRP) levels are frequent but lack of specificity.[3] Synovial fluid is commonly turbid, with leucocyte counts between 10,000 and 20,000 cells/ml and a preponderance of polymorphonuclears (PMN). [16] Other noninvasive tests assess prior exposure to TB antigens, such as the tuberculin skin test (TST) - also denominated purified protein derivative (PPD) test- and IFN-gamma release assays (IGRAs). While both IGRA and TST provide evidence for infection with TB, they cannot distinguish active from

latent TB. [11017] The absence of positive TST or IGRAs should not exclude a diagnosis of TB; up to 50% of patients with disseminated tuberculosis have negative TSTs.[1]

Radiographic imaging can show soft tissue swelling and effusion in early stages. Ultrasonography is more useful to confirm the presence of an effusion or synovial thickening and to guide synovial fluid (or abscess) aspiration.[18] Later on, findings include juxtaarticular osteopenia, peripherally located osseous erosions and progressive articular destruction with narrowing of the joint space (Phemister triad).[1918] The end stage of TB arthritis is characterized by severe joint destruction and eventually sclerosis and fibrous ankylosis.[20] Magnetic resonance imaging (MRI) is useful to demonstrate early changes, as well as to characterize the extent of disease, including soft tissue and bone adjacent affectation.[20]

Treatment of TB arthritis is largely based on antimicrobial therapy, and it may be managed with medical therapy alone, although surgical intervention may also be warranted.[121219] In early cases, antituberculous therapy can result in complete resolution, without residual joint disease.[2] This supports the importance of a high level of suspicion of joint TB and an early, aggressive approach to diagnosis.

According to the EUA Centers for Diseases Control and Prevention guidelines, a 6- to 9-month regimen containing rifampin is recommended for treatment of bone and joint TB.[21] Initial medical therapy for drug-susceptible TB consists of a combination of 4 drugs including rifampin, isoniazid, ethambutol and pyrazinamide for 2 months (ethambutol may be discontinued if susceptibility to the other 3 drugs is demonstrated).[121] After this induction period, patients with drug-susceptible TB should continue with isoniazid and rifampin for a minimum of 4 to 7 more months of therapy.[21] However, the optimal duration of antituberculous therapy for bone and joint TB is uncertain.[12] Most of the information comes from old studies on spinal TB, with relevant drawbacks.[222324] Some experts tend to favor 9 to 12-month duration regimens, particularly in patients who present with a significant burden of disease or a high net state of immunosuppression.[12] Antituberculous treatment should be supervised by an expert in the field, particularly in case of multidrug-resistant TB.

In terms of the orthopaedic management, the initial approach is typically non-operative. This should focus on the prevention of deformity (through bracing or splinting) and the maintenance of range of motion of the involved join (though

physiotherapy). In the lower extremity, limiting weightbearing should be considered in case with significant bone or joint destruction to prevent progressive deformities. While the backbone of treatment of joint TB remains antituberculous therapy, surgery is necessary in some cases, although its role in not always clear.[19] Surgical intervention in the active phase of TB arthritis is usually considered for patients with large abscesses, significant devitalized bone and/or showing inadequate response to medical management.[1912526] However, some authors have suggested that surgical debridement and synovectomy may expedite healing and limit joint damage.[2728293031] There is however little evidence in the literature to support these statements, since clinical trials or even sound observational studies with control groups are lacking. Further studies would be needed to determine if arthroscopic or open debridement is superior to medical management alone.

Although total joint arthroplasty is typically not performed during the active phase of TB arthritis, in may be unavoidable in certain instances and in these cases arthroplasty followed by long-term antimycobacterial therapy may be used with some success.[1] Thus, while active infection was previously considered to be contraindication for arthroplasty surgery, with long intervals of at least 10 years being advocated, there have been a number of reports of total hip arthroplasty (THA) in cases with active TB infection.[1931] A systematic review on the outcome of single-stage THA in 65 patients with active hip TB found only one case of reactivation in a patient who was non-compliant with treatment after a follow-up of 53 months (2-9 years).[32] However, the evidence for total knee arthroplasty (TKA) in patients with active knee TB remains limited.[33] Most authors emphasize the importance of a thorough debridement when doing arthroplasty in the setting of active infection. Patients with quiescent infection and substantial joint destruction, fibrous ankylosis with significant loss of function and/or chronic pain after TB arthritis may also benefit from operative management.[191] Excisional arthroplasty, joint replacement surgery or arthrodesis may be considered to treat these sequalae of TB arthritis.[12919] For the hip and knee, joint replacement appears to be superior in terms of the functional outcome.[34353637] Optimally, total joint arthroplasty after TB arthritis should be deferred until patients show no evidence of recurrent disease after completion of therapy given the potential for reactivation disease after arthroplasty.[1] However, the optimal time interval between the treatment of joint TB and the arthroplasty surgery

is not known. In most cases, patients undergoing joint replacement received pre and post-operative prolonged TB therapy.

Disclosures

LCM has nothing to disclose

References

1. Hogan JI, Hurtado RM, Nelson SB. Mycobacterial Musculoskeletal Infections. Infect Dis Clin North Am. 2017;31(2):369–82.
2. Pigrau-Serrallach C, Rodríguez-Pardo D. Bone and joint tuberculosis. Eur Spine J. 2013;22(SUPPL.4):556–66.
3. Johansen IS, Nielsen SL, Hove M, Kehrer M, Shakar S, Wøyen AVT, et al. Characteristics and Clinical Outcome of Bone and Joint Tuberculosis from 1994 to 2011: A Retrospective Register-based Study in Denmark. Clin Infect Dis. 2015;61(4):554–62.
4. Mateo L, Manzano JR, Olivé A, Manterola JM, Pérez R, Tena X, et al. Tuberculosis osteoarticular: Estudio de 53 casos. Med Clin (Barc) [Internet]. 2007;129(13):506–9. Available from: http://dx.doi.org/10.1157/13111371
5. Qian Y, Han Q, Liu W, Yuan WE, Fan C. Characteristics and management of bone and joint tuberculosis in native and migrant population in Shanghai during 2011 to 2015 11 Medical and Health Sciences 1103 Clinical Sciences. BMC Infect Dis. 2018;18(1):1–10.
6. Berney S, Goldstein M, Bishko F. Clinical and diagnostic features of tuberculous arthritis. Am J Med. 1972;53(1):36–42.
7. Huang TY, Wu TS, Yang CC, Chiang PC, Yu KH, Lee MH. Tuberculous arthritis - A fourteen-year experience at a tertiary teaching hospital in Taiwan. J Microbiol Immunol Infect. 2007;40(6):493–9.
8. Mariconda M, Cozzolino A, Attingenti P, Cozzolino F, Milano C. Osteoarticular tuberculosis in a developed country. J Infect. 2007;54:357–80.
9. Ali R, Jalil A, Qureshi A. Extra spinal osteoarticular tuberculosis: A case series of 66 patients from a tertiary care hospital in Karachi. J Pak Med Assoc. 2012;62(12):1344–8.
10. Lewinsohn DM, Leonard MK, LoBue PA, Cohn DL, Daley CL, Desmond E, et al. Official American Thoracic Society/Infectious Diseases Society of

America/Centers for Disease Control and Prevention Clinical Practice Guidelines: Diagnosis of Tuberculosis in Adults and Children. Clin Infect Dis [Internet]. 2017 Jan 15;64(2):111–5. Available from: https://academic.oup.com/cid/article/64/2/111/2811357

11. Ohl CA, Forster D. Infectious Arthritis of Native Joints. In: Bennet J, Dolin R, Blaser MJ, editors. Mandell, Douglas, and Bennett's Principles and Practice of Infectious Diseases. Ninth Edit. Philadelphia: Elsevier Inc.; 2020. p. 1400–17.
12. Shen Y, Yu G, Zhong F, Kong X. Diagnostic accuracy of the Xpert MTB/RIF assay for bone and joint tuberculosis: A meta-analysis. Cheungpasitporn W, editor. PLoS One [Internet]. 2019 Aug 22;14(8):e0221427. Available from: https://dx.plos.org/10.1371/journal.pone.0221427
13. Li Y, Jia W, Lei G, Zhao D, Wang G, Qin S. Diagnostic efficiency of Xpert MTB/RIF assay for osteoarticular tuberculosis in patients with inflammatory arthritis in China. Hasnain SE, editor. PLoS One [Internet]. 2018 Jun 1;13(6):e0198600. Available from: https://dx.plos.org/10.1371/journal.pone.0198600
14. Gu Y, Wang G, Dong W, Li Y, Ma Y, Shang Y, et al. Xpert MTB/RIF and GenoType MTBDRplus assays for the rapid diagnosis of bone and joint tuberculosis. Int J Infect Dis [Internet]. 2015 Jul;36:27–30. Available from: http://dx.doi.org/10.1016/j.ijid.2015.05.014
15. Sun Q, Wang S, Dong W, Jiang G, Huo F, Ma Y, et al. Diagnostic value of Xpert MTB/RIF Ultra for osteoarticular tuberculosis. J Infect [Internet]. 2019;79(2):153–8. Available from: https://doi.org/10.1016/j.jinf.2019.06.006
16. Garrido G, Gomez-Reino JJ, Fernandez-Dapica P, Palenque E, Prieto S. A review of peripheral tuberculous arthritis. Semin Arthritis Rheum. 1988;18(2):142–9.
17. Jia H, Pan L, Qin S, Liu F, Du F, Lan T, et al. Evaluation of interferon-γ release assay in the diagnosis of osteoarticular tuberculosis. Diagn Microbiol Infect Dis [Internet]. 2013 Jul;76(3):309–13. Available from: http://dx.doi.org/10.1016/j.diagmicrobio.2013.03.030
18. Griffith JF, Kumta SM, Leung PC, Cheng JCY, Chow LTC, Metreweli C. Imaging of musculoskeletal tuberculosis: A new look at an old disease. Clin Orthop Relat Res. 2002;(398):32–9.
19. Tuli SM. General Principles of Osteoarticular Tuberculosis. Clin Orthop Relat

Res [Internet]. 2002 May 31;398(398):11–9. Available from: https://www.taylorfrancis.com/books/9781000094367/chapters/10.4324/9780429317903-11

20. De Vuyst D, Vanhoenacker F, Gielen J, Bernaerts A, De Schepper AM. Imaging features of musculoskeletal tuberculosis. Eur Radiol. 2003;13(8):1809–19.

21. Nahid P, Dorman SE, Alipanah N, Barry PM, Brozek JL, Cattamanchi A, et al. Official American Thoracic Society/Centers for Disease Control and Prevention/Infectious Diseases Society of America Clinical Practice Guidelines: Treatment of Drug-Susceptible Tuberculosis. Clin Infect Dis. 2016;63(7):e147–95.

22. A controlled trial of six-month and nine-month regimens of chemotherapy in patients undergoing radical surgery for tuberculosis of the spine in Hong Kong. Tubercle [Internet]. 1986 Dec;67(4):243–59. Available from: https://linkinghub.elsevier.com/retrieve/pii/0041387986900140

23. Controlled trial of short-course regimens of chemotherapy in the ambulatory treatment of spinal tuberculosis. Results at three years of a study in Korea. Twelfth report of the Medical Research Council Working Party on Tuberculosis of the Spine. J Bone Joint Surg Br [Internet]. 1993 Mar;75(2):240–8. Available from: https://online.boneandjoint.org.uk/doi/10.1302/0301-620X.75B2.8444944

24. Five-year assessment of controlled trials of short-course chemotherapy regimens of 6, 9 or 18 months' duration for spinal tuberculosis in patients ambulatory from the start or undergoing radical surgery. Fourteenth report of the Medical Research Council W. Int Orthop [Internet]. 1999;23(2):73–81. Available from: http://www.ncbi.nlm.nih.gov/pubmed/10422019

25. Lesić AR, Pesut DP, Marković-Denić L, Maksimović J, Cobeljić G, Milosević I, et al. The challenge of osteo-articular tuberculosis in the twenty-first century: a 15-year population-based study. Int J Tuberc Lung Dis [Internet]. 2010 Sep;14(9):1181–6. Available from: http://www.ncbi.nlm.nih.gov/pubmed/20819266

26. Gursu S, Yildirim T, Ucpinar H, Sofu H, Camurcu Y, Sahin V, et al. Long-term follow-up results of foot and ankle tuberculosis in Turkey. J Foot Ankle Surg. 2014;53(5):557–61.

27. Vohra R, Kang HS, Dogra S, Saggar RR, Sharma R. Tuberculous

osteomyelitis. J Bone Jt Surg [Internet]. 1997 Jul 1;79(4):562–6. Available from: http://www.bjj.boneandjoint.org.uk/cgi/doi/10.1302/0301-620X.79B4.7618

28. Gardam M, Lim S. Mycobacterial Osteomyelitis and Arthritis. Infect Dis Clin North Am [Internet]. 2005 Dec;19(4):819–30. Available from: https://linkinghub.elsevier.com/retrieve/pii/S0891552005000735

29. Dhillon MS, Nagi ON. Tuberculosis of the foot and ankle. Clin Orthop Relat Res. 2002;(398):107–13.

30. Samuel S, Boopalan PRJVC, Alexander M, Ismavel R, Varghese VD, Mathai T. Tuberculosis of and around the ankle. J Foot Ankle Surg [Internet]. 2011;50(4):466–72. Available from: http://dx.doi.org/10.1053/j.jfas.2011.04.002

31. Duan X, Yang L. Arthroscopic management for early-stage tuberculosis of the ankle. J Orthop Surg Res. 2019;14(1):1–8.

32. Kim S-J, Postigo R, Koo S, Kim JH. Total hip replacement for patients with active tuberculosis of the hip. Bone Joint J [Internet]. 2013 May;95-B(5):578–82. Available from: https://online.boneandjoint.org.uk/doi/10.1302/0301-620X.95B5.31047

33. Habaxi K-K, Wang L, Miao X-G, Alimasi WQ-K, Zhao X-B, Su J-G, et al. Total knee arthroplasty treatment of active tuberculosis of the knee: a review of 10 cases. Eur Rev Med Pharmacol Sci [Internet]. 2014;18(23):3587–92. Available from: http://www.ncbi.nlm.nih.gov/pubmed/25535127

34. Liu CS, Liu FZ, Wang XY, Yao Y, Qiao L, Fu J, et al. Comparison of total curative effect between total hip arthroplasty and hip arthrodesis in treating coxotuberculosis. Eur Rev Med Pharmacol Sci. 2018;22(1):90–5.

35. Kumar V, Garg B, Malhotra R. Total hip replacement for arthritis following tuberculosis of hip. World J Orthop. 2015;6(8):636–40.

36. Zeng M, Xie J, Wang L, Hu Y. Total knee arthroplasty in advanced tuberculous arthritis of the knee. Int Orthop [Internet]. 2016;40(7):1433–9. Available from: http://dx.doi.org/10.1007/s00264-015-3050-x

37. Zeng M, Hu Y, Leng Y, Xie J, Wang L, Li M, et al. Cementless total hip arthroplasty in advanced tuberculosis of the hip. Int Orthop. 2015;39(11):2103–7.

Appendices

1) Search strategy

<u>MESH terms:</u>

Joint AND Tuberculosis

<u>Pubmed Search String:</u>

(JOINT) AND TUBERCULOSIS AND ((Clinical Study[ptyp] OR Clinical Trial[ptyp] OR Comparative Study[ptyp] OR Review[ptyp] OR systematic[sb] OR Classical Article[ptyp] OR Case Reports[ptyp]) AND ("1970/01/01"[PDat] : "2019/12/31"[PDat]) AND Humans[Mesh] AND English[lang] AND medline[sb] AND adult[MeSH])

<u>Included articles</u>:

- Human
- English
- Article
- Search since 1970

<u>Excluded:</u>

- Case reports
- Narrative reviews
- Periprosthetic joint TB
- Paediatric patients <18yr
- Rheumatoid Arthritis
- Spine and SI joint
- Tempo-mandibular joint
- TB osteomyelitis
- TB Tenosynovitis
- Basic science (non-clinical) research
- Non-Tuberculous Mycobacteria
- Dactylitis
- Symphysis pubis

Search Results for Question 3 – Surgical Management: Additional search criteria applied during abstract screening

- Describes surgical management of patients and outcomes
- Studies done in last 20 years (since 1999)
- Studies with 9 or more cases

Level of Evidence and Strength of Recommendation:

According to GRADE system. [Guyatt Gordon H, Oxman Andrew D, Vist Gunn E, Kunz Regina, Falck-Ytter Yngve, Alonso-CoelloPablo et al. GRADE: an emerging consensus on rating quality of evidence and strength of recommendations. *BMJ* 2008; 336 :924].

Fig.1 Flow diagram showing selection of included studies.

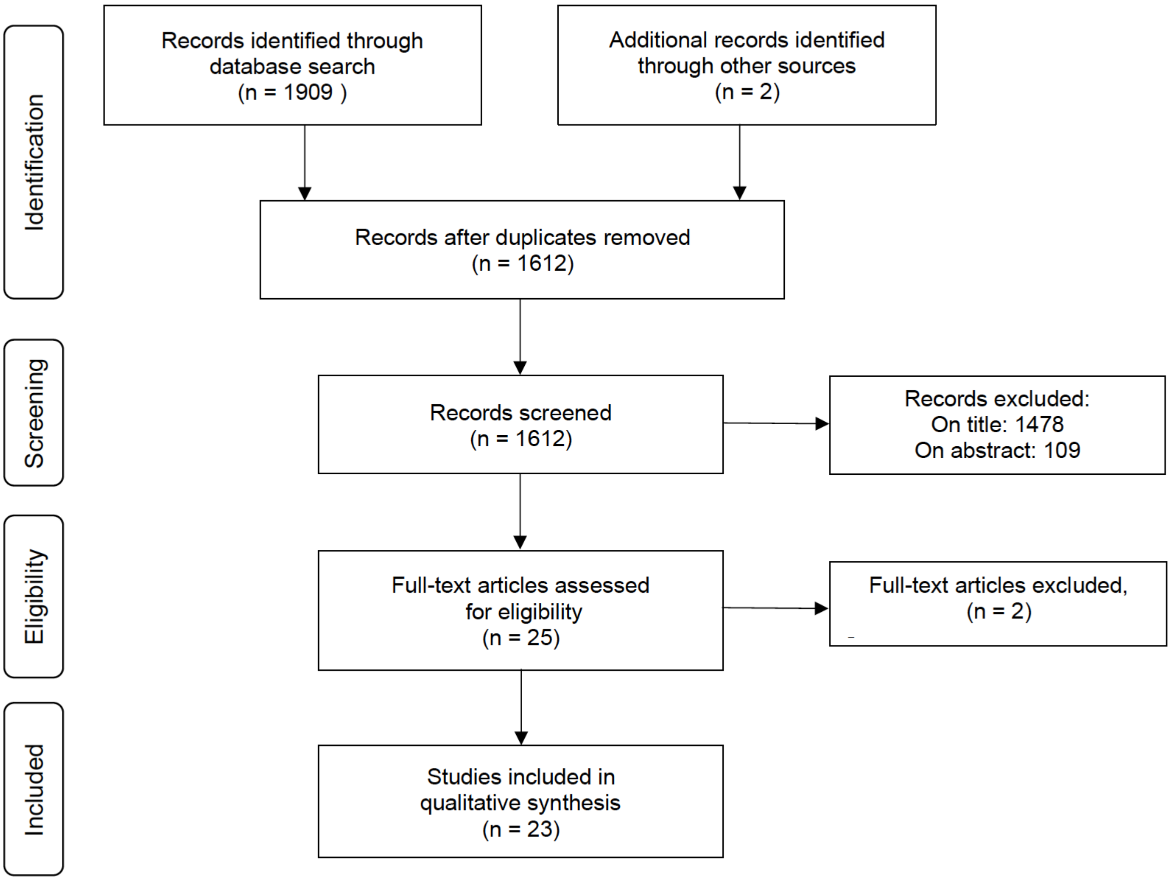

Supplement: The supplement related to this article is available online at: https://doi.org/10.5194/jbji-8-29-2023-supplement. [file jbji-8-29-supplement.zip › Workgroup report 8, TB SANJO.pdf]
