# Supplementary material for: Guideline for management of septic arthritis in native joints (SANJO)
Source: J Bone Jt Infect. 2023 Jan 12;8(1):29–37. doi: 10.5194/jbji-8-29-2023 (PMC9901514; doi:10.5194/jbji-8-29-2023)
Supplement: The supplement related to this article is available online at: https://doi.org/10.5194/jbji-8-29-2023-supplement. [file jbji-8-29-supplement.zip › Workgroup report 5, mobilization after SANJO.pdf]

EBJIS guideline Workgroup 5: Joint Mobilization

Author
Parham Sendi, MD, FIDSA
University Hospital of Basel, University of Basel
AND
Institute for Infectious Diseases, University of Bern, Bern, Switzerland

Background and aim

Joint function after treatment of septic arthritis is impaired in up to 30% of patients [1]. Multiple variables can influence the final functional outcome. In this document, the effect, optimal starting time, and modalities of joint mobilization are evaluated.

Patients with septic arthritis are usually managed in the inpatient hospital setting to begin with and require an interprofessional team of caregivers. Joint mobilization starts when the infection is clinically under control.

The choice of rest versus motion has always presented a dilemma in the management of musculoskeletal injury and disease [2]. Treatment of injured joints has included immobilization because of the intuition that injury demands a period of rest for healing to occur and the commonsense reasoning that moving a painful part would produce more pain. The forefathers of orthopedics also strongly advocated treatment with enforced, uninterrupted, and prolonged rest. The end results were often atrophy, stiffness, and contractions. Prolonged immobilization following surgical intervention is a common cause of knee motion limitation [3]. Paulos et al. [3] reported that infrapatellar contracture syndrome is a recognized cause of posttraumatic knee morbidity.

Encouraging results with early motion led to studies of the biology of repair under the influence of motion [4-7]. However, these were mostly experimental animal and basic studies. Salter et al. [2, 6, 7] and O'Driscoll et al. [4] have demonstrated the beneficial effects of continuous passive motion not only on healing of articular cartilage defects, but also on fluid exchange; they have also justified its use for septic arthritis. These studies showed that early active or passive non-weight-bearing mobilization improves the nutrition of synovial cartilage and resists the weakening of surrounding muscles [8].

What are the strategies of joint mobilization?

The goal is to reduce pain and restore and maintain physiological joint function. After reviewing the literature and involving a multidisciplinary expert panel, Chabaud et al. [9] proposed a consensus protocol after septic knee arthritis. The authors suggested the following three phases:

- early mobilization to recover range of motion
- muscular strengthening first unloaded without any resistance
- gradual reloading with a walking stick and finishing with functional work

Naylor et al. [10] categorized the rehabilitation periods after septic arthritis into three time periods, namely:

- the early rehabilitation phase (0 to 6 weeks)
- the middle rehabilitation phase (6 to 14 weeks)
- the late rehabilitation phase (15 to 30 weeks)

Each of these phases has goals with respect to body function and structure, activity of the physiotherapist, and participation of the patient. These phases should be adapted according to the anatomic site of infection and the patient's perspective for rehabilitation (e.g., daily practice activity, professional sport activity).

Summary of recommendations and level of evidence

When should joint mobilization be started?
After an infection is under control, motion should begin slowly but deliberately. Motion should not begin before the drains have been removed and the drain holes closed. This approach may decrease the incidence of chronic drainage or fistula formation [11, 12]. In line with the individual's healing dynamic after an infection, there is no uniform and precisely defined time point for starting mobilization. Reported starting time points after active symptoms subside have ranged from 24 to 48 hours [13] up to 5 days [13, 14]. Katz et al. [15] started passive motion of the involved joint in 14 children (mean 3 years [2 weeks to 9 years]) on the first day after drainage.

How many mobilization cycles/day should there be and when should weight bearing be started?
There is no evidence on the number of mobilization cycles per day. The daily implementation of joint mobilization depends on numerous factors, including the patient's physical strength and ambition, pain management, institutional possibilities (e.g., mechanical devices for continuous passive motion, physiotherapist schedule, number of qualified caregivers), and costs.

Katz et al. [15] gradually increased motion as much as the patient could tolerate in their study with children. Passive motion was continued until the child resumed active motion spontaneously. Older children, in addition, were encouraged to move their affected limb actively as much as they could. Weight bearing was encouraged as soon as possible.

Sreenivas et al. [16] reported knee mobilization after 2 weeks and weight bearing at 6 weeks in their series of 26 cases of acute hematogenous septic arthritis in adults. Diefenbeck et al. [17] recommend light weight bearing (i.e. 10 kg) of the lower extremities within the first 3 weeks and a gradual weight increase over next 3 weeks.

How should the joint be rested between mobilization cycles?
No studies are available on joint rest between mobilization cycles. Recommendations include allowing the joint to be in its functional position and positioning the joint to allow passive range of motion activities (e.g., continuous passive motion). Cooling, analgesics, and anti-inflammatory therapy contribute to pain relief and detumescence and may be beneficial during rest periods. In addition, they may allow early and continuous mobilization and alleviate synovitis [17]. However, these treatment practices have not yet been systematically evaluated.

Does regional anesthesia for pain relief during mobilization have any drawbacks?
In patients with native joint septic arthritis, this question has not been systematically evaluated or investigated within a study setting. The majority of data stem from a setting other than septic arthritis, namely, from an optimal postoperative analgesic regimen following total knee replacement. Randomized trials and systematic reviews have concluded that local infiltration of anesthetics can provide improved postoperative pain relief [18-20]. Thus, when joint resistance to mobilization is met, manipulation techniques may also be used under anesthesia followed by the resumption of the activity initiated up to that point, with an emphasis on increasing the joint's amplitude of motion. Drawbacks of regional anesthesia for pain relief are not specific to septic arthritis and include adverse effects and allergies, similar to all drugs.

Bagry et al. [21] investigated the effect of a continuous peripheral nerve block on the inflammatory response in knee arthroplasty in 12 patients. After receiving spinal anesthesia, they were randomly allocated to either patient-controlled analgesia with morphine (n = 6) or a combination of continuous lumbar plexus and sciatic nerve blocks with ropivacaine 0.2% for 48 hours. Interestingly, continuous lumbar plexus and sciatic nerve blocks with ropivacaine contributed to the attenuation of the postoperative inflammatory response. It is unclear whether or not this should be taken into consideration in early relapse cases or in infections that are not yet controlled.

Conclusions
In septic arthritis of the native joint, there is not enough evidence to recommend one mobilization strategy over another. Previous uncontrolled observational studies have shown that prolonged immobilization following surgical intervention leads to limitation of knee motion, atrophy, stiffness, and contractions. Conversely, animal and basic studies have demonstrated the beneficial effects of continuous passive motion. These results have been implemented in clinical practice [22].

Mobilization is recommended to be started *after* drains have been removed and surgical wounds closed. Most experts recommend mobilization when infection is controlled (i.e., within 24 to 48 hours up to the first 5 days) and then gradually increasing weight bearing within the first 6 weeks.
Further recommendations on joint mobilization cycles per day, joint rest between cycles, starting weight bearing, amount of weight when bearing, and pain management in septic arthritis derive from institutional and personal experience, as well textbooks, and hence, differ between centers. In addition, the patient's joint function prior to infection and her or his perspective regarding joint use must be considered. Studies are needed to evaluate and validate joint mobilization concepts and to ensure results are comparable between institutions. These studies are feasible, in particular when considering the number of assessment scores available to measure joint function [10, 15, 16, 23].

Literature review

Material and methods
Literature search strategy: We used the electronic database MEDLINE (Medical Literature Analysis and Retrieval System Online) through the PubMed interface and the Google Books search engine. Keywords and Medical Subject Heading (MeSH) terms used were as follows: septic arthritis, native joint arthritis, infectious arthritis, rehabilitation, physical therapy, joint mobilization, recovery.

Literature review with evidence grading and highlight on bias
Level IV evidence

Disclosures
Parham Sendi: none

References

1. Sharp JT, Lidsky MD, Duffy J, Duncan MW. Infectious arthritis. Arch Intern Med **1979**; 139(10): 1125-30.
2. Salter RB. History of rest and motion and the scientific basis for early continuous passive motion. Hand Clin **1996**; 12(1): 1-11.
3. Paulos LE, Rosenberg TD, Drawbert J, Manning J, Abbott P. Infrapatellar contracture syndrome. An unrecognized cause of knee stiffness with patella entrapment and patella infera. Am J Sports Med **1987**; 15(4): 331-41.
4. O'Driscoll SW, Kumar A, Salter RB. The effect of continuous passive motion on the clearance of a hemarthrosis from a synovial joint. An experimental investigation in the rabbit. Clin Orthop Relat Res **1983**; (176): 305-11.
5. Mooney V, Stills M. Continuous passive motion with joint fractures and infections. Orthop Clin North Am **1987**; 18(1): 1-9.
6. Salter RB, Bell RS, Keeley FW. The protective effect of continuous passive motion in living articular cartilage in acute septic arthritis: an experimental investigation in the rabbit. Clin Orthop Relat Res **1981**; (159): 223-47.
7. Salter RB, Simmonds DF, Malcolm BW, Rumble EJ, MacMichael D, Clements ND. The biological effect of continuous passive motion on the healing of full-thickness defects in articular cartilage. An experimental investigation in the rabbit. J Bone Joint Surg Am **1980**; 62(8): 1232-51.
8. Salter RB. The biologic concept of continuous passive motion of synovial joints. The first 18 years of basic research and its clinical application. Clin Orthop Relat Res **1989**; (242): 12-25.
9. Chabaud A, Tetard M, Descamps S, et al. P070 Therapeutic algorithm for nativeseptic arthritis rehabilitation of the knee. Ann Phys Rehabil Med **2017**; 60(September 2017): e32.
10. Naylor AR, Briggs MS, Kegelmeyer DK, Kloos AD. Rehabilitation and functional outcomes after extensive surgical debridement of a knee infected by fusobacterium necrophorum: a case report. Int J Sports Phys Ther **2013**; 8(3): 311-27.

11. Ballard A, Burkhalter WE, Mayfield GW, Dehne E, Brown PW. The functional treatment of pyogenic arthritis of the adult knee. J Bone Joint Surg Am **1975**; 57(8): 1119-23.
12. Schurman DJ, Smith RL. Surgical approach to the management of septic arthritis. Orthop Rev **1987**; 16(4): 241-5.
13. Donatto KC. Orthopedic management of pyogenic arthritis. Compr Ther **1999**; 25(8-10): 411-7.
14. Simank HG, Wadi B, Bernd L. [Joint empyema]. Orthopade **2004**; 33(3): 327-31.
15. Katz K, Goldberg I, Yosipovitch Z. Early mobilization in septic arthritis. 14 children followed for 2 years. Acta Orthop Scand **1990**; 61(2): 161-2.
16. Sreenivas T, Nataraj AR, Menon J. Acute hematogenous septic arthritis of the knee in adults. Eur J Orthop Surg Traumatol **2013**; 23(7): 803-7.
17. Diefenbeck M, Abitzsch D, Hofmann GO. [Joint infections. Known facts and new trends]. Unfallchirurg **2012**; 115(6): 489-95.
18. Gibbs DM, Green TP, Esler CN. The local infiltration of analgesia following total knee replacement: a review of current literature. J Bone Joint Surg Br **2012**; 94(9): 1154-9.
19. Stein BE, Srikumaran U, Tan EW, Freehill MT, Wilckens JH. Lower-extremity peripheral nerve blocks in the perioperative pain management of orthopaedic patients: AAOS exhibit selection. J Bone Joint Surg Am **2012**; 94(22): e167.
20. Thomas K, Barrett B, Tupper R, Dacenko-Grawe L, Holm K. Pain management after total knee arthroplasty: a case–control study of continuous nerve block therapy. Orthop Nurs **2014**; 33(5): 268-76.
21. Bagry H, de la Cuadra Fontaine JC, Asenjo JF, Bracco D, Carli F. Effect of a continuous peripheral nerve block on the inflammatory response in knee arthroplasty. Reg Anesth Pain Med **2008**; 33(1): 17-23.
22. McCarthy MR, O'Donoghue PC, Yates CK, Yates-McCarthy JL. The clinical use of continuous passive motion in physical therapy. J Orthop Sports Phys Ther **1992**; 15(3): 132-40.
23. Mexim A, Apostu D, Cosam D. A literature review: physicaltherapy of the hip after septic arthritis. Balneo Research Journal **2018**; 9(3): 299 302.
