# Supplementary material for: Guideline for management of septic arthritis in native joints (SANJO)
Source: J Bone Jt Infect. 2023 Jan 12;8(1):29–37. doi: 10.5194/jbji-8-29-2023 (PMC9901514; doi:10.5194/jbji-8-29-2023)
Supplement: The supplement related to this article is available online at: https://doi.org/10.5194/jbji-8-29-2023-supplement. [file jbji-8-29-supplement.zip › jbji-8-29-2023-supplement-title-page.pdf]

Supplement of J. Bone Joint Infect., 8, 29–37, 2023
https://doi.org/10.5194/jbji-8-29-2023-supplement
© Author(s) 2023. CC BY 4.0 License.

Supplement of

Guideline for management of septic arthritis in native joints (SANJO)

Christen Ravn et al.

Correspondence to: Christen Ravn (christen.ravn@rm.dk) and Jeroen Neyt (jeroen.neyt@uzgent.be)

- jbji-8-29-2023-supplement-title-page.pdf
- Workgroup report 1, diagnosis of SANJO.pdf
- Workgroup report 2, microbiological methods of SANJO.pdf
- Workgroup report 3, initial surgical treatment of SANJO.pdf
- Workgroup report 4, empirical antibiotic treatment of SANJO.pdf
- Workgroup report 5, mobilization after SANJO.pdf
- Workgroup report 6, outcome evaluation.pdf
- Workgroup report 7, ACL-R infection.pdf
- Workgroup report 8, TB SANJO.pdf
- Workgroup report 9, Pediatric SANJO.pdf

The copyright of individual parts of the supplement might differ from the article licence.
