# Supplementary material for: Guideline for management of septic arthritis in native joints (SANJO)
Source: J Bone Jt Infect. 2023 Jan 12;8(1):29–37. doi: 10.5194/jbji-8-29-2023 (PMC9901514; doi:10.5194/jbji-8-29-2023)
Supplement: The supplement related to this article is available online at: https://doi.org/10.5194/jbji-8-29-2023-supplement. [file jbji-8-29-supplement.zip › Workgroup report 6, outcome evaluation.pdf]

EBJIS guideline Workgroup 6: Outcome evaluation

Authors:
Jeroen GV Neyt[1], Guillem Bori[2], Ireneusz Babiak[3], Tomislav Madjarevic[4], Len Marais[5], Aditya Menon[6], Dirk Jan F. Moojen[7], Marta Sabater-Martos[8], Imre Sallai[9], Sharma Sunil[10], Javier Martínez[11]

1 Department of Orthopaedic and Trauma Surgery, University Hospitals of Ghent, Ghent, Belgium
2 Cirurgia Ortopèdica i Traumatologia.Hospital Clínic. Universitat de Barcelona, Spain
3Medical University Warschaw, Poland
4 University Hospital for Orthopaedic Surgery Lovran, Croatia University Of Rijeka, Croatia
5 Dept Orthopaedics University of KwaZulu-Natal, Durban South Africa
6 Department of Orthopaedics,P. D. Hinduja Hospital and MRC, Mahim, Mumbai, India
7 Department of Orthopaedic and Trauma Surgery, JointResearch, OLVG Hospital, Amsterdam, The Netherlands
8 Department of Orthopaedic and Trauma Surgery, Hospital Clínic de Barcelona, Spain
9 Budapest Orthopaedic Department (Semmelweis University), Hungary
10 Department of Orthopaedic Surgery, Brighton and Sussex University Hospitals NHS, UK
11 Servicio de Cirugía Ortopédica y Traumatología. Unidad Patología Séptica Ap. Locomotor. CSUR Infecciones Osteoarticulares Resistentes.Hospital Clínico Universitario " Virgen de la Arrixaca", Murcia. España

What are the most important signs of treatment failure during treatment for septic arthritis in native joints (SANJO)?
Based on the limited evidence available in the literature and with expert-based input, we recommend looking out for the following signs indicating treatment failure:

A Clinical signs: persistent pain and/or local signs of inflammation (including presence of purulent discharge) and/or systemic signs of infection and/or deteriorating joint function
B Serology: CRP not decreasing or rather increasing (WBC count)
C Synovial fluid at re-aspiration: elevated WBC count and percentage polymorphonuclear leucocytes, persistently positive microbial cultures

The clinician should primarily monitor the clinical, serological, and synovial signs of treatment failure. In case of prolonged treatment failure, imaging modalities may be able to demonstrate adjacent tissue damage.

Which key elements should be addressed for you when evaluating the treatment outcome of native joints after septic arthritis (SANJO)?
Based on the limited evidence available in the literature and with expert-based input, we recommend taking into account at least the following when evaluating treatment outcome:

1 parameters related to remaining joint function
2 the need for further surgery in the short, middle long and long term
3 eradication of infection to safely proceed to joint replacement for severe joint damage
4 increased mortality rates

Addendum / Supplemental information

Treatment Failure and Evaluation Outcome

Septic arthritis of a native joint should be seen as an acute septic condition with potentially devastating sequelae(1) and for that reason it requires immediate medical attention and prompt surgical treatment. Timing appears to be of essence in light of the inevitable buildup of bioburden but despite adhering to this principle deployed, remedial actions do not necessarily lead to a short and a long-term successful outcome. Defining the latter is one thing, defining what should be considered treatment failure is another. Unfortunately, there is sparse literature and a lack of high-quality evidence to outline this framework of definitions. In order to facilitate some attempt in the runup towards this goal, two basic questions should assist to evaluate treatment failure in non-responders and treatment outcomes. Subsequently, recommendations might follow.

1. ***Question 1: What are the most important signs of treatment failure during treatment for septic arthritis in native joints?***

Question 1.1: How to define treatment failure for an acute or a chronic infection nonresponding to standard first-line treatment with antibiotics and an invasive procedure or surgery?

*For this purpose, a large internet database was thoroughly searched for during the two decades i.e., from **2000 to 2021 (March)** with papers and abstracts mainly in English. Subsequently, a manual analysis of references of eligible articles was performed.*

Terms of interest/keywords: *Treatment failure, definition, native septic arthritis.*

Objective: *To determine whether a general definition to define treatment failure after the first medical and surgical treatment of native septic arthritis does exist.*

Results:

Few studies have addressed failure of treatment of native joint septic arthritis (NJSA) and definitions do vary. In the bibliographic review, just seven papers were found defining failure. They are as follows:

- *In a paper from New Zealand published in 2020 (coding based retrospective study on large (302 episodes) and small (250 episodes) native joints) treatment failure was defined as: any death within 90 days of index admission; relapse; reinfection; amputation, excision arthroplasty, or arthrodesis of an involved joint for ongoing infection; or readmission to hospital for ongoing NJSA. Relapse was defined as readmission for NJSA of the previously infected joint, with the same organism isolated or negative culture. Reinfection was defined as NJSA of the same joint caused by a different organism. (1)*
- *Treatment failure in a paper from Spain in 2015 (single center retrospective study with 186 patients) was defined as death, admission to the intensive care unit, the need for surgery after 72 h of initial treatment or readmission to the hospital for the same reason (2).*
- *In a French retrospective cohort study published in 2014 on methicillin sensitive Staphylococcus Aureus bone and joint infections treatment failure in sixty-six patients included i) persisting infection under appropriate antimicrobial therapy; and/or ii) relapse after antimicrobial therapy disruption (3).*
- *In a North American study (2015) with 128 adult patients and 132 native joints, any of the following signs or symptoms -developing during the postoperative period- were considered a recurrence of infection and thus failure of a single surgical debridement: persistent purulent discharge, from a drain or incisional site(s), increasing pain, decreasing range of motion, persistent fevers, or persistent elevation of serologic inflammatory markers (4).*
- *In a Korean single center study (2020) with 97 patients, postoperative recurrent infection of the adult native shoulder was confirmed by the following: recurrent clinical manifestations including aggravated pain and fever. Ominous laboratory evidence in the patient’s blood test included an elevated WBC count, ESR, and CRP level. In aspirated joint fluid, a WBC count of greater than 50,000 cells/mL would be suggestive for a recurrent infection (5).*

- *Failure was defined after medical treatment and/or surgical treatment in a paper on native hip (25) and knee (72) joint septic arthritis from France (2021). Medical treatment failure was defined as the need for surgery despite adequate antibiotic treatment for a period of 7 days. Surgical treatment failure was defined as the need for a second surgical intervention after a similar time lapse. Patients for whom surgery was delayed after one or two needle aspirations were included in the medical group, unless they had surgery during the first 7 days. In that case, they were included in the surgical group. Surgery required after 7 days was then considered a treatment failure. Also, death or amputation was considered a treatment failure (6).*
- *In a Spanish paper (2020) on Staphylococcus native joint arthritis in 29 patients, treatment failure was defined as any situation in which the objective of successfully treating the infection was not met, including death related to the infection, relapse or persistence of the infection after four weeks of treatment (7).*

- *In conclusion this literature review appears to harness case series studies only. Further, the definition of treatment failure for native septic arthritis is not universally accepted, let alone standardized. In aforementioned papers, authors have attempted to partially define treatment failure. Additional research is needed to standardize the definition of failure and outline concepts of persistent lack of source control.*

References:

1. *McBride S, Mowbray J, Caughey W, Wong E, Luey C, Siddiqui A, Alexander Z, Playle V, Askelund T, Hopkins C, Quek N, Ross K, Orec R, Mistry D, Coomarasamy C, Holland D. Epidemiology, Management, and Outcomes of Large and Small Native Joint Septic Arthritis in Adults. Clin Infect Dis. 2020 Jan 2;70(2):271-279. doi: 10.1093/cid/ciz265. PMID: 30941403.*
2. *Maneiro JR, Souto A, Cervantes EC, Mera A, Carmona L, Gomez-Reino JJ. Predictors of treatment failure and mortality in native septic arthritis. Clin Rheumatol. 2015 Nov;34(11):1961-7. doi: 10.1007/s10067-014-2844-3. Epub 2014 Dec 13. PMID: 25501634.*
3. *Valour F, Bouaziz A, Karsenty J, Ader F, Lustig S, Laurent F, Chidiac C, Ferry T; Lyon BJI study group. Determinants of methicillin-susceptible Staphylococcus aureus native bone and joint infection treatment failure: a retrospective cohort study. BMC Infect Dis. 2014 Aug 16;14:443. doi: 10.1186/1471-2334-14-443. PMID: 25128919; PMCID: PMC4147168.*
4. *Hunter JG, Gross JM, Dahl JD, Amsdell SL, Gorczyca JT. Risk factors for failure of a single surgical debridement in adults with acute septic arthritis. J Bone Joint Surg Am. 2015 Apr 1;97(7):558-64. doi: 10.2106/JBJS.N.00593. PMID: 25834080; PMCID: PMC6948783.*
5. *Joo YB, Lee WY, Shin HD, Kim KC, Kim YK. Risk factors for failure of eradicating infection in a single arthroscopic surgical procedure for septic arthritis of the adult native shoulder with a focus on the volume of irrigation. J Shoulder Elbow Surg. 2020 Mar;29(3):497-501. doi: 10.1016/j.jse.2019.07.014. Epub 2019 Sep 18. PMID: 31542323.*
6. *Mabille C, El Samad Y, Joseph C, Brunschweiler B, Goeb V, Grados F, Lanoix JP. Medical versus surgical treatment in native hip and knee septic arthritis. Infect Dis Now. 2021 Mar;51(2):164-169. doi: 10.1016/j.medmal.2020.04.019. Epub 2020 May 5. PMID: 32387296.*
7. *Muñoz-Gallego I, Mancheño M, Pérez-Montarelo D, Viedma E, Chaves F, Lora-Tamayo J. Staphylococcus aureus native arthritis over 10 years. Med Mal Infect. 2020 May;50(3):257-262. doi: 10.1016/j.medmal.2020.01.012. Epub 2020 Feb 11. PMID: 32057526.*

GRADE:

STUDY	*YEAR*	*TYPE OF STUDY*	*EVIDENCE QUALITY*
McBride S (1)	*2020*	*Case series*	⊕⊖⊖⊖ *Very Low*
Maneiro JR (2)	*2015*	*Case series*	⊕⊖⊖⊖ *Very Low*
Valour F(3)	*2014*	*Case series*	⊕⊖⊖⊖ *Very Low*
Hunter JG (4)	*2015*	*Case series*	⊕⊖⊖⊖ *Very Low*
Joo Y-B (5)	*2020*	*Retrospective case-control*	⊕⊖⊖⊖ *Very Low*
Mabille C (6)	*2021*	*Case series*	⊕⊖⊖⊖ *Very Low*
Muñoz-Gallego I (7)	*2020*	*Case series*	⊕⊖⊖⊖ *Very Low*

2. ***Question 2: Which key elements should be addressed for you when evaluating the treatment outcome of native joints after septic arthritis (SANJO)?***

Results:

A literature search on treatment outcome produced the following. Bauer et al (1) in France reported in a retrospective cohort study on fifty-three cases of NJSA (31 knees and 22 hips) treated by arthroplasty. The final results were assessed in terms of functional outcome (on PMA functional score for hips and IKS score for knees) and successful eradication of infection. Two-stage arthroplasty was successful in 26 of the 30 cases of evolutive septic arthritis (87%), while the 1-stage procedure was successful in 22 of the 23 cases of quiescent septic arthritis (95%) (NS). Functional results were very good. No significant difference in functional outcome or successful eradication of infection was found between the 1- and 2-stage procedures. No significant difference in final outcome in terms of infection eradication was found between knees and hips. No clinical, microbiological, or treatment-related criteria emerged as risk factors for septic failure.
Sultan et al (2) reported in the USA in a multicenter retrospective cohort study on the risk and associated risk factors of a periprosthetic joint infection in patients with a history of treated same-joint native joint septic arthritis. The final cohort included 62 patients who had a mean follow-up of 4.4 years (range, 3 months–17 years) from the time of TJA. A total of 21 patients (34%) had less than 2 years of follow up, including six (10%) mortalities. In total, eight patients (13%) died during the study period, none of which were due to PJI. In patients with a history of treated same-joint native septic arthritis, the proportion of PJI was five of 62 patients (8%). Patients with history of native joint infections are at higher risk of PJI, especially smokers. A minimum interval of 2 years from the time of resolving native joint septic arthritis to TJA appears to be a safer option. Medical optimization of comorbidities that may confer additional risk, such as diabetes, becomes exceptionally important in these patients.
Maneiro et al (3) in Spain evaluated in a retrospective single-center study with 186 patients' treatment failure for NJSA as primary outcome. Secondary outcomes included mortality, complications, endocarditis, bacteremia, hospital readmission and the duration of the hospital stay. The median age was 64 years, and the percentage of male patients was 68.9%. Predictors for treatment failure were a Staphylococcus aureus infection, endocarditis, and the involvement of joints difficult to access with needle drainage. Predictors for increased mortality were age, the leucocyte count at baseline, bacteremia, diabetes mellitus and chronic renal failure. According to the authors, the predictors for treatment failure were joint-related factors and systemic complications, whereas conditions related to the host's immune competence, such as age and comorbidities predict increased mortality.
Huang et al (4) investigated in a national cohort study the incidence, site-specific mortality, and prognostic factors of native septic arthritis (SA) in Taiwan. A total of 31 491 patients were identified as having SA, the most common site of infection being the knee (50.1%), followed by the hip (14.4%), other sites (26.8%), the shoulder (5.5%) and multiple sites (1.2%). The 30-day, 90-day and 1-year mortality rates were 4.3, 8.6 and 16.4% respectively. Predictors for mortality were hip infection, shoulder infection, multiple-site infection, being male, age ≥65 years old and comorbidities.
Abram et al (5) reported in a retrospective cohort study in patients who received arthroscopic knee washout for septic arthritis in England to determine the risk of mortality and adverse joint outcomes following septic arthritis of the native knee. A total of 12 132 patients were included (mean age 56·6 years of whom 4307 (36%) were female. Secondary septic arthritis diagnosis versus primary diagnosis was associated with an increased odds ratio for mortality. With at least 1 year follow-up, at one year 15 had had an arthrodesis, 46 patients had an amputation, and 152 patients had been scheduled for an arthroplasty. Within 15 years, 159 of 1816 patients had received an arthroplasty, corresponding to an annual risk of arthroplasty that was about six times that of the general population.

This literature search on treatment outcome produced a few papers highlighting the three most important parameters to assess outcome after treatment cycles. The first parameter relates to the remaining functionality of the affected joint affects and how this functionality impacts the quality of life of the patient and activities of daily living. In order to do this assessment, validated outcome score systems for at least the major joints such as hip, knee, shoulder, elbow and wrist are available in tandem with quality-of-life scoring. Assessment of joint function should be continued for at least 2 years (1,2). As a consequence of significant functional deterioration, the diseased joint and adjacent soft tissue and bone structures in large joints might require adequate surgical site removal with a treatment strategy of a 2-stage joint replacement within this two-year period. After this period, a relatively successful outcome by a single stage joint replacement could be achieved subject to absence of persistent native joint infection as demonstrated by a stable clinical status and favorable serology and synovial fluid analysis.

The second key element when evaluating the treatment outcome of native joints after septic arthritis refers to the need for additional surgical remedial action to deal with the sequelae, i.e. corrective osteotomies after partial growth plate arrest in skeletally immature patients, open total synovectomies, capsular and joint contracture releases, resection arthroplasties, arthrodesis and joint replacements in adults.
The third key element appears to be increased mortality. Predicting factors for this devastating outcome in the papers studied were hip infection, shoulder infection, multiple-site infection, being male, age ≥65 years old and comorbidities (diabetes mellitus and chronic renal failure) (3,4). A diagnosis of a secondary septic arthritis versus a primary one was also associated with increased mortality (5).

References:

1.- Bauer T, Lacoste S, Lhotellier L, Mamoudy P, Lortat-Jacob A, Hardy P. Arthroplasty following a septic arthritis history: a 53 cases series. Orthop Traumatol Surg Res. 2010;96:840–843.
2.- Sultan AA, Mahmood B, Samuel LT, George J, Faour M, Pelt CE, Anderson MB, Klika AK, Higuera CA. Patients with a History of Treated Septic Arthritis are at High Risk of Periprosthetic Joint Infection after Total Joint Arthroplasty. Clin Orthop Relat Res. 2019;477(7):1605-1612.
3.- Maneiro JR, Souto A, Cervantes EC, Mera A, Carmona L, Gomez-Reino JJ. Predictors of treatment failure and mortality in native septic arthritis. Clin Rheumatol. 2015;34(11):1961-7.
4.- Huang YC, Ho CH, Lin YJ, Chen HJ, Liu SY, Wang CL, Lin CH, Wang JJ, Chien CC. Site-specific mortality in native joint septic arthritis: a national population study. Rheumatology (Oxford). 2020 Dec 1;59(12):3826-3833.
5.- Abram SGF, Alvand A, Judge A, Beard DJ, Price AJ. Mortality and adverse joint outcomes following septic arthritis of the native knee: a longitudinal cohort study of patients receiving arthroscopic washout. Lancet Infect Dis. 2020;20(3):341-349.

GRADE:

STUDY	***YEAR***	***TYPE OF STUDY***	**EVIDENCE QUALITY**
Bauer T (1)	*2010*	*Retrospective cohort series/observational*	⊕ ⊖ ⊖ ⊖ *Very Low* *Very small sample size*
Sultan (2)	*2019*	*Retrospective cohort series/observational*	⊕ ⊖ ⊖ ⊖ *Very Low* *Very small sample size*
Maneiro(3)	*2015*	*Retrospective cohort series/observational*	⊕ ⊖ ⊖ ⊖ *Low* *Small sample size*
Huang YC(4)	*2020*	*Retrospective cohort series/observational*	⊕ ⊕ ⊕ ⊖ *Moderate* *Large sample size*
Abram SGF (5)	*2020*	*Retrospective cohort series/observational*	⊕ ⊕ ⊕ ⊖ *Moderate* *Large sample size*

Study	*year*	*Type of study*	*Evidence quality*
Peres LR	*2016*	*RCT*	*⊕⊕⊕⊖*<br>*Moderate*<br>*No blinding, small size*
Wirtz DC	*2001*	*Case-control*	*⊕⊕⊖⊖*<br>*Low*
Böhler C	*2016*	*Case-control*	*⊕⊕⊖⊖*<br>*Low*
Johns BP	*2017*	*Cohort*	*⊕⊕⊖⊖*<br>*Low*<br>*They use OR in a cohort study, RR would be much more appropriate*
Faour M	*2019*	*Case- Control*	*⊕⊕⊕⊖*<br>*Moderate*<br>*Though is an observational study sample size being large and the effect size is important.*
Stutz G	*2000*	*Case series*	*⊕⊖⊖⊖*<br>*Very low*
Balabaud L	*2007*	*Consecutive series. Case-control*	*⊕⊕⊖⊖*<br>*Low*
Aim F	*2015*	*Observational*	*⊕⊖⊖⊖*<br>*Very low*
Felck E	*2011*	*Case series*	*⊕⊖⊖⊖*<br>*Very low*
Shaikh AA	*2014*	*Case series*	*⊕⊖⊖⊖*<br>*Very low*
Hochreiter B	*2016*	*Case series*	*⊕⊖⊖⊖*<br>*Very low*
Kodumuri P	*2012*	*Retrospective Cohort*	*⊕⊕⊖⊖*<br>*Low*
Lauper N	*2018*	*Retrospective Cohort*	*⊕⊕⊕⊖*<br>*Moderate*<br>*Though is a retrospective study sample size being large and the effect size is important.*
Dave OH	*2016*	*Retrospective cohort*	*⊕⊕⊖⊖*<br>*Low*

In line with other nonspecific patient related outcome scoring systems such as the Musculo-Skeletal Tumor Rating Scale (MSTS-87) or the Toronto Extremity Salvage Score (TESS) and joint specific outcome scoring systems such as e.g. the Knee Society Score, the Harris Hip Score, the AO Foot Ankle Score, the disabilities of the arm, shoulder and hand (DASH) questionnaire, the Constant-Murley Shoulder Score, the development of integrated and validated outcome scoring instruments for septic arthritis of the native joint would be beneficial to compare treatment modalities and to determine failure versus success.

Inclusion of quality of life measurement scales such as the ASK (Activity Scale for Kids), the SRS (Session Rating Scale for Kids), WorldHealthQuality of Life Toolkits, the 36 or 12 Short Form Health Survey ,… should be evident.

Further research with at least well-designed cohort or case-control studies is required to provide more robust quality of evidence.
