# Supplementary material for: Guideline for management of septic arthritis in native joints (SANJO)
Source: J Bone Jt Infect. 2023 Jan 12;8(1):29–37. doi: 10.5194/jbji-8-29-2023 (PMC9901514; doi:10.5194/jbji-8-29-2023)
Supplement: The supplement related to this article is available online at: https://doi.org/10.5194/jbji-8-29-2023-supplement. [file jbji-8-29-supplement.zip › Workgroup report 4, empirical antibiotic treatment of SANJO.pdf]

EBJIS guideline Workgroup 4: Empirical antimicrobial treatment

Authors:

Efthymia Giannitsioti (Dept. of Infectious Diseases, Attikon University General Hospital, Athens, Greece)

Svetlana Bohkova (Dept. of Orthopaedic Surgery and Traumatology, University Hospital of St. Petersburg, Russia)

Miguel Araújo Abreu (Dept. of Infectious Diseases, Centro Hospitalar do Porto, Portugal)

Is empirical treatment as effective as culture-guided treatment in SA?

Evidence regarding the correct moment to start, the choice and the duration of antibiotic therapy in septic arthritis (SA) is limited because large randomized controlled trials have not been performed in adults and relevant data is scarce. [1].

The isolation of the microorganism in patients with SA may be hampered if antibiotics are administered before obtaining samples for microbiological evaluation. These patients were significantly less likely to demonstrate a microorganism on microscopy and on culture, leading to a 50% drop in the sensitivity of synovial fluid cultures [2]. Although some data showed that patients treated only upon a clinical evaluation of SA had a comparable morbidity and mortality than those who were treated upon microbiological proof of the infection [4], whenever possible, it is advisable to obtain a synovial fluid sample before starting antibiotics. If clinical suspicion of SA is high (patients with a short history of a warm, swollen and tender joint (or joints) with restriction of movement with or without fever) and sample collection is not possible, then it is correct to start antibiotic treatment even before obtaining samples for culture [3].

The initial choice of antibiotics is based upon synovial fluid Gram stain, prevalence of drug resistance of causative microorganisms in a particular geographic area, the presence of risk factors for specific pathogens and the severity of the infection [1,4]. Methicillin-susceptible *S. aureus* (MSSA) is the predominant pathogen, and it has been identified in septic arthritis of native joints in approximately 45-65% of cases followed by *Streptococcus* spp. (15%) [5–9]. The incidence of *Neisseria gonorrhoeae* is low, but it should be suspected in young and sexually active people. Rates of MRSA are reported up to 5% [5–7] although in some regions like the US is the most common pathogen. Gram-negative bacteria (GNB) are less frequently involved in SA (15-17%) being *P. aeruginosa* and *E. coli* the most frequent isolates [5,6,10]. Elderly and immunecompromised patients have a higher risk for GNB. The incidence of MRSA and *P. aeruginosa* are higher in patients with intravenous drug users [9,11]. According to the complexity of the etiology, changing epidemiology and increasing rates of resistant pathogens, a culture-driven antibiotic administration is preferable to an empirical one.

Timing of initiation of empirical antimicrobial treatment: immediate after synovial fluid /tissue sampling or afterwards?

Although the timing of initiation of empirical antimicrobial treatment is of paramount importance, no randomized trials or observational comparative studies with satisfactory design were published. A common recommendation suggests that empirical administration of antibiotics in case of suspicion for septic arthritis should be deferred until the synovial fluid is drawn for cultures[12]. Patients with septic arthritis of the knee receiving antibiotics before laboratory tests were less likely to demonstrate a microorganism on microscopy or culture of the synovial aspirate [2]. Therefore, empirical antibiotic therapy should be commenced after arthrocentesis and further adjusted following the results of microbiological cultures. In septic patients, the treatment should be started within the first hour of admission [13], therefore, synovial fluid and tissue sampling should never delay the prompt start of antibiotic therapy [strong evidence, case control studies]. In non-septic patients even though the lack of solid evidence, it appears that the delay of initiation of antibiotic therapy is related to treatment failure[14].

Recommendation 1:

Considering that diagnostic yield is reduced by empirical antibiotics' administration and that antimicrobial resistance emerge worldwide, we should consider that all attempts for a prompt microbiological documentation of SA should be exhausted before starting treatment (weak evidence, expert opinion). Antimicrobial therapy should be started without delay once specimens of joint fluid for culture are obtained [weak evidence, expert opinion].

2. Empirical treatment: broad or narrow antibiotic spectrum?

Regarding antimicrobial treatment of native septic arthritis, few studies exist often with poor methodological quality. Meta-analysis on antibiotic therapy in bone and joint infections have a high heterogeneity often including both native and prosthetic joint infections and osteomyelitis [15]. Furthermore, data on the use of narrow or broad-spectrum antibiotics for empirical treatment of patients with septic arthritis in native joints are very limited. Data search revealed only small, retrospective case studies, often with a mixed population of native and prosthetic joint infections [1,9,15]. Epidemiological trends of resistance in the community and in health-care settings are important as they conduct the need of a narrow or broad antimicrobial treatment. In settings where no third-generation cephalosporin resistant GNB are detected and rates of MRSA do not increase within years, narrow spectrum antibiotics are appropriate empirical treatment in SA [16]. However, emerging antimicrobial resistance mainly by extended-spectrum β-lactamases (ESBL) can affect even up to 20% of all isolates. In SA caused by GNB in Taiwan, a 25% of Enterobacterales and 20% of non-fermenting GNB were resistant to ceftriaxone and ceftazidime, respectively [10]. In the US, MRSA was the most common pathogen in community-acquired SA in adults [17]. Therefore, new concerns arise for the narrow spectrum antibiotics -including cephalosporins- as empirical initial treatment and highlights the need for a continuous epidemiological surveillance of antimicrobial resistance worldwide [15,18].

The importance of starting active empirical antibiotic has been recurrently documented in patients with sepsis. The experience in SA is scarce but a short

case series caused by *S. aureus* (15 MRSA and 43 MSSA) suggested a benefit of adequate empirical treatment. The majority of MRSA cases received inappropriate empirical therapy (93%) while no one of the MSSA cases. The sepsis related mortality was higher for MRSA (13% versus 5%), although the difference was not statistically significant [19].

Empirical treatment should be selected according to: 1) the result of Gram staining (gram positive or gram negative) and morphology (cocci forming grapes or chains, bacilli), 2) the presence of risk factors for MRSA, or resistant gram negatives including *P. aeruginosa* and cephalosporin-resistant Enterobacterales [4], and 3) the severity of the infection. The selection of empirical antibiotic treatment according to the Gram staining, morphology and the presence of risk factors is depicted in table 1. A matter of debate is from which percentage of risk we must start empirical antibiotic treatment to cover a broader spectrum of microorganisms [1,20]. There is no established breakpoint but a prevalence ≥10% would be reasonable and ≥5% in patients with severe infection (septic shock). These are the breakpoints included in table 1. Unfortunately, on many occasions the Gram staining is negative, and the empirical treatment should be selected based on epidemiology and the presence of risk factors (table 1).

Table 1. Antibiotic options for empirical treatment in patients with native septic arthritis according to the Gram staining result.

**Gram staining**	**Antibiotic**
Grampositive cocci in grapes:	
- No risk for MRSA (≤10% or ≤5% in patients with septic shock)	Cefazolin 2g TID iv<br>Flu-cloxacillin 2g QID iv
- Risk of MRSA[1] or penicillin allergy	Vancomycin[2] 15-20mg/Kg BID iv<br>Teicoplanin[3] 10mg/kg QD iv, im, sc

	Linezolid[4] 600mg BID iv, po<br>Daptomycin[5] 8-10mg/kg QD iv<br>Ceftaroline[6] 600mg BID or TID iv<br>Ceftobiprole[6] 500mg TID iv<br>Dalbavancin[7] (see reference 30)<br>Telavancin[8] 10mg/kg QD iv<br>Fosfomycin[9] 8g TID iv
Gram positive cocci in chains	Ceftriaxone 1g BID iv or 2g QD<br>Cefotaxime 2g TID iv
Gram negative cocci[10]	Ceftriaxone 1g QD iv or 2g QD<br>Cefotaxime 1g TID iv
Gram negative bacilli<br>- Without risk factors<br>- With risk factors for *P. aeruginosa* or resistance to 3rd generation cephalosporins[11]	 Ceftriaxone 1g BID iv or 2g QD<br>Cefotaxime 2g TID iv<br>Meropenem[12] 2g TID iv ± amikacin[13] 15-20 mg/kg QD iv
Negative Gram staining<br>- No risk factors<br>- With risk factors	 Cefazolin 2g TID iv or<br>Flu-cloxacillin 2g QID + ceftriaxone 1g BID<br>Vancomycin 15-20mg/kg BID (or other anti-MRSA) + Meropenem 2g TID iv

MRSA, methicillin-resistant *S. aureus*. QD, once daily. BID, twice daily. TID, three times daily. QID, four times daily. Iv, intravenous route. Im, intramuscular. Sc subcutaneous route. Po, oral route.

[1] Risk factors for MRSA include: a) prior colonization or infection by MRSA, b) prevalence of MRSA ≥10% in the population, c) intravenous drug user, and d) the presence of ≥2 of the following: 1) coming from a long-term care facility or prior admission in an acute care center in the past 3 months, 2) chronic renal failure or 3) the patient has received a fluoroquinolone in the past 3 months.

[2] In case of one of the following situations select for other alternative: 1) patients with renal failure, and 2) the prevalence of MIC for vacomycin ≥1 mg/L (automated systems) or ≥1.5 mg/L (E-test) is ≥10%.

[3] Teicoplanin was proven effective in the treatment of osteomyelitis and SA [21]. Subcutaneous or intravenous route of administration have shown similar plasma concentrations [22].

[4] Linezolid has 100% oral bioavailability being a good option for oral therapy. It has demonstrated good results in bone and joint infections [23].

[5] Daptomycin has demonstrated effectiveness in bone and joint infections [1,24].

[6] New fifth-generation cephalosporins with activity against MRSA and good results as salvage treatment in complex bone and joint infections [25].

[7] Lipoglycopeptide with a long half-life that can be administered once a week or every 2 weeks. For the dosing, to cover 30 to 60 days, we recommend reading the reference 31 [26]. It has proven efficacy in patients with bone and joint infections[27].

[8] Lipoglycopeptide with shorter half-life than dalbavancin with proven efficacy in 40 patients with SA. It can be a second line choice in countries where the drug is available [28].

[9] Fosfomycin is very active against MRSA and synergistic with other antibiotics (beta-lactams, daptomycin, linezolid). It is recommended to use in combination [29].

[10] In cases with *N. gonorrhoeae* it is advisable to empirically cover *Chalmydia trachomatis* giving a single dose of 2 g of azithromycin.

[11] Risk factors include: 1) severe immunosuppression, 2) intravenous drug users, 3) prior antibiotic therapy in the past 3 months or 4) prior infection or colonization.

[12] In regions or patients with low prevalence of resistance to third generation cepphalosporins, potential alternatives for meropenem are piperacillin/ tazobactam 4g QID iv or ceftazidime 2g TID iv or cefepime 2g TID iv [30].

[13] For patients at risk of multidrug or extensively drug resistant gram negative bacteria (immunocompromised host, healthcare or nursing homes, pre-colonized patients) including carbapenemase-producing GNB. Novel b-lactams (ceftolozane/tazobactam, ceftazidime /avibactam) could be considered in the context of Infectious diseases consultation [31].

Recommendation 2:

Empirical treatment should be selected according to: 1) the result of Gram staining (grampositive or gramnegative) and morphology (cocci forming grapes or chains, bacilli), 2) the presence of risk factors for MRSA, or resistant gramnegatives including *P. aeruginosa* and cephalosporin-resistant Enterobacterales [4], and 3) the severity of the infection [weak evidence, expert opinion].

3. Guided antimicrobial treatment

Empirical treatment should be tailored once the microbiological data from blood/synovial/tissue cultures is available. De-escalation in susceptible pathogens is mandatory. It is recommended to maintain intravenous administration for 1-2 weeks followed by 2-4 weeks of oral antimicrobial agents. In total, a 4–6-week treatment is suggested although no robust data exist. Up to 4-6 weeks treatment is preferable for large native joint SA but shorter duration (<4 weeks) could be enough for small native joint SA [32]. For *Neisseria gonorrhoeae* SA, a 7-day regimen is effective.

The selection of oral antibiotic should be based on the in vitro activity, oral bioavailability, and the diffusion to the synovial fluid [33]. Oral options are summarized in table 2.

Table 2. Oral antibiotic treatment for patients with septic arthritis

**Microorganism**	**Oral antibiotic**
S. aureus[1]	Amoxicillin/clavulanate 875/125mg TID<br>Levofloxacin[2] 500-750mg QD<br>Moxifloxacin[2] 400mg QD<br>Linezolid[3] 600mg BID<br>Tedizolid[3] 200mg QD<br>Clindamycin 600mg TID<br>Cotrimoxazole[4] 160/800mg BID<br>Minocycline 100mg BID<br>Fusidic acid Na 500mg TID
Streptococcus spp	Amoxicillin 1g TID<br>Cefalexin 1g TID<br>Clindamycin 600mg TID
Gramnegatives	Ciprofloxacin 500-750mg BID<br>Amoxicillin/clavulanate 875/125mg TID<br>Cefixime 400mg QD or BID

[1] The addition of rifampicin (600mg QD or 450mg BID) is proven to improve outcome in prosthetic joint infections [34]. Although relevant data on native joint infection is lacking, it is reasonable to recommend the combination with fluoroquinolones.

[2] Oral fluoroquinolones were proven to be non-inferior to standard beta-lactam treatment in patients with chronic osteomyelitis [35,36]. Moreover, the combination of rifampicin with either ciprofloxacin [34] or levofloxacin [37] were effective in patients with prosthetic joint infection, but relevant data in SA is not available.

[3] For >2 weeks of treatment with linezolid, close monitor of adverse events is necessary. The most important are gastrointestinal events, fatigue, anemia and thrombocytopenia. Tedizolid has been associated with less frequency of adverse events.

[4] Cotrimoxazole has been used in combination with other agents as fluoroquinolones, rifampin and beta-lactams [38].

Unfortunately, according to a recent retrospective analysis including 543 native joint SA only 40% of SA had a positive synovial fluid [32], therefore, treatment should be based on the risk factors for resistant pathogens. In these patients, it is advisable to obtain a nasal swab to determine whether the patient is a carrier of *S. aureus* and a rectal swab to discard the presence of multi-drug resistant GNB. Although the information in patients with SA is lacking, the negative predictive value of these samples is high in other infections.

Recommendation 3:

De-escalation according to the susceptibility pattern is mandatory. It is recommended to maintain intravenous administration for 1-2 weeks followed by 2-4 weeks of oral antimicrobial agents when possible [weak evidence, expert opinion].

References

1. Mathews CJ, Weston VC, Jones A, Field M, Coakley G. Bacterial septic arthritis in adults. Lancet **2010**; 375:846–855.

2. Hindle P, Davidson E, Biant L. Septic arthritis of the knee: the use and effect of antibiotics prior to diagnostic aspiration. Ann Roy Coll Surg **2012**; 94:351–355.

3. Gupta MN, Sturrock RD, Field M. Prospective comparative study of patients with culture proven and high suspicion of adult onset septic arthritis. Annals of the rheumatic diseases **2003**; 62:327–331.

4. Coakley G, Mathews C, Field M, et al. BSR & BHPR, BOA, RCGP and BSAC guidelines for management of the hot swollen joint in adults. Rheumatology **2006**; 45:1039–1041.

5. Clerc O, Prod'hom G, Greub G, Zanetti G, Senn L. Adult native septic arthritis: a review of 10 years of experience and lessons for empirical antibiotic therapy. J Antimicrob Chemoth **2011**; 66:1168–1173.

6. Maneiro JR, Souto A, Cervantes EC, Mera A, Carmona L, Gomez-Reino JJ. Predictors of treatment failure and mortality in native septic arthritis. Clin Rheumatol **2014**; 34:1961–1967.

7. Muñoz-Egea M-C, Blanco A, Fernández-Roblas R, et al. Clinical and microbiological characteristics of patients with septic arthritis: A hospital-based study. J Orthop **2014**; 11:87–90.

8. Kennedy N, Chambers ST, Nolan I, et al. Native Joint Septic Arthritis: Epidemiology, Clinical Features, and Microbiological Causes in a New Zealand Population. J Rheumatology **2015**; 42:2392–2397.

9. Ross JJ. Septic Arthritis of Native Joints. Infect Dis Clin N Am **2017**; 31:203–218.

10. Lin W-T, Tang H-J, Lai C-C, Chao C-M. Clinical manifestations and bacteriological features of culture-proven Gram-negative bacterial arthritis. J Microbiol Immunol Infect **2017**; 50:527–531.

11. Allison DC, Holtom PD, Patzakis MJ, Zalavras CG. Microbiology of Bone and Joint Infections in Injecting Drug Abusers. Clin Orthop Relat Res **2010**; 468:2107–2112.

12. Amanatullah D, Dennis D, Oltra EG, et al. Hip and Knee Section, Diagnosis, Definitions: Proceedings of International Consensus on Orthopedic Infections. J Arthroplast **2019**; 34:S329–S337.

13. Sherwin R, Vilke GM, Wardi G. DOES EARLY AND APPROPRIATE ANTIBIOTIC ADMINISTRATION IMPROVE MORTALITY IN EMERGENCY DEPARTMENT PATIENTS WITH SEVERE SEPSIS OR SEPTIC SHOCK? The Journal of Emergency Medicine **2017**;

14. Valour F, Bouaziz A, Karsenty J, et al. Determinants of methicillin-susceptible Staphylococcus aureusnative bone and joint infection treatment failure: a retrospective cohort study. Bmc Infect Dis **2014**; 14:443.

15. Stengel D, Bauwens K, Sehouli J, Ekkernkamp A, Porzsolt F. Systematic review and meta-analysis of antibiotic therapy for bone and joint infections. Lancet Infect Dis **2001**; 1:175–188.

16. Ben-Chetrit E, Zamir A, Natsheh A, Nesher G, Wiener-Well Y, Breuer GS. Trends in antimicrobial resistance among bacteria causing septic arthritis in adults in a single center: A 15-years retrospective analysis. Intern Emerg Med **2020**; 15:655–661.

17. Frazee BW, Fee C, Lambert L. How Common is MRSA in Adult Septic Arthritis? Ann Emerg Med **2009**; 54:695–700.

18. George J, Chandy VJ, Premnath J, et al. Microbiological Profile of Septic Arthritis in Adults: Lessons Learnt and Treatment Strategies. Indian J Med Microbi **2019**; 37:29–33.

19. Al-Nammari SS, Bobak P, Venkatesh R. Methicillin resistant Staphylococcus aureus versus methicillin sensitive Staphylococcus aureus adult haematogenous septic arthritis. Arch Orthop Traum Su **2007**; 127:537–542.

20. Berendt T, Byren I. Bone and joint infection. Clin Med **2004**; 4:510–518.

21. LeFrock J, Ristuccia A. Teicoplanin in the treatment of bone and joint infections: An open study. Journal of Infection and Chemotherapy **1999**; 5:32–39.

22. Peeters O, Ferry T, Ader F, et al. Teicoplanin-based antimicrobial therapy in Staphylococcus aureus bone and joint infection: tolerance, efficacy and experience with subcutaneous administration. Bmc Infect Dis **2016**; 16:622.

23. Falagas ME, Siempos II, Papagelopoulos PJ, Vardakas KZ. Linezolid for the treatment of adults with bone and joint infections. International Journal of Antimicrobial Agents **2007**; 29:233–239.

24. Lalani T, Boucher HW, Cosgrove SE, et al. Outcomes with daptomycin versus standard therapy for osteoarticular infections associated with Staphylococcus aureus bacteraemia. J Antimicrob Chemoth **2008**; 61:177–182.

25. Malandain D, Dinh A, Ferry T, et al. Salvage therapy for complex bone and joint infections with ceftaroline: a multicentre, observational study. Int J Antimicrob Ag **2017**; 50:277–280.

26. Cojutti PG, Rinaldi M, Zamparini E, et al. Population pharmacokinetics of dalbavancin and dosing consideration for optimal treatment of adult patients with staphylococcal osteoarticular infections. Antimicrob Agents Ch **2021**;

27. Morata L, Cobo J, Fernández-Sampedro M, et al. Safety and Efficacy of Prolonged Use of Dalbavancin in Bone and Joint Infections. Antimicrobial Agents and Chemotherapy **2019**; 63:1405.

28. Sims CR, Bressler AM, Graham DR, Lacy MK, Lombardi DA, Castaneda-Ruiz B. Real-World Clinical Use and Outcomes of Telavancin for the Treatment of Bone and Joint Infections: Results from the Telavancin Observational Use Registry (TOUR™). Drugs - Real World Outcomes **2021**; :1–10.

29. Morata L, Soriano A. The role of fosfomycin in osteoarticular infection. Revista española de quimioterapia : publicación oficial de la Sociedad Española de Quimioterapia **2019**; 32 Suppl 1:30–36.

30. Shirtliff ME, Mader JT. Acute Septic Arthritis. Clin Microbiol Rev **2002**; 15:527–544.

31. Vena A, Castaldo N, Bassetti M. The role of new β-lactamase inhibitors in gram-negative infections. Curr Opin Infect Dis **2019**; 32:638–646.

32. McBride S, Mowbray J, Caughey W, et al. Epidemiology, management and outcomes of large and small native joint septic arthritis in adults. Clinical infectious diseases : an official publication of the Infectious Diseases Society of America **2019**;

33. Thabit AK, Fatani DF, Bamakhrama MS, Barnawi OA, Basudan LO, Alhejaili SF. Antibiotic penetration into bone and joints: An updated review. Int J Infect Dis **2019**; 81:128–136.

34. Zimmerli W, Widmer AF, Blatter M, Frei R, Ochsner PE. Role of rifampin for treatment of orthopedic implant-related staphylococcal infections: a randomized controlled trial. Foreign-Body Infection (FBI) Study Group. JAMA: The Journal of the American Medical Association **1998**; 279:1537–1541.

35. Mader JT, Cantrell JS, Calhoun J. Oral ciprofloxacin compared with standard parenteral antibiotic therapy for chronic osteomyelitis in adults. J Bone Jt Surg Am Volume **1990**; 72:104–10.

36. Greenberg RN, Tice AD, Marsh PK, et al. Randomized trial of ciprofloxacin compared with other antimicrobial therapy in the treatment of osteomyelitis. Am J Medicine **1987**; 82:266–9.

37. Nguyen S, Robineau O, Titecat M, et al. Influence of daily dosage and frequency of administration of rifampicin–levofloxacin therapy on tolerance and effectiveness in 154 patients treated for prosthetic joint infections. Eur J Clin Microbiol **2015**; 34:1675–1682.

38. Deconinck L, Dinh A, Nich C, et al. Efficacy of cotrimoxazole (Sulfamethoxazole-Trimethoprim) as a salvage therapy for the treatment of bone and joint infections (BJIs). Plos One **2019**; 14:e0224106.
