# Supplementary material for: Guideline for management of septic arthritis in native joints (SANJO)
Source: J Bone Jt Infect. 2023 Jan 12;8(1):29–37. doi: 10.5194/jbji-8-29-2023 (PMC9901514; doi:10.5194/jbji-8-29-2023)
Supplement: The supplement related to this article is available online at: https://doi.org/10.5194/jbji-8-29-2023-supplement. [file jbji-8-29-supplement.zip › Workgroup report 9, Pediatric SANJO.pdf]

EBJIS guideline Workgroup 9: Pediatric septic arthritis

AUTHORS AND AFFILIATIONS

Dr. Markus Pääkkönen (Turku University Hospital, Turku, Finland)

Dr. Marko Pokorn (Ljubljana University Medical Center, Ljubjana, Slovenia)

Dr. Jesus Saavedra (Hospital Universitario Gregorio Marañon, Madrid, Spain)

Dr. Martin Gottliebsen (Aarhus University Hospital, Aarhus, Denmark)

RELATED DISCLOSURES OF AUTHORS

Dr. Markus Pääkkönen:	Nothing to disclose
Dr. Marko Pokorn	Received research grant from Pfizer.
Dr. Jesus Saavedra	Nothing to disclose
Dr. Martin Gottliebsen:	Nothing to disclose

Background and clinical application

Introduction

The traditional treatment for paediatric septic arthritis (SA) has consisted of long courses of antibiotics started intravenously, and aggressive surgery. During the recent decade, shorter duration of antibiotic treatment and a more conservative approach to surgery besides diagnostic arthrocentesis has been recommended [1-4].

Antibiotic therapy

The initial empiric antibiotic therapy should cover *S. aureus* in all age groups. The epidemiological characteristics suggest that first generation cephalosporins, anti-staphylococcal penicillins or clindamycin should be used in the initial antibiotic therapy of SA in children with methicillin-sensitive S. aureus (MSSA). There is a paucity of clinical trials comparing the efficacy of different antibiotics in SA with only one prospective,

randomized study comparing clindamycin vs 1st generation cephalosporins [4]. This study did not find differences between the two antibiotics and found short intravenous therapy followed by oral therapy to be safe and efficacious. It should be noted that the study did not include patients with community acquired methicillin resistant S.aureus (ca-MRSA). In areas where MRSA has a high incidence (10-15% of *S. aureus* infections) an antibiotic with good activity against this agent as well as against MSSA should be used. Clindamycin is a good option if the rate of clindamycin-resistant ca-MRSA is low, and always keeping in mind that a small percentage of MSSA may be resistant to clindamycin [2-9]. Vancomycin, linezolid and trimethoprim – sulfamethoxazole (TMP-SMX) are used for ca-MRSA [10,11].

In children, the rate of penicillin or cefotaxime-resistant *S. pneumoniae* outside the central nervous system is very low in our setting, being around 3% for invasive pneumococal infection. In the United States the rates of resistance to B-lactams are also very low ranging 3-6% of pneumococcal SA. Therefore, B-lactams (especially penicillin or ampicillin/amoxicillin) are a suitable first line therapy for pneumococcal SA in children. For group A streptococcus (GAS) and group B streptococcus (GBS), penicillin is still first line therapy recommended by most of experts. For children allergic to penicillin, clindamycin may be a suitable alternative for *Streptococci* SA, although a variable rate of these bacteria may be resistant [4].

Randomized controlled trials have shown two to five days of intravenous therapy followed by oral therapy for a total of 2-3 weeks (minimum 10 days) to be appropriate [4,7,8]. SA caused by MRSA, *Salmonella* spp. or Panton-Valentine leukocidin (PVL)-positive strains may need longer duration of both intravenous and oral therapy based on the severity of osteoarticular infections caused by these microorganisms with an increased rate of complications.

Adjuvant therapy

Several studies have evaluated the effect of corticosteroids in the outcome of children with SA. A randomized, placebo-controlled study done in Costa Rica evaluated the impact of treatment with antibiotics associated to dexamethasone (DXM) or placebo for 4 days on the outcome of 100 children with bacteriologically confirmed SA, predominantly caused by *S. aureus*. Treatment with DXM shortened the duration of symptoms, and after 1 year of follow-up sequelae were found in in a significantly lower rate in the DXM group (26 vs. 2%) [12].

Surgery

Significant controversy remains regarding operative treatment besides diagnostic arthrocentesis. One prospective, and several retrospective studies have shown arthrocentesis to be appropriate approach for SA therapy in children also in hip and shoulder arthritis. It has been suggested that arthrotomy may be reserved for cases with delayed presentation or that are resistant to therapy [13,14].

Prognosis and follow-up

The aim of the short-term follow-up of children with SA is to confirm that the infection has been eradicated. Prospective studies in children with SA demonstrated that antibiotics could be safely stopped once the patient is asymptomatic or has minor symptoms, CRP is normal (e.g. < 2 mg/dL), after a minimum length of treatment (10-14 days for non-complicated SA) [4]. The precise rate of intra-articular epiphyseal injuries or growth disorders is not known. Risk may vary according to the duration of symptoms before the treatment is initiated, age of the patient, localization of septic arthritis and the causative agent.

The followup should include a thorough clinical examination and C-reactive protein measurements. Radiographs or MRI may be done on demand. Salmonella and MRSA are related to higher rate of complications and may require a more frequent follow-up.

In a prospective treatment trial on 130 children with culture-positive septic arthritis no patient experienced residual dysfunction. Most data regarding septic arhtritis is from retrospective cohort studies. In a large epidemiology study from France on 1359 patients with septic arthritis, only 2 deaths occurred, of which one patient was undergoing treatment for leukemia [15]. There are only few reports from low-resource countries, where the disease burden is the highest. The quality of the studies varies making comparison between different regions difficult.

Summary of recommendations

1. When and how to do diagnostic sampling

Recommendations

1. When to do diagnostic sampling

Joint drainage by aspiration is recommended after the diagnosis of SA is suspected. A diagnostic sample for joint culture should always be obtained. Blood cultures should always be obtained. [IIB]

2. How to do diagnostic sampling

The sample for bacteriology may be be taken by needle aspiration of the joint, (arthroscopy or arthrotomy). Ultrasound or fluoroscopy may be used to assist in the procedure. Diagnostic yield can be increased by inoculating joint fluid into a blood culture vial or using molecular methods for pathogen detection. [IIB]

2. Empiric antibiotic therapy

Recommendations

1. When to start empiric antibiotic therapy

Empirical therapy should be initiated immediately once appropriate cultures have been obtained. [IIA]

2. Primary antibiotics

S. aureus should be targeted in all age groups. The epidemiological characteristics suggest that first generation cephalosporins, anti-staphylococcal penicillins or clindamycin should be used in the initial antibiotic therapy of SA in children. [IIA]

3. Special considerations for antimicrobial treatment in neonates

Antibiotic treatment should also cover enterobacteria. [IIA]

4. Special considerations for antimicrobial treatment in children < 5 years

Kingella kingae should also be covered. *Haemophilus influenzae* type B and *Streptococcus pneumoniae* should be considered in unvaccinated children and in cefuroxime may be considered instead of 1st generation cephalosporins. [IIIB]

5. Special considerations regarding community acquired methicillin-resistant *S. aureus* (ca-MRSA)

Empirical therapy should include an appropriate coverage against methicillin resistant *S. aureus* (ca-MRSA) in areas with more than 10-15% prevalence of this bacterium. [IIA]

6. Empiric antibiotic therapy for ca-MRSA

Clindamycin in monotherapy may be appropriate in most of the cases unless the infection is very severe or there is a high rate of clindamycin-resistant MRSA, where other antibiotics such as glycopeptides, daptomycin or linezolid should be used. [IIIB]

3. Targeted antibiotic therapy

1. Targeted antibiotic therapy for methicillin-sensitive *S. aureus* (MSSA)

For MSSA, an anti-staphylococcal β-lactam such as cefazolin or anti-staphylococcal penicillin may be appropriate. Clindamycin may be used, but some strains can be resistant to this antibiotic. [IIA]

2. Targeted antibiotic therapy for ca-MRSA

Clindamycin is suitable for susceptible strains. For clindamycin-resistant strains, a glycopeptide or other appropriate antibiotic for MRSA, such as linezolid or daptomycin should be chosen. [IIIB]

3. Targeted antibiotic therapy for *K. kingae*

For the treatment of *K. kingae* SA, penicillin may be a suitable antibiotic following the general recommendations. [IIB]

4. Targeted antibiotic therapy for group A and group B streptococcus (GAS, GBS) and *Streptococcus pneumoniae*

In children, penicillin is chosen for GAS and GBS, and for penicillin-susceptible *S. pneumoniae*. If *S. pneumoniae* is detected by molecular techniques and no antibiogram is available, penicillin may still be a suitable antibiotic if the rate of penicillin resistance for this bacterium in the community is low. [IIIB]

5. Atypical pathogens

SA caused by *Salmonella* spp, *Pseudomonas* spp or enterobacteriaceae may need to adjust antibiotics according to susceptibilities and may need prolonged IV/PO therapy (e.g. total of 4-6 weeks), and a more aggressive approach, especially in young infants or in children with underlying diseases. [IIIB]

6. Oral treatment

Sequential oral treatment with first generation cephalosporins or clindamycin are good options when guided by the antibiotic susceptibilities of the bacteria. Same doses are used for intravenous and oral treatment. [IIA] In the case of MRSA, TMP-SMX may be an option and can be combined with rifampin. [IIIB] Fluoroquinolones or linezolid are other options for oral treatment. [IIIB]

4. Duration of antibiotic therapy

1. Duration of intravenous antibiotic therapy

The minimum duration of intravenous antibiotic therapy for uncomplicated SA is 2-4 days. [IA]

2. Duration of oral antibiotic therapy

The duration of therapy for non-complicated SA is 2-3 weeks. [IA]

3. Special cases

Complicated or high-risk cases such as those produced by *Salmonella*, MRSA or Panton-Valentine leukocidin (PVL)-positive strains, infections developing in young infants, or with slow clinical improvement, may need to receive longer duration (total of 4-6 weeks) of both intravenous (IV) and oral therapy. The duration of therapy for infants with SA, including those under 4 months of age, may be 4-6 weeks, with a minimum of 7-14 days of IV therapy. [IIB]

5. Adjuvant therapy

1. Non-steroidal anti-inflammatory drugs (NSAID)

Anti-inflammatory therapy may be of benefit in patients with SA and NSAIDs should be appropriately provided as needed according to pain and inflammation. [IIIA]

2. Corticosteroids

IV corticosteroids as adjuvant therapy to antibiotics may hasten clinical recovery and decreased hospital stay in children with SA. The long-term effect is not known. [IB]

3. Physical therapy

Physical therapy should be considered in children with SA, especially in severe cases and if surgery is performed. [IIIB]

6. Surgery

1. Need for invasive procedures

Controversy remains regarding the need for invasive procedures besides diagnostic sampling. One prospective, and several retrospective studies have shown arthrocentesis to be appropriate approach for SA therapy in children also in hip and shoulder arthritis. [IIIB]

2. Indications for open drainage / arthrotomy

We suggest considering a more conservative approach to surgical treatment of paediatric septic arthritis once arthrocentesis has been performed [IIIC]. Arthrotomy is considered with childen under 3 months, or with nonneonatal children with a long duration of symptoms at presentation (>5 days), or patients that have failed conservative treatment or have undergone already ≥3 repeated aspirations without symptom alleviation. Arthroscopy is an option and provides visualization of the joint space. Routine placement of a drain is not supported by evidence. [IIIB]

7. Monitoring outcome

1. Risk of sequelae

The risk of sequelae is low in children when the diagnosis and management is performed early in the diseases. Risk factors related to sequelae may include young infants and newborns, infections caused by more virulent strains such as MRSA, *Salmonella* or PVL-positive strains, longer duration of symptoms before therapy, and hip involvement. Thus, children with SA with any of these risk factors should be followed more closely and for a longer periods of time to rule out or treat sequelae. [IIB]

2. Monitoring recovery

A multidisciplinary team should follow children with SA until osteoarticular function is restored and sequelae are ruled out or resolved. In the short term, improvement of symptomatology and decrease of CRP may suffice to evaluate the appropriate outcome of children with uncomplicated SA. [IA]

3. Imaging

Controlled imaging studies may be of help in certain cases, especially in complicated infections or upon suspecting complication, usually manifesting as non-responce to treatment. At least hip arthritis may followed with X-ray imaging. [IIIB]

4. Duration of follow-up

As complications develop slowly, a long routine follow-up of at least a year is recommended. Routine follow-up by orthopedics or pediatricians with experience in pediatric musculoskeletal diseases may are recommended at 2-4 weeks, 3 months and 12 months after discharge or until the patient has resumed normal activities and is free of pain. Hip arthritis may be followed for a longer period. [IIIA]

Notes:

-Quality of evidence
I = Good evidence: Randomized placebo-controlled trials, randomized trials
II = Moderate evidence: Well designed not randomized studies, cohort, case control
III = Poor evidence: Expert opinion, case series

-Strength of recommendation - team consensus
A = Strong recommendqtion
B = Moderate recommendation
C = Weak recommendation

8. Literature review

For the full literature review including evidence grading and highlight on bias see Appendix I.

9. Key reference list *(for complete reference list see Appendix I)*

1. Saavedra-Lozano J, Falup-Pecurariu O, Faust SN, Girschick H, Hartwig N, Kaplan S, et al. Bone and Joint Infections. Pediatr Infect Dis J. 2017 Aug;36(8):788-799.

2. Nelson JD. Antibiotic concentrations in septic joint effusions. N Engl J Med. 1971 18;284:349-53.

3. Feigin RD, Pickering LK, Anderson D, Keeney RE, Shackleford PG. Clindamycin treatment of osteomyelitis and septic arthritis in children. Pediatrics. 1975 Feb;55(2):213-23.

4. Peltola H, Pääkkönen M, Kallio P, Kallio MJ; OM-SA Study Group. Clindamycin vs. first-generation cephalosporins for acute osteoarticular infections of childhood-a prospective quasi-randomized controlled trial. Clin Microbiol Infect. 2012 Jun;18(6):582-9

5. Arrieta AC, Bradley JS, Popejoy MW, et al. Randomized Multicenter Study Comparing Safety and Efficacy of Daptomycin Versus Standard-of-care in Pediatric Patients With Staphylococcal Bacteremia. Pediatr Infect Dis J. 2018; 37(9):893-900.

6. Thwaites GE, Scarborough M, Szubert A. Adjunctive rifampicin for Staphylococcus aureus bacteraemia (ARREST): a multicentre, randomised, double-blind, placebo-controlled trial. Lancet. 2018; 391: 668-678.

7. Pääkkönen M, Kallio Pe, Kallio MJI, Peltola H. Does bacteremia associated with bone and joint infections necessitate prolonged parenteral antibiotic therapy. J Ped Infect Dis Soc 2015;4:174-7

8. Peltola H, Pääkkönen M, Kallio P, Kallio MJ; Osteomyelitis-Septic Arthritis (OM-SA) Study Group. Prospective, randomized trial of 10 days versus 30 days of antimicrobial treatment, including a short-term course of parenteral therapy, for childhood septic arthritis. Clin Infect Dis. 2009 May 1;48(9):1201-10. doi: 10.1086/597582.

9. Gijón M, Bellusci M, Petraitiene B, et al. Pediatric Community-Acquired Bone and Joint Staphylococcus Aureus Infections In Europe: Severe Infections are Associated to Panton-Valentine Leucocidin Presence. Pediatr Infect Dis J. 2020 Jun;39(6):e73-e76.

10. Chen CJ, Chiu CH, Lin TY, et al. Experience with linezolid therapy in children with osteoarticular infections. Pediatr Infect Dis J. 2007 Nov;26(11):985-8.

11. Messina AF, Namtu K, Guild M, Dumois JA, Berman DM. Trimethoprim-sulfamethoxazole therapy for children with acute osteomyelitis. Pediatr Infect Dis J. 2011;30(12):1019–21.

12. Delgado-Noguera MF, Forero Delgadillo JM, Franco AA, Vazquez JC, Calvache JA. Corticosteroids for septic arthritis in children. Cochrane Database Syst Rev. 2018; 21;1:CD012125.

13. Smith SP, Thyoka M, Lavy CB, Pitani A. Septic arthritis of the shoulder in children in Malawi. A randomized, prospective study of aspiration versus arthrotomy and washout. J Bone Joint Surg Br. 2002; 84: 1167-72.

14. Givon U, Piberman B, Schindler A, Blankstein A, Ganel A. Treatment of septic arthritis of the hip joint by repeated ultrasound-guided aspirations. J Pediatr Orthop 2004; 24: 266-70.

15. Grammatico-Guillon L, Maakaroun Vermesse Z, Baron S, Gettner S, Rusch E, Bernard L. Paediatric bone and joint infections are more common in boys and toddlers: a national epidemiology study. Acta Pediatr 2013; 102: e120-5.

10. Literature search strategy

A literature search was performed seeking randomized controlled trials, prospective trials on comparative retrospective series focusing on arthrocentesis or more extensive surgery in the treatment of pediatric septic arthritis. References of available reports were checked to find further references.

11. Appendix

1. Appendix I. Comprehensive review regarding EBJIS septic arthritis guideline (2021) workgroup 11, pediatric septic arthritis.
